# Finding branched pathways in metabolic network via atom group tracking

**DOI:** 10.1371/journal.pcbi.1008676

**Published:** 2021-02-02

**Authors:** Yiran Huang, Yusi Xie, Cheng Zhong, Fengfeng Zhou

**Affiliations:** 1 School of Computer and Electronics and Information, Guangxi Key Laboratory of Multimedia Communications and Network Technology, Guangxi University, Nanning, China; 2 College of Computer Science and Technology, Key Laboratory of Symbolic Computation and Knowledge Engineering of Ministry of Education, Jilin University, Changchun, China; La Jolla Institute for Allergy and Immunology, UNITED STATES

## Abstract

Finding non-standard or new metabolic pathways has important applications in metabolic engineering, synthetic biology and the analysis and reconstruction of metabolic networks. Branched metabolic pathways dominate in metabolic networks and depict a more comprehensive picture of metabolism compared to linear pathways. Although progress has been developed to find branched metabolic pathways, few efforts have been made in identifying branched metabolic pathways via atom group tracking. In this paper, we present a pathfinding method called BPFinder for finding branched metabolic pathways by atom group tracking, which aims to guide the synthetic design of metabolic pathways. BPFinder enumerates linear metabolic pathways by tracking the movements of atom groups in metabolic network and merges the linear atom group conserving pathways into branched pathways. Two merging rules based on the structure of conserved atom groups are proposed to accurately merge the branched compounds of linear pathways to identify branched pathways. Furthermore, the integrated information of compound similarity, thermodynamic feasibility and conserved atom groups is also used to rank the pathfinding results for feasible branched pathways. Experimental results show that BPFinder is more capable of recovering known branched metabolic pathways as compared to other existing methods, and is able to return biologically relevant branched pathways and discover alternative branched pathways of biochemical interest. The online server of BPFinder is available at http://114.215.129.245:8080/atomic/. The program, source code and data can be downloaded from https://github.com/hyr0771/BPFinder.

## Author summary

Computational search of branched metabolic pathways is a fundamental problem in metabolic engineering and metabolic network analysis, which provides a systematic way of understanding the metabolism and discovering alternative pathways for synthesis of useful biomolecules. We propose BPFinder, a novel computational approach to identify branched metabolic pathways via atom group tracking. Different from other pathfinding methods using atom tracking, BPFinder tracks the movement of atom groups in metabolic network to find linear atom group conserving pathways, and merge the found linear pathways by the selected branched compounds to generate branched pathways. Based on the structure of conserved atom groups in branched compounds, we design two merging rules for branched compounds: overlapping rule and non-overlapping rule. The user can flexibly adopt these rules to accurately find the branched pathways that contain overlapping/non-overlapping conserved atom groups. BPFinder also enables the user to combine the information of compound similarity, Gibbs free energy of reactions, and conserved atom groups to sort resulting pathways. Compared with other existing methods, BPFinder can more accurately recover the known branched pathways. The alternative branched pathways returned by BPFinder reveal that the user can flexibly utilize our proposed merging rules to discover biochemically meaningful pathways of interest.

## Introduction

In the past decades, the quantity of metabolic data in the metabolic databases, such as KEGG (Kyoto Encyclopedia of Genes and Genomes) [1–3] and MetaCyc [4], has a rapid explosion, which makes it possible to explore the metabolic networks in depth [5,6]. Research on this large amount of metabolic data requires new computational methods to automatically search and analyse biologically meaningful metabolic pathways. Many efforts have been devoted to automatically find metabolic pathways, which can be classified into stoichiometric methods and graph-based pathfinding methods. Stoichiometric methods [7–11] typically establish stoichiometry optimization models to find the pathways that convert a source compound to a target compound in metabolic networks [12,13], and are usually applied for modelling specific metabolic systems [14]. On the other hand, a metabolic network can be represented as a graph where the metabolites can be denoted as nodes and the reactions can be denoted as edges [15]. An intuitive strategy for finding metabolic pathways is to search pathways based on the connectivity of the reactions and the metabolites in the graph. Previous graph-based pathfinding methods [16–24] dominantly focused on finding linear metabolic pathways between a pair of source and target compounds in metabolic networks.

Nevertheless, a graph-based pathfinding strategy [16–21] sometimes may involve hub metabolites into the resulting pathways as it selects reactions and compounds based on the connectivity to find linear pathways [25]. Recently, people find that tracking the movements of atoms from the source compound to the target compound is an effective way of avoiding hub metabolites in finding linear pathways [26]. A number of atom tracking methods, such as LPAT [26], MetaRoute [27], CFP [13], PathTracer [28], HPAT [29] and RouteSearch [22] have been successfully proposed to avoid hub metabolites when finding linear metabolic pathways.

However, these approaches require defining the atoms to be tracked. This may result in missing of the pathways that do not conserve the tracked atoms. For solving this problem, based on the KEGG RPAIR database [3,30], we proposed a linear pathfinding method, namely AGPathFinder [31] for finding linear metabolic pathways by atom group tracking. AGPathFinder searches the linear pathways by tracking the movement of atom groups through metabolic network, and combines the information of reaction thermodynamics and compound similarity to direct the search of linear metabolic pathways in the KEGG RPAIR database. Similarly, Faust et al.[32] constructed weighted metabolic network by the reactant pairs of KEGG RPAIR, and inferred pathways between a set of compounds or reactions in the weighted metabolic network, and evaluated the effects of the weighting and filtering of reactant pairs in pathfinding. The evaluation results showed that combining RPAIR annotation with compound weighting can greatly improve the quality of pathfinding [32].

On the other side, branched metabolic pathways consist of multiple pathways that biochemically interact in metabolic networks. Comparing with linear metabolic pathways, the branched metabolic pathways are dominant in metabolic networks and describe a more comprehensive picture of metabolism [14,33]. Identifying branched metabolic pathways enables the analysis of metabolism with a complete insight in comparison to the limited picture described by linear pathways [14].

A number of pathfinding methods have been proposed for finding branched metabolic pathways. For example, Gerard.et al [34] developed a bio-inspired algorithm PhDSeeker for finding feasible linear and branched metabolic pathways using ant colony optimization algorithm. Aarthi Ravikrishnan.et al [24] presented a pathfinding approach MetQuest for identifying branched metabolic pathways by combining breadth-first search with dynamic programming. Heath.et al [26] proposed a graph-based method BPAT-S for finding branched metabolic pathways using atom tracking. BPAT-S utilizes LPAT to return a set of linear metabolic pathways between a pair of source and target metabolites. And these linear branches are attached to the seed pathway to produce branched pathways. Another graph-based method ReTrace [33] takes a similar strategy to give rise to branched pathways, but the linear pathways of ReTrace only conserve one atom. Based on the observation that BPAT-S takes a lot of time to find branches and these branches may have already been involved in the linear pathways obtained by LPAT, Heath.et al [14] proposed another branched pathfinding method BPAT-M to eliminate this redundancy by comprehensively inventorying the linear pathways returned by LPAT and merge the linear pathways using atom tracking information.

**Fig 1:**
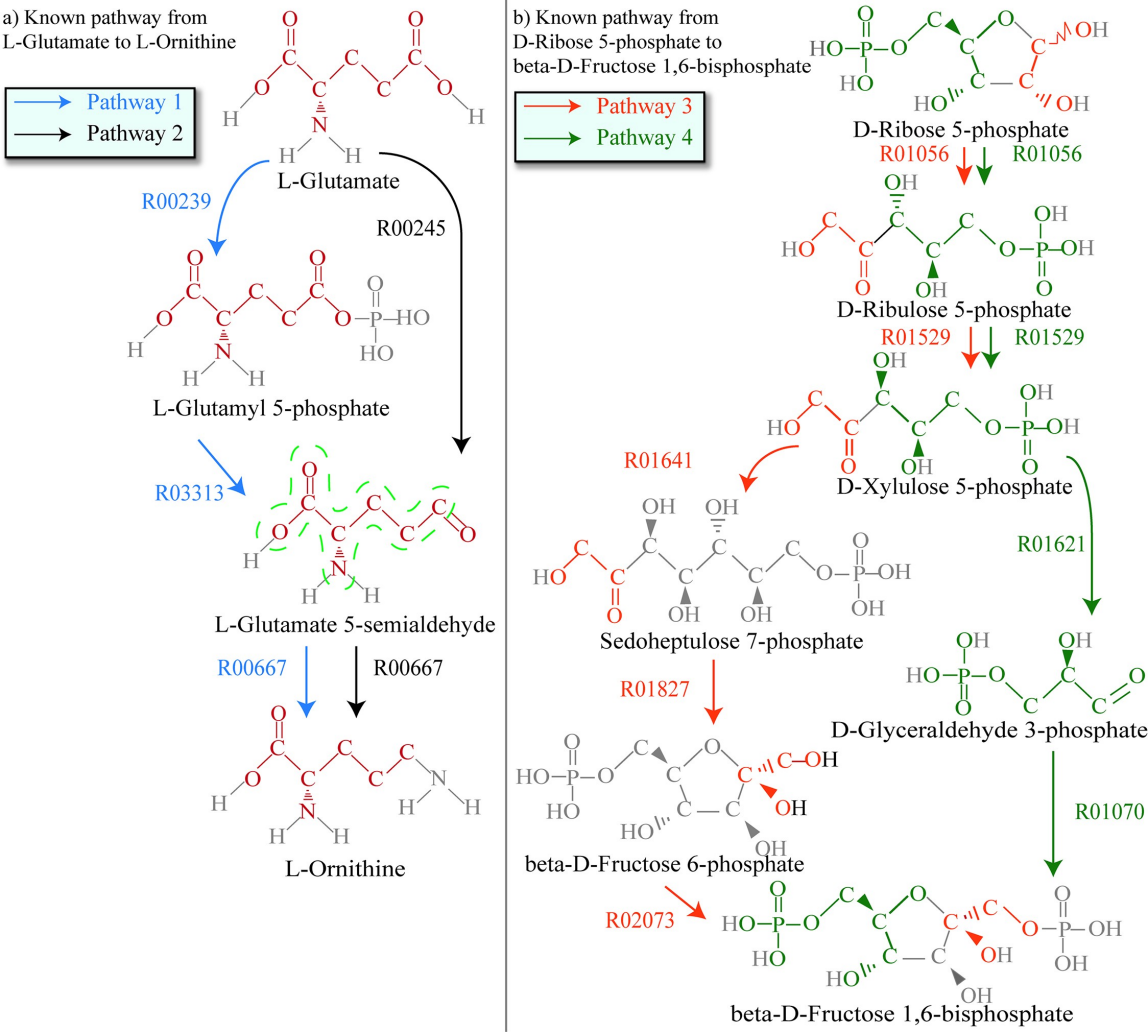
Two branched metabolic pathways. **a)** A part of the branched metabolic pathway rn00330 from L-Glutamate to L-Ornithine in KEGG. **b)** A part of the branched metabolic pathway rn00030 from D-Ribose 5-phosphate to beta-D-Fructose 1,6-bisphosphate in KEGG. The conserved atom groups transferred from source compound to current compound are drawn in red for pathways 1 and 2. The conserved atom groups transferred from source compound to current compound are drawn in orange and green for pathways 3 and 4 respectively. The partition circled with green dashed line in the compound L-Glutamate 5-semialdehyde is the overlapping part of the conserved atom groups of pathways 1 and 2. Note that, in this paper, the compounds and reactions are represented by their KEGG identifiers.

Branched compound is the compound that acts as branch point to connect multiple linear pathways in metabolic network. It is known that, in many known branched metabolic pathways, linear pathways contain overlapping conserved atom (groups) in branched compounds. For example, Fig 1A shows a part of the branched metabolic pathway rn00330 of *Homo sapiens* from L-Glutamate to L-Ornithine in KEGG database. This branched pathway consists of the linear pathways 1 and 2. As shown in Fig 1A, the pathway 1 and the pathway 2 contain overlapping conserved atom groups in branched compound L-Glutamate 5-semialdehyde (the overlapping conserved atom groups in the compound L-Glutamate 5-semialdehyde of pathways 1 and 2 are circled with green dashed line in Fig 1A).

On the other hand, some linear pathways in the known branched pathways contain non-overlapping conserved atom (groups) in branched compounds. For example, Fig 1B shows a part of the branched pathway rn00030 of *Quercus suber* from D-Ribose 5-phosphate to beta-D-Fructose 1,6-bisphosphate in KEGG. This branched pathway consists of linear pathways 3 and 4. As shown in Fig 1B, pathways 3 and 4 contain non-overlapping conserved atom groups in branched compound D-Xylulose 5-phosphate (the non-overlapping conserved atom groups in the compound D-Xylulose 5-phosphate of pathways 3 and 4 are drawn in orange and green respectively in Fig 1B).

Branched pathway is a universal characteristic of metabolism[14]. From the parts of pathways rn00330 and rn00030 in Fig 1, we can observe that there exist two kinds of branched pathways: The branched pathway that contains overlapping conserved atom (groups) in branched compound, and the branched pathway that contains non-overlapping conserved atom (groups) in branched compound. Generally, branched pathways produce target compounds through combinations of linear pathways that split branched compounds into smaller ones, work parallel with many compounds, and join compounds into larger ones [14], which results in the emergence of overlapping/non-overlapping conserved atom (groups) in branched compounds. The ways of involving overlapping/non-overlapping conserved atom (groups) in branched compounds lead to these two classifications of branched pathways.

BPAT-M is an effective method for finding branched pathways containing non-overlapping conserved atoms in branched compound [14]. For example, BPAT-M successfully found an alternative branched pathway from α-D-Glucose 6-phosphate to cephalosporin C by merging linear pathways though branched compounds containing non-overlapping conserved carbon atoms [14]. In this found pathway, BPAT-M correctly identifies the crucial reaction catalyzed by d-(l-a-aminoadipyl)-l-cysteinyl-d-valine (ACV) synthetase in the known pathway, and implies that it would more likely use the glycolysis pathway to produce pyruvate in the pathway from α-D-Glucose 6-phosphate to l-valine [14]. The automatic findings of the reaction catalyzed by ACV synthetase and the alternative pathway from α-D-Glucose 6-phosphate to l-valine indicate that finding branched pathways containing non-overlapping conserved atoms in branched compound can correctly retrieve reactions in known pathway and find alternative pathways that perhaps may not be typically considered, which might provide useful implications for designing biosynthetic pathway of cephalosporin C [14].

However, BPAT-M only merges the linear pathways that do not contain overlapping conserved atoms in the branched and target compounds to produce branched pathways. This may result in failing to predict the branched pathways that contain overlapping conserved atoms in the branched and target compounds.

In general, the applications of pathfinding results may vary in particular scenarios. In the application like metabolic network reconstruction, it is usually desirable to search alternative metabolic pathways in specific organisms [3,14,19,35,36]. In the applications like synthetic biology and metabolic engineering, it is also desirable to search alternative pathways that do not exist in a single organism [14,19]. One of the primary goals of synthetic biology is to redesign metabolic pathways for producing desired compounds [19]. The emerging molecular biology techniques [37–39] have increasingly allowed to experimentally re-implement desired enzymes in any cellular context to draft a *de novo* synthetic pathway [19]. For example, a synthetic pathway has been experimentally re-implemented in yeast to produce noscapine from tyrosine [40]. In this synthetic pathway, totally 18 enzymatic steps, only 13 enzymes were from the opium poppy, and two were from the brown rat and bacteria, and the other three were from other plants [40]. In such experimental study, the bottleneck is transferring from the implementation of pathways to the initial design of pathways with the rapid development in molecular biology [19]. It is thus desirable to develop appropriate pathfinding tools for guiding the design of synthetic metabolic pathways that may not exist in a single organism [14,19,26]. A number of pathfinding methods including Tinker[19], BPAT-M[14], BPAT-S[26], LPAT[26] and HAPT [29] were developed to search pathways for the design of synthetic pathways that may span multiple organisms through the entire reactions in biochemical database.

In this work, we propose a pathfinding method called BPFinder for finding branched metabolic pathways between two given compounds by atom group tracking, which intends to assist in the synthetic design of metabolic pathways by considering the entire reactions in KEGG RPAIR database.

The contributions of our work are listed as follows.

- Different from the branched pathfinding methods using atom tracking, BPFinder identifies linear metabolic pathways by tracking atom groups, and merges the linear atom group conserving pathways to produce branched pathways. This can enable users to find branched metabolic pathways without defining the tracked atoms. The reactant pairs (RPAIRs) are the compound pairs that have atoms or atom groups in common between two sides of reaction [30]. Although the pathways computed by Faust et al.’s method [32] are identified in the metabolic network constructed by the reaction pairs of KEGG RPAIR and these computed pathways may include the compounds with conserved atom groups transferred between reactant pairs, the computed pathways of Faust et al.’s method are not required to include the compound with conserved atom groups transferred from source compound. And therefore the compound of the computed pathways from Faust et al.’s method [32] may possibly not include the conserved atom groups from source compound. Biochemical intuition implies that pathways that move a set of atoms from source to target compounds will be biochemically relevant [26]. Different from the pathways computed by Faust et al.’s method [32], our method infers pathways by tacking the movement of the conserved atom groups from source to target compound, and each compound of our computed pathway is required to include the conserved atom groups from source compound, which may facilitate us to discover biologically relevant pathways.
- In order to uncover the branched pathways that contain the linear pathways with overlapping conserved atom groups in the branched compounds, and provide a complete picture of the transformations of the conserved atom groups in the pathway inference, we propose two merging rules for branched compound based on the structure of the conserved atom groups: overlapping rule and non-overlapping rule. Overlapping rule merges the linear pathways that contain overlapping conserved atom groups in the branched compounds to produce pathways. Non-overlapping rule merges the linear pathways that contain non-overlapping conserved atom groups in the branched compounds to produce pathways. This can enable us to closely follow and analyze the confluence and separation of the conserved atom groups during the pathway inference, and discover potentially useful branched pathways.

Additionally, it is noted that weight schemes based on the characteristics such as reaction thermodynamics and compound similarity, can assist the user to pick out the metabolic pathways of interest [14,31]. The combined information of thermodynamic feasibility, branched compounds, compound similarity and conserved atom groups is also utilized together to rank the resulting branched pathways for the user. To facilitate the use of our framework, we also provide the web-based version (http://114.215.129.245:8080/atomic/) and pathway visualization results.

Experimental results conducted on the multi-genome scale data of KEGG demonstrate that our method can more accurately recover the known branched metabolic pathways than other existing methods, and is capable of finding biochemically relevant branched pathways of interest. Overall, BPFinder is a generally applicable and effective tool for finding branched metabolic pathways.

## Results

In this section, we verify the experimental performance of BPFinder on finding branched pathways in metabolic network. BPFinder is implemented in Java. We use 5848 compounds, 7340 reactions with atom mapping information obtained from the KEGG LIGAND database[2] to build metabolic network, and search the branched pathways converting atom groups from the given source to target compound in the built metabolic network.

BPAT-S [26], BPAT-M [14], PhdSeeker [34], Retrace [33] and MetQuest [24] are five available algorithms that are able to find branched metabolic pathways, and we choose these five branched pathfinding algorithms as the baselines to evaluate the performance of BPFinder on finding branched metabolic pathways. In the experiments, we evaluate the performance of the pathfinding methods by comparing the resulting branched pathways with 30 known branched pathways (see S1 Text) retrieved from the literature [26,31,41] and the KEGG database [24,31]. Note that, these 30 known pathways are retrieved from core branched metabolic pathways that may span multiple organisms in KEGG, and the structures of these pathways may perhaps be largely conserved across domains of life. In order to learn the performance on finding pathways whose structure may not largely be conserved across domains of life, we also evaluate the performance of the pathfinding methods by comparing the resulting branched pathways with 20 known organism-specific branched pathways (see S3 Text and S4 Text) that are retrieved from KEGG [24,31] and only exist in less than 20% of 3312 organisms in KEGG. Moreover, three found branched pathways will be discussed to learn more about the features of the branched pathfinding methods.

BPAT-S and BPAT-M are web-based frameworks. We used BPAT-S and BPAT-M to find branched pathways on http://metabolicpaths.kavrakilab.org/#bpatsrun and http://metabolicpaths.kavrakilab.org/#bpatmrun, respectively. Retrace [33], PhdSeeker [34] and MetQuest [24] are downloaded from https://www.cs.helsinki.fi/group/sysfys/software/retrace/, https://sourceforge.net/projects/sourcesinc/files/phdseeker/, and https://github.com/RamanLab/metquest respectively. BPFinder, Retrace, PhdSeeker and MetQuest were run on the computer with an Intel Xeon CPU 6130 and 40GB RAM. The running operating system is Linux.

### Performance metrics of branched pathfinding

In this work, we evaluate the biochemical relevance of the computed branched pathways by comparing the computed branched pathways with the known branched pathways. The experimental comparisons are carried out based on the following seven criteria.

- The Ratio of the Edges of the Largest Common Connected Sub-graph(*R*_*ELCCS*_): In the sub-graphs of the computed pathway, the sub-graph *G*_*sub*_ is the largest connected sub-graph that is isomorphic to a sub-graph of the known pathway, and *R*_*ELCCS*_ is the ratio of the edges of *G*_*sub*_ to the edges of the known pathway [42]. We can use *R*_*ELCCS*_ to evaluate the structure similarity of the computed pathway and known pathway. Higher *R*_*ELCCS*_ of the computed pathway indicates that the structure of the computed pathway is closer to the known pathway, which demonstrates the ability of recovering known pathways for pathfinding methods. The average value of *R*_*ELCCS*_ is computed by the following equation:
  $$R_{ELCCS}=\frac{1}{N}\sum_{i=1}^{N}R_{{ELCCS}_{i}}$$
  where *N* is the number of computed pathways, $R_{{ELCCS}_{i}}$ is the *R*_*ELCCS*_ of the *i*th computed pathway.
- Sensitivity *Sn* = *tp*/(*tp* + *fn*) where true positive compounds (*tp*) are the compounds found in both computed and known pathways and the orders of these compounds in the computed and known pathways are the same [31,34]. False negative compounds (*fn*) are the compounds in the known pathway but not in the computed pathway [31,34].
- Positive prediction value *PPV* = *tp*/(*tp* + *fp*) where false positive compounds (*fp*) are the compounds not in the known pathway but in the computed pathway [31,34].
- Accuracy for compound *AC* = (*Sn* + *PPV*)/2. Higher compound accuracy of the computed pathways demonstrates that the ability of recovering the compounds in the known pathway for pathfinding method is better [31,34].
- Sensitivity for reaction *R_Sn* = *r_tp*/(*r_tp* + *r_fn*) where true positive reactions (*r_tp*) are the reactions found in both computed pathway and known pathway and the orders of these reactions in the computed and known pathway are the same. False negative reactions (*r_fn*) are the reactions in the known pathway but not in the computed pathway [31,34].
- Positive prediction value for reaction *R_PPV* = *r_tp*/(*r_tp* + *r_fp*) where false positive reactions (*r_fp*) are the reactions not in the known pathway but in the computed pathway [31,34].
- Accuracy for reaction *R_AC* = (*R_Sn* + *R_PPV*)/2. Higher reaction accuracy of the computed pathways demonstrates that the ability of recovering the reactions in the known pathway for pathfinding method is better [31,34].

### Comparison with other methods

For each pair of source and target compounds of 30 known core branched pathways and 20 known organism-specific branched pathways, we use BPFinder, Retrace, BPAT-S, BPAT-M, PhDSeeker and MetQuest to find top five pathways, and compare the found pathways to the known pathways.

The parameters *α*_*s*_, *α*_*sf*_, *α*_*t*_ and *α*_*p*_ are weight parameters of BPFinder, which are used to adjust the relative weights of compound similarity, Gibbs free energy of reaction, conserved atom groups and branched compounds in pathfinding respectively (see section “Sorting branched metabolic pathways” for more details). In this section, the parameters in BPFinder are listed as follows: the number *k* of candidate linear pathways is 2000, *α*_*s*_ is 0.1, *α*_*sf*_ is 0.2, *α*_*t*_ is 0.2, *α*_*p*_ is 0.8 and the number of minimal atom groups transferred from source to target compound is 2. Meanwhile, BPFinder combines the overlapping and non-overlapping rules to search branched pathways. BPFinder first searches the branched pathways by non-overlapping rule, and then BPFinder searches the branched pathways by overlapping rule in the case of no branched pathways are returned by non-overlapping rule. The parameters in other comparative pathfinding methods are set to the default values (see S2 Text).

Table 1 summarizes the number of found branched pathways and the average *R*_*ELCCS*_, sensitivity, positive prediction value and accuracy for the 30 known core branched pathways tested.

**Table 1:** The number of found branched pathways and the average *R*_*ELCCS*_, sensitivity, positive prediction value and accuracy for the 30 known core branched pathways tested.

Method	Top Pathway							Best of top five pathways							Number of found branched pathways
	*R*_*ELCCS*_	*Sn*	*PPV*	*AC*	*R_Sn*	*R_PPV*	*R_AC*	*R*_*ELCCS*_	*Sn*	*PPV*	*AC*	*R_Sn*	*R_PPV*	*R_AC*	
BPFinder	0.222	0.26	0.29	0.275	0.134	0.157	0.145	0.237	0.276	0.309	0.293	0.134	0.157	0.145	103
Retrace	0.122	0.113	0.196	0.154	0.067	0.09	0.078	0.191	0.18	0.322	0.251	0.067	0.09	0.078	101
BPAT-S	0.181	0.201	0.154	0.177	0.103	0.118	0.111	0.204	0.212	0.163	0.187	0.103	0.118	0.111	141
BPAT-M	0.104	0.108	0.127	0.117	0.039	0.089	0.064	0.145	0.143	0.177	0.16	0.039	0.089	0.064	95
MetQuest	0.114	0.098	0.098	0.098	0.056	0.198	0.127	0.118	0.098	0.098	0.098	0.056	0.198	0.127	47
PhdSeeker	0.034	0.014	0.015	0.015	0.006	0.07	0.038	0.054	0.049	0.016	0.032	0.006	0.07	0.038	30

Note: The best performer is marked in red box presentation.

As can be seen in Table 1, BPAT-S returns more branched pathways than other methods. On the other hand, as shown in Table 1, for the top pathway and the best of top five pathways computed by each method, BPFinder performs the best with the highest values of *R*_*ELCCS*_ as compared to other comparative methods, and the performance of BPAT-S is comparable with the performance of BPFinder. These results indicate that the structures of the found branched pathways of BPFinder and BPAT-S are more similar to the known core branched pathways, and BPFinder is thus more capable of recovering the known core branched pathways than other comparative methods.

Moreover, as can be seen from Table 1, for the top pathway computed by each method, our BPFinder method outperforms other comparative methods not only in *Sn* but also in *PPV*, thereby resulting in a superior performance in overall *AC*. Furthermore, in Table 1, it can be seen that for the performance of the best of top five pathways, BPFinder also achieves higher values of *Sn* and *AC* than other methods, and Retrace obtains a higher *PPV* than other methods as it includes fewer false positive compounds in the resulting pathways. However, for the best of top five pathways, since more false negative compounds are also included in the resulting pathways of Retrace, the *Sn* of Retrace is much smaller as compared to BPFinder, which thus yields a lower *AC*. These results demonstrate that compared with other branched pathfinding methods, the ability of BPFinder in recovering the compounds of the known core branched pathways is better.

With regard to the performance of including reactions in the computed pathways, it can also be seen from Table 1 that, for the top pathway computed by each method, the performance of BPFinder is better than other methods in terms of *R_Sn* and *R_AC*. Moreover, for the best of top five pathways computed by each method, BPFinder also shows improved performance than five other methods in the values of *R_Sn* and *R_AC*. As seen from Table 1, for the top pathway and the best of top five pathways, MetQuest achieves higher values of *R_PPV* than other methods since its resulting pathways include fewer false positive reactions, however, the values of *R_Sn* of MetQuest are much lower in comparison to BPFinder, which results in lower values of *R_AC* than BPFinder. These results demonstrate that compared with other branched pathfinding methods, in most cases, BPFinder is able to more accurately recover the reactions of the known core branched pathways.

On the other hand, Table 2 summarizes the number of found branched pathways and the average *R*_*ELCCS*_, sensitivity, positive prediction value and accuracy for the 20 organism-specific branched pathways tested.

**Table 2:** The number of found branched pathways and the average *R*_*ELCCS*_, sensitivity, positive prediction value and accuracy for the 20 organism-specific branched pathways tested.

Method	Top Pathway							Best of top five pathways							Number of found branched pathways
	*R*_*ELCCS*_	*Sn*	*PPV*	*AC*	*R_Sn*	*R_PPV*	*R_AC*	*R*_*ELCCS*_	*Sn*	*PPV*	*AC*	*R_Sn*	*R_PPV*	*R_AC*	
BPFinder	0.23	0.24	0.267	0.253	0.117	0.119	0.118	0.29	0.339	0.292	0.315	0.179	0.127	0.153	72
Retrace	0.106	0.119	0.179	0.149	0.011	0.01	0.01	0.169	0.147	0.286	0.216	0.038	0.081	0.059	100
BPAT-S	0.166	0.221	0.114	0.168	0.077	0.046	0.061	0.226	0.243	0.126	0.184	0.102	0.062	0.082	99
BPAT-M	0.096	0.123	0.082	0.103	0.009	0.019	0.014	0.164	0.183	0.157	0.17	0.042	0.072	0.057	65
MetQuest	0.128	0.091	0.149	0.12	0.041	0.156	0.098	0.14	0.091	0.149	0.12	0.059	0.169	0.114	42
PhdSeeker	0.05	0.026	0.018	0.022	0.018	0.053	0.035	0.05	0.026	0.018	0.022	0.018	0.053	0.035	25

Note: The best performer is marked in red box presentation.

As can be seen in Table 2, Retrace returns more branched pathways than other methods. Furthermore, as shown in Table 2, for top pathway and the best of top five pathways, BPFinder also shows improved performance than other methods in *R*_*ELCCS*_. This result demonstrates that the found pathways of BPFinder are more similar to known organism-specific pathways than other comparative methods as well. Moreover, as seen from Table 2, for top pathway and the best of top five pathways, BPFinder gives higher values of *Sn*, *PPV* and *AC* than other methods. These results indicate that BPFinder is also more capable of recovering the compounds of known organism-specific pathways than other comparative methods.

Regarding the performance of including reactions in the computed pathways, as shown in Table 2, BPFinder shows improved performance in comparison to other methods in terms of *R_Sn* and *R_AC*, while MetQuest obtains higher values of *R_PPV* than other methods, however, the values of *R_Sn* of MetQuest are much smaller than BPFinder, which leads to lower values of *R_AC* than BPFinder. These results demonstrate that, in most cases, BPFinder is also able to more accurately recover the reactions of known organism-specific pathways than other methods.

Consequently, the results from Tables 1 and 2 demonstrate that BPFinder is able to discover biochemically relevant branched pathways and is an effective method for finding branched metabolic pathways.

### Sensitivity analysis of weight parameters

The parameters *α*_*p*_, *α*_*s*_, *α*_*sf*_ and *α*_*t*_ are weight parameters of BPFinder to adjust the relative weights of the number of branched compound, compound similarity, Gibbs free energy of reaction and the number of minimal atom group in sorting resulting pathways respectively. In the following, we will evaluate the impact of weight parameters *α*_*p*_, *α*_*s*_, *α*_*sf*_ and *α*_*t*_ on the performance of BPFinder. In order to independently evaluate the direct impact of each parameter on sorting resulting pathways, for each combination of parameters, we set one of the parameters *α*_*p*_, *α*_*s*_, *α*_*sf*_, *α*_*t*_ as 1 while the remaining three parameters are 0. For example, when (*α*_*p*_, *α*_*s*_, *α*_*sf*_, *α*_*t*_) = (0,1,0,0), we will only consider compound similarity to sort resulting pathways. Meanwhile, as in previous section, BPFinder also combines overlapping and non-overlapping rules to search branched pathways, and the number of candidate linear pathways is 2000.

Note that BPFinder produces branched pathways by merging linear atom group conserving pathways, and the pathways that do not transfer atom group from source to target compound will not be returned. The performances of BPFinder under different weight parameters are summarized in Tables 3 and 4.

**Table 3:** The average *R*_*ELCCS*_, sensitivity, positive prediction value and accuracy for the 30 known core pathways tested under different weight parameters.

Weight parameters	Number of minimal atom group	Top Pathway							Best of top five pathways						
		*R*_*ELCCS*_	*Sn*	*PPV*	*AC*	*R_Sn*	*R_PPV*	*R_AC*	*R*_*ELCCS*_	*Sn*	*PPV*	*AC*	*R_Sn*	*R_PPV*	*R_AC*
(*α*_*p*_,*α*_*s*_, *α*_*sf*_, *α*_*t*_) = (1,0,0,0)	Max	0.203	0.211	0.304	0.258	0.142	0.231	0.186	0.249	0.254	0.351	0.303	0.194	0.258	0.226
	Min	0.181	0.219	0.339	0.279	0.103	0.205	0.154	0.241	0.303	0.348	0.325	0.189	0.221	0.205
(*α*_*p*_,*α*_*s*_, *α*_*sf*_, *α*_*t*_) = (0,1,0,0)	Max	0.163	0.187	0.211	0.199	0.058	0.086	0.072	0.2	0.221	0.243	0.232	0.08	0.112	0.096
	Min	0.154	0.169	0.159	0.164	0.073	0.076	0.075	0.193	0.239	0.17	0.204	0.108	0.091	0.1
(*α*_*p*_,*α*_*s*_, *α*_*sf*_, *α*_*t*_) = (0,0,1,0)	Max	0.175	0.18	0.293	0.236	0.107	0.2	0.153	0.203	0.214	0.3	0.257	0.13	0.195	0.163
	Min	0.17	0.158	0.237	0.198	0.1	0.183	0.141	0.201	0.222	0.287	0.255	0.132	0.186	0.159
(*α*_*p*_,*α*_*s*_, *α*_*sf*_, *α*_*t*_) = (0,0,0,1)	Max	0.185	0.221	0.222	0.222	0.104	0.142	0.123	0.246	0.26	0.229	0.245	0.141	0.152	0.146
	Min	0.234	0.238	0.239	0.239	0.183	0.197	0.19	0.283	0.277	0.252	0.265	0.212	0.208	0.21

Note: The best performer is marked in red box presentation and the worst performer is marked in green box presentation. “Max” represents the number of minimal atom group transferred is maximum and “Min” represents the number of minimal atom group transferred is minimum.

**Table 4:** The average *R*_*ELCCS*_, sensitivity, positive prediction value and accuracy for the 20 known organism-specific pathways tested under different weight parameters.

Weight parameters	Number of minimal atom group	Top Pathway							Best of top five pathways						
		*R*_*ELCCS*_	*Sn*	*PPV*	*AC*	*R_Sn*	*R_PPV*	*R_AC*	*R*_*ELCCS*_	*Sn*	*PPV*	*AC*	*R_Sn*	*R_PPV*	*R_AC*
(*α*_*p*_,*α*_*s*_, *α*_*sf*_, *α*_*t*_) = (1,0,0,0)	Max	0.244	0.252	0.248	0.25	0.152	0.214	0.183	0.292	0.31	0.264	0.287	0.207	0.223	0.215
	Min	0.178	0.139	0.152	0.146	0.064	0.133	0.099	0.28	0.271	0.23	0.251	0.156	0.187	0.172
(*α*_*p*_,*α*_*s*_, *α*_*sf*_, *α*_*t*_) = (0,1,0,0)	Max	0.16	0.242	0.214	0.228	0.062	0.066	0.064	0.216	0.297	0.356	0.327	0.098	0.085	0.091
	Min	0.173	0.232	0.192	0.212	0.064	0.066	0.065	0.224	0.27	0.325	0.298	0.111	0.087	0.099
(*α*_*p*_,*α*_*s*_, *α*_*sf*_, *α*_*t*_) = (0,0,1,0)	Max	0.182	0.205	0.204	0.204	0.113	0.169	0.141	0.252	0.27	0.221	0.246	0.183	0.176	0.179
	Min	0.245	0.254	0.242	0.248	0.128	0.157	0.142	0.264	0.261	0.247	0.254	0.141	0.15	0.146
(*α*_*p*_,*α*_*s*_, *α*_*sf*_, *α*_*t*_) = (0,0,0,1)	Max	0.129	0.083	0.083	0.083	0.056	0.033	0.044	0.146	0.208	0.095	0.152	0.056	0.033	0.044
	Min	0.148	0.111	0.111	0.111	0.074	0.039	0.057	0.148	0.111	0.111	0.111	0.074	0.039	0.057

Note: The best performer is marked in red box presentation and the worst performer is marked in green box presentation. “Max” represents the number of minimal atom group transferred is maximum and “Min” represents the number of minimal atom group transferred is minimum.

As can be seen in Tables 3 and 4, most of the best performers are obtained from independently sorting resulting pathways by the parameters *α*_*p*_ and *α*_*t*_ respectively. Thus *α*_*p*_ and *α*_*t*_ could be important parameters that may greatly affect the performance of finding branched pathways. Totally 14 metrics, 8 best performers of the metrics including *AC*, *PPV*, *R_AC* and *R_PPV* in Table 3 and 10 best performers of the metrics including *AC*, *PPV*, *R_AC*, *R_Sn* and *R_PPV* in Table 4 are obtained when (*α*_*p*_, *α*_*s*_, *α*_*sf*_, *α*_*t*_) = (1,0,0,0). This demonstrates that, when we only consider the number of branched compound to sort resulting pathways, it would be easier to recover the compounds and reactions of known pathways in the resulting pathways.

Interestingly, we can see that in Tables 3 and 4, for the number of minimal atom group, both of the best and worst performers of the metrics could be produced by the parameters “Max” and “Min”. This may imply that tracking more minimal atom groups may not necessarily obtain better performance. Moreover, when (*α*_*p*_, *α*_*s*_, *α*_*sf*_, *α*_*t*_) = (0,0,0,1), 6 best performers of the metrics are obtained in Table 3 whereas 14 worst performers of the metrics are obtained in Table 4. These results demonstrate that, when we only consider the number of minimal atom group to sort resulting pathways, possibly due to different movements of conserved atom groups in the core and organism-specific pathways, the performances of BPFinder on searching core and organism-specific pathways are quite different, and it would be better to take into account such feature to find branched pathways by using the number of minimal atom group to sort resulting pathways.

On the other hand, it can also be seen that, 12 worst performers of the metrics including *R*_*ELCCS*_, *AC*, *PPV*, *R_AC*, *PPV*, *R_Sn* and *R_PPV* are obtained in Table 3 when (*α*_*p*_, *α*_*s*_, *α*_*sf*_, *α*_*t*_) = (0,1,0,0). This indicates that it would be difficult for BPFinder to return branched pathways that are similar to core metabolic pathways, and to recover the compounds and reactions of core metabolic pathways in the resulting pathways when we only consider compound similarity to sort resulting pathways.

Furthermore, 2 worst and 2 best performers of the metrics are obtained in Table 3 and Table 4 respectively when (*α*_*p*_, *α*_*s*_, *α*_*sf*_, *α*_*t*_) = (0,0,1,0), and most of the metrics obtained by (*α*_*p*_, *α*_*s*_, *α*_*sf*_, *α*_*t*_) = (0,0,1,0) in Tables 3 and 4 are between the best and worst performers. This demonstrates that we could obtain a moderate performance when we only consider the Gibbs free energy of reaction to sort resulting pathways.

Generally, the results of Tables 3 and 4 may provide us some implications on the choice of parameters. However, different parameter combinations may produce a variety of resulting pathways with interesting features, and it is up to the users to determine feasible parameters to search branched pathways with the feature of interest.

### Study case

The results in the previous section indicate that our method BPFinder is capable of finding biochemically relevant branched pathways by atom group tracking. BPAT-M, BPAT-S and Retrace are the available pathfinding methods that are able to search branched metabolic pathways using atom tracking. We will discuss three branched pathway cases to learn more about the characteristics of the branched pathfinding methods using atom (group) tracking. The purpose of this section is not to evaluate the performance of pathfinding methods, which have been discussed in the last section, but to obtain insight into the features of pathfinding methods by analysis.

#### Study case of branched pathway: Pyruvate to L-valine

**Fig 2:**
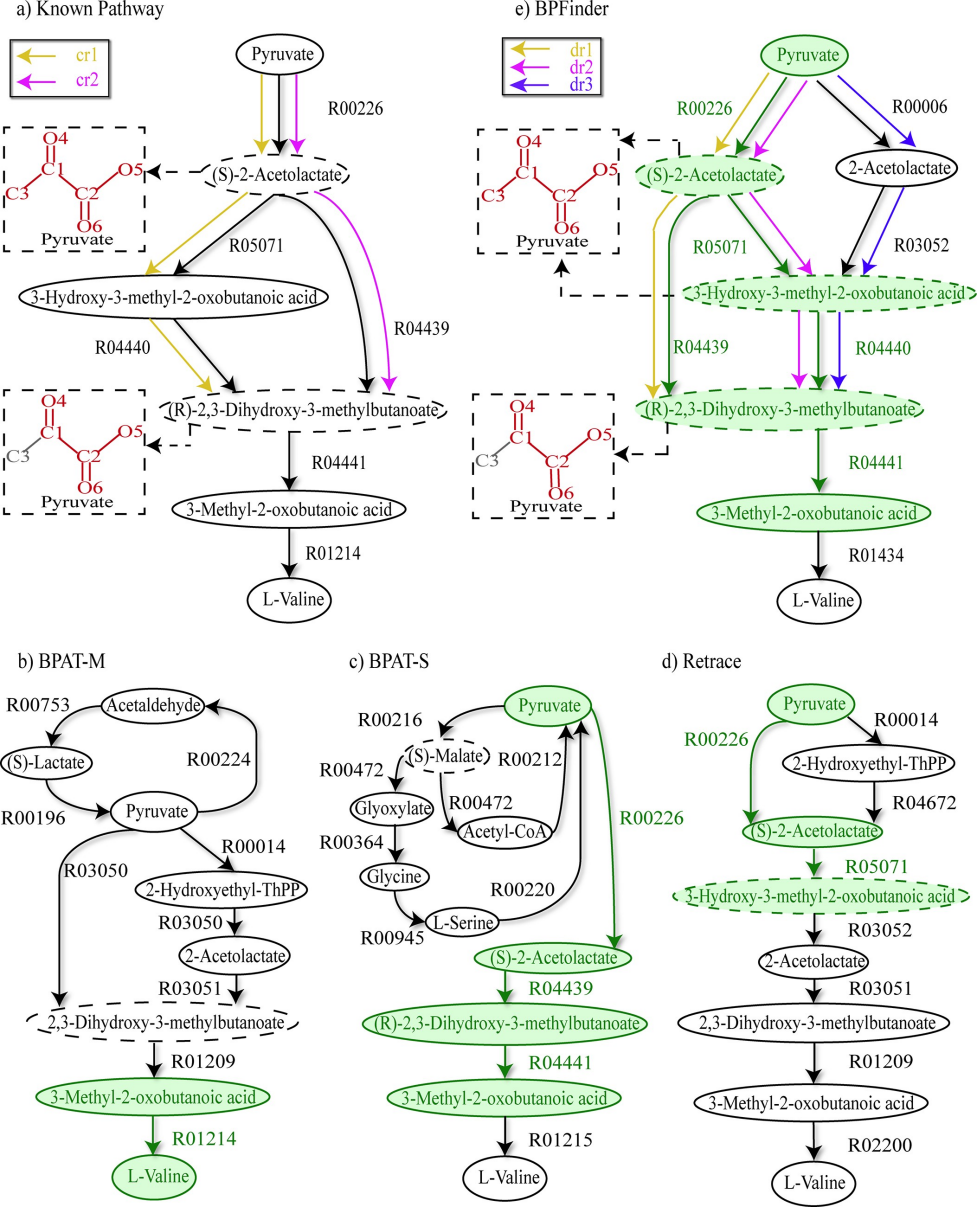
Branched pathway from pyruvate to L-valine. **a)** The known branched pathway from pyruvate to L-valine in KEGG. cr1, cr2 are two sub-branched pathways in this known pathway. **b)** The found pathway of BPAT-M. **c)** The found pathway of BPAT-S. **d)** The found pathway of Retrace. **e)** The found pathway of BPFinder. dr1, dr2, dr3 are three sub-branched pathways in the found pathway of BPFinder. The compounds circled with dashed ellipses are the branched compounds in the pathways. In the found pathways, the reactions that appear with the same substrates and products in the known pathway are drawn in green. The conserved atom groups from pyruvate in the branched compounds are drawn in red in the dashed rectangles.

L-valine is an essential amino acid for human being to maintain cell and organ protein content [43], and the biosynthesis pathway of L-valine can be started from pyruvate through a series of reactions catalyzed by acetohydroxy acid synthase, acetohydroxy acid isomeroreductase, dihydroxy acid dehydratase, and transaminase B [44]. Fig 2 shows the known branched pathway from pyruvate to L-valine in KEGG, and the branched pathways from pyruvate to L-valine found by BPAT-M, BPAT-S, Retrace and BPFinder. In the search of this branched pathway, BPFinder uses the overlapping rule to merge branched compounds to produce branched pathways, and the number of candidate linear pathways is 2000, the number of minimal atom groups transferred from pyruvate to L-valine is 2, and *α*_*s*_ is 0.1, *α*_*sf*_ is 0.2, *α*_*t*_ is 0.2, *α*_*p*_ is 0.8. The parameters in BPAT-M, BPAT-S and Retrace are set to the default values (see S2 Text).

In Fig 2B, we can see that BPAT-M merges two linear pathways from pyruvate to L-valine by the branched compound 2,3-Dihydroxy-3-methylbutanoate, and the last two compounds of the found pathway of BPAT-M are identical to those of the known pathway. As can be seen from Fig 2C, the linear part from pyruvate to 3-Methyl-2-oxobutanoic acid of the pathway found by BPAT-S is the same as the known pathway. Interestingly, in Fig 2B and 2C, it can be seen that both found pathways of BPAT-M and BPAT-S contain additional reactions and cycles that start from pyruvate.

In Fig 2D, the compounds pyruvate, (S)-2-Acetolactate, 3-Hydroxy-3-methyl-2-oxobutanoic acid, 3-Methyl-2-oxobutanoic acid and L-valine of the pathway returned by Retrace appear in the known pathway, but the rest compounds of this found pathway is different from the known pathway. These results demonstrate that atom tracking can enable BPAT-M, BPAT-S and Retrace to return the alternative branched pathways that are similar to the known branched pathway from pyruvate to L-valine to some extent.

On the other hand, in Fig 2E, the conserved atom groups in the branched compound (R)-2,3-Dihydroxy-3-methylbutanoate of dr1 and dr2 are the same, and BPFinder adopts overlapping rule to merge the sub-branched pathways dr1 and dr2 through (R)-2,3-Dihydroxy-3-methylbutanoate. BPAT-M cannot return the sub-branched pathways like dr1 and dr2 as BPAT-M only merges linear pathways through the branched compounds that do not contain overlapping conserved atoms to produce branched pathways. Similarly, neither of BPAT-S and Retrace combines the overlapping atoms in the branched compounds to produce branched pathways, and they do not return this branched pathway either.

Moreover, comparing Fig 2A and 2E, it can be seen that the sub-branched pathways dr1 and dr2 from BPFinder are the same as the sub-branched pathways cr1 and cr2 of the known pathway respectively. Noted that these two known branched pathways cr1 and cr2 appear in *E*. *coli* in KEGG and the conserved atom groups in the branched compounds (S)-2-Acetolactate and (R)-2,3-Dihydroxy-3-methylbutanoate of dr1 and dr2 are the same as those of cr1 and cr2 respectively. These results indicate that BPFinder successfully identifies the movement of the conserved atom groups in the known branched pathway and largely recovers the known branched pathway from pyruvate to L-valine by tracking atom groups. Note that this known branched pathway is commonly used in the biosynthesis of L-valine [44], BPFinder thus can be an generalized simple framework for finding branched pathways.

Besides the sub-branched pathways dr1 and dr2, BPFinder finds the third sub-branched pathway dr3, which goes through the pathway from pyruvate to (R)-2,3-Dihydroxy-3-methylbutanoate via the reactions R00006, R03052 and R04440. Note that, in Fig 2E, although the products of the reaction R00226 in dr2 and the reaction R00006 in dr3 are different, both reactions R00226 and R00006 can be catalyzed by the enzyme acetolactate synthase. Similarly, in dr2 and dr3, both reactions R05071 and R03052 can be catalyzed by the enzyme 2-acetolactate mutase. This indicates that the *de novo* synthesis of L-valine from pyruvate could involve different reactions catalyzed by the same enzyme, which might facilitate the experimental implementations of the *de novo* synthetic pathway *in vivo* or *in vitro*. These interesting relationships, discovered automatically by our method, could be helpful inspirations for the design and analysis of metabolic pathways.

#### Study case of branched pathway: Pregnenolone to 11beta-ydroxyprogesterone

**Fig 3:**
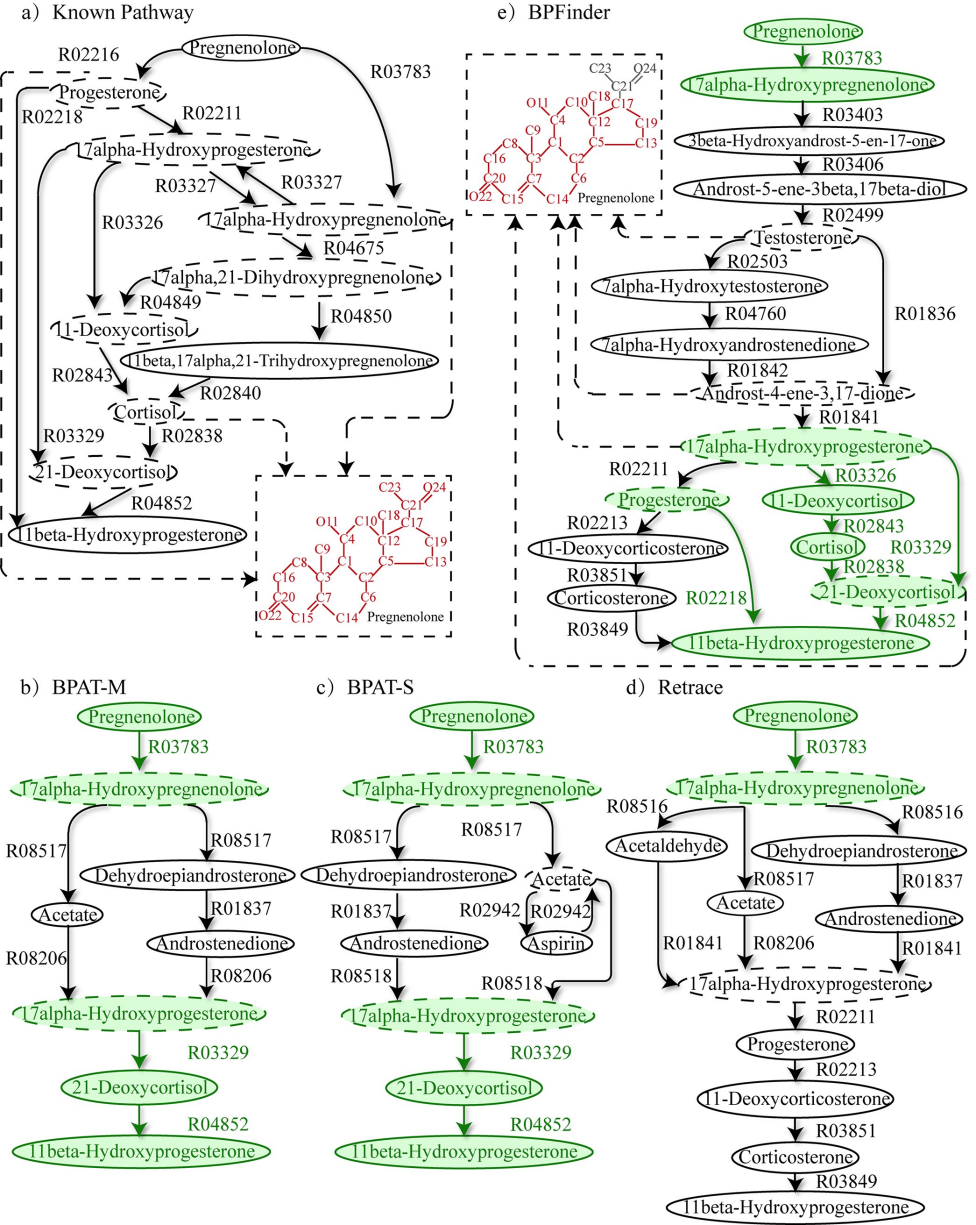
Branched pathway from pregnenolone to 11beta-Hydroxyprogesterone. **a)** The known branched pathway from pregnenolone to 11beta-Hydroxyprogesterone in KEGG. **b)** The found pathway of BPAT-M. **c)** The found pathway of BPAT-S. **d)** The found pathway of Retrace. **e)** The found pathway of BPFinder. The compounds circled with dashed ellipses are the branched compounds in the pathways. In the found pathways, the reactions that appear with the same substrates and products in the known pathway are drawn in green. The conserved atom groups from pregnenolone in the branched compounds are drawn in red in the dashed rectangles.

11beta-Hydroxyprogesterone influences the Na+ Absorption in mammalian principal cortical collecting duct cells [45] and is one of the important intermediates in the formation of aldosterone. 11beta-Hydroxyprogesterone can be synthesized from pregnenolone [46]. The *de novo* biosynthesis of 11beta-Hydroxyprogesterone from pregnenolone consists of many branched sub-pathways. Fig 3 shows the known branched pathway from pregnenolone to 11beta-Hydroxyprogesterone in KEGG, the branched pathways from pregnenolone to 11beta-Hydroxyprogesterone found by BPAT-M, BPAT-S, Retrace and BPFinder. Note that, the aim of study case is not to evaluate the performance of pathfinding methods, but to get insight into the characteristics of pathfinding. The branched pathways from pregnenolone to 11beta-Hydroxyprogesterone found by BPFinder using previous parameter values are only composed of 2 linear pathways. In order to learn more about the diversity of the branched pathways of BPFinder, in the search of this branched pathway, BPFinder applies overlapping rule to merge branched compounds to produce branched pathways, and finds a branched pathway composed of 4 linear pathways by the following parameters: the number of the candidate linear pathways is 2000, the number of minimal atom groups transferred from pregnenolone to 11beta-Hydroxyprogesterone is 3, and *α*_*s*_ is 0.3, *α*_*sf*_ is 0.2, *α*_*t*_ is 0.2, *α*_*p*_ is 0.8. The parameters in BPAT-M, BPAT-S and Retrace are set to the default values (see S2 Text).

In Fig 3B and 3C, we can see that five compounds pregnenolone, 17alpha-Hydroxypregnenolone, 17alpha-Hydroxyprogesterone, 21-Deoxycortisol, 11beta-Hydroxyprogesterone in the known pathway are included in the found pathway of BPAT-M and BPAT-S, and most of the compounds and reactions in the found branched pathway of BPAT-M and BPAT-S are the same. And in Fig 3D, except for the reaction R03783 and its substrate and product, the core part of the pathway from Retrace is different to the known pathway. Interestingly, four atom (group) tracking methods BPFinder, BPAT-M, BPAT-S and Retrace choose 17alpha-Hydroxypregnenolone and 17alpha-Hydroxyprogesterone to construct the found branched pathways. Although both 17alpha-Hydroxyprogesterone and 17alpha-Hydroxypregnenolone are contained in the known pathway and the found pathways from these pathfinding methods, the known pathway directly goes a shortcut from 17alpha-Hydroxypregnenolone to 17alpha-Hydroxyprogesterone through the reaction R03327 whereas 17alpha-Hydroxyprogesterone is created by different schemes with more reactions in these four computed pathways. This result reveals that 17alpha-Hydroxyprogesterone could be produced from 17alpha-Hydroxypregnenolone further upstream in the 11beta-Hydroxyprogesterone synthesis pathway.

In Fig 3E, based on 77 found linear pathways from pregnenolone to 11beta-Hydroxyprogesterone and 22 candidate branched compounds, BPFinder determines five overlapping branched compounds, and merges four linear pathways through these five branched compounds to build the branched pathways. From Fig 3E, it can be observed that, compared with the known pathway, one of the sub-branched pathways of BPFinder from 11-Deoxycortisol to 11beta-Hydroxyprogesterone appears in the known pathway. Pregnenolone is one of the source metabolites synthesizing testosterone and androst-4-ene-3,17-dione in the biosynthesis of steroids in rat brain [47]. Similar metabolic synthesis can also be observed in Fig 3E. In the pathway from BPFinder, pregnenolone is the source compound of synthesizing testosterone and androst-4-ene-3,17-dione.

The cultured human melanoma cells can metabolize progesterone to 11-deoxycorticosterone and corticosterone [48]. Meanwhile, the human melanoma line, which is shown to express *CYP17*, *CYP21A2*, *POMC* and *CRH* genes, could metabolize progesterone to corticosterone [48]. Interestingly, in Fig 3, BPFinder and Retrace not only find the sub-branched pathway metabolizing progesterone to corticosterone, but also return the reaction R03849 converting corticosterone to 11beta-hydroxyprogesterone. The reaction R03849 is catalyzed by the enzyme 21-hydroxylase that contains gene *CYP21A2* [49]. This implies the alternative pathway from progesterone to 11beta-hydroxyprogesterone could possibly be synthesized in the human melanoma line.

Although the found branched pathway from BPFinder is different from the known branched pathway, comparing the dashed rectangles of Fig 3A and 3E, we can find that the structures of the conserved atom groups in the branched compounds of the found pathway and the known pathway are very similar. The branched pathways with such characteristics may provide novel options and inspirations in designing *de novo* alternatives branched pathways for synthetizing 11beta-Hydroxyprogesterone.

#### Study case of branched pathway: D-Glucose 1-phosphate to 2-Phospho-D-glycerate

**Fig 4:**
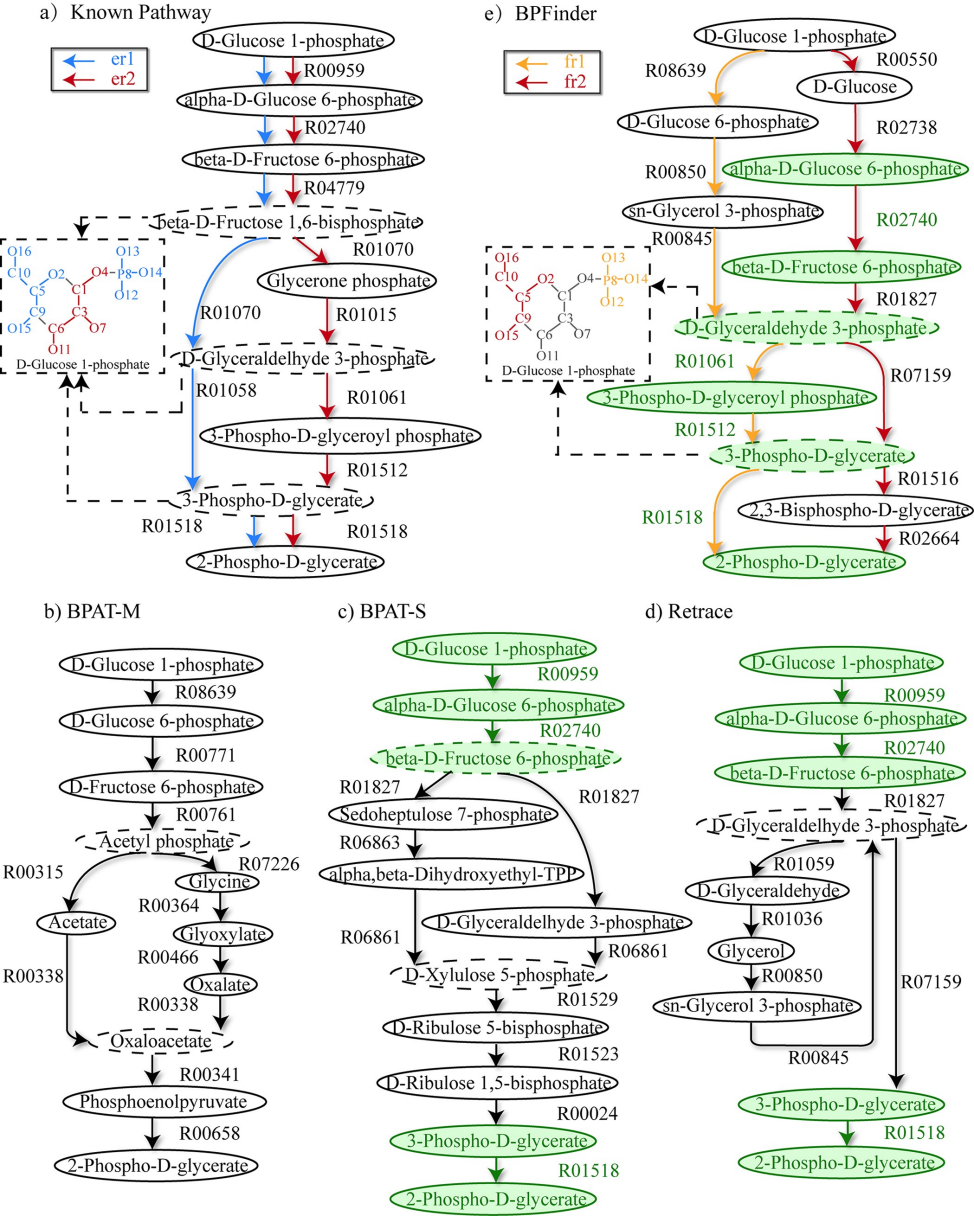
Branched pathway from d-Glucose 1-phosphate to 2-Phospho-D-glycerate. **a)** The known branched pathway from d-Glucose 1-phosphate to 2-Phospho-D-glycerate in KEGG. er1 and er2 are two sub-branched pathways in this known pathway. **b)** The found pathway of BPAT-M. **c)** The found pathway of BPAT-S. **d)** The found pathway of Retrace. **e)** The found pathway of BPFinder. fr1 and fr2 are two sub-branched pathways in the found pathway of BPFinder. The compounds circled with dashed ellipses are the branched compounds in the pathways. In the found pathways, the reactions that appear with the same substrates and products in the known pathway are drawn in green. In the sub-branched pathways er1 and fr1, the conserved atom groups from d-Glucose 1-phosphate in the branched compounds are drawn in blue and orange in the dashed rectangles respectively. In the sub-branched pathways er2 and fr2, the conserved atom groups from d-Glucose 1-phosphate in the branched compounds are drawn in red in the dashed rectangles.

2-Phospho-D-glycerate is an important intermediate of glycolysis and is valuable for the study of the enzymes, which use 2-Phospho-D-glycerate as a substrate, such as enolase and phosphoglycerate mutase [50]. Fig 4 shows the known branched pathway from d-Glucose 1-phosphate to 2-Phospho-D-glycerate in KEGG, and the branched pathways from d-Glucose 1-phosphate to 2-Phospho-D-glycerate found by BPAT-M, BPAT-S, Retrace and BPFinder. In this study case, we demonstrate how to use non-overlapping rule in BPFinder to discover the branched metabolic pathways that contain non-overlapping conserved atom groups in the branched compounds.

In the search of this branched pathway, different from the last two study cases, BPFinder adopts non-overlapping rule to merge branched compounds to generate branched pathways, and the number of the candidate linear pathways is 15000, the number of minimal atom groups transferred from d-Glucose 1-phosphate to 2-Phospho-D-glycerate is 3, *α*_*s*_ is 0.3, *α*_*sf*_ is 0.2, *α*_*t*_ is 0.2 and *α*_*p*_ is 0.8. The parameters in BPAT-S and Retrace are set to the default values (see S2 Text). BPAT-M does not return branched pathways for this study case by the default values. And in order to find this branched pathway by using BPAT-M, we readjust the parameters of BPAT-M to enlarge the search scope of branched pathways. And finally BPAT-M returns this branched pathway by the following parameters: The number of candidate linear pathways is 100000, the number of pathways in each cluster is limited to 2000, the beam search width for pathways is 500, and the minimal number of carbons for the seed pathways is 1.

In Fig 4B, we can find that compared with the known pathway, BPAT-M uses different compounds and reactions to build branched pathway, and uses two new branched compounds acetyl phosphate and oxaloacetate to construct this branched pathway. In Fig 4C, a part of the found pathway of BPAT-S from d-Glucose 1-phosphate to beta-D-Fructose 6-phosphate is the same as the known pathway, and BPAT-S finds two different sub-branched pathways starting from the branched compound beta-D-Fructose 6-phosphate to 2-Phospho-D-glycerate.

On the other hand, in Fig 4D, we can see that, the branched pathways from Retrace and BPAT-S are similar, and the first three compounds and the last two compounds in the branched pathways from Retrace and BPAT-S are the same. Additionally, the pathway from Retrace contains the critical reaction producing 2-Phospho-D-glycerate whereas also containing some additional reactions, compounds and cycles not found in the pathways of BPAT-M, BPAT-S and BPFinder.

From Fig 4E it can be observed that, the sub-branched pathways fr1 and fr2 consist of the branched pathway from BPFinder. And the conserved atom groups in the branched compound d-Glyceraldehyde 3-phosphate of fr1 and fr2 do not overlap with each other. Moreover, the conserved atom groups in the branched compound 3-Phospho-D-glycerate of fr1 and fr2 do not overlap with each other as well. Interestingly, similar scenarios can also be observed in the branched compounds of the sub-branched pathways er1 and er2 from the known pathway. And BPFinder adopts non-overlapping rule to merge fr1 and fr2 through the branched compounds d-Glyceraldehyde 3-phosphate and 3-Phospho-D-glycerate respectively.

In this found branched pathway, we can closely observe that the conserved atom groups transferred by fr1 and fr2 converge and separate at the branched compounds d-Glyceraldehyde 3-phosphate and 3-Phospho-D-glycerate during the inference of the branched pathways from d-Glucose 1-phosphate to 2-Phospho-D-glycerate. In the study of metabolic engineering, it is hard to formulate merely one “correct” branched metabolic pathway [14]. An important feature of our method is to return potentially useful branched pathways and enable the users to discover interesting alternative pathways. Discovering the characterized alternative pathways like this found pathway may be preferred to return the pathways that are already well studied in some applications [14].

## Discussion and conclusion

Computational identification of biochemically meaningful metabolic pathways is a crucial means for the applications such as metabolic network analysis, synthetic biology and metabolic engineering [14,17,26,32,33,51]. In this work, we propose a pathfinding method called BPFinder for finding branched metabolic pathways between two given compounds by atom group tracking, which aims to assist the design of synthetic metabolic pathways. Distinguishing from other branched pathfinding methods using atom tracking, BPFinder tracks the movement of atom groups in metabolic network to search linear pathways, and merge the linear atom group conserving pathways through the selected branched compounds to generate branched metabolic pathways. This enables the user to search biologically meaningful branched metabolic pathways without proposing the tracked atoms in advance.

During the selection of branched compounds for merging linear pathways, we provide two merging rules for branched compound based on the structure of conserved atom groups: overlapping rule and non-overlapping rule. We can flexibly use two rules on demand to accurately discover the branched metabolic pathways that contain overlapping/non-overlapping conserved atom groups in the branched compounds. This can facilitate us to closely observe the confluence and separation of the conserved atom groups in the branched metabolic pathways during the pathway inference. In order to provide more options for the user to find the branched pathways of interest, BPFinder also enables the user to combine the information of compound similarity, Gibbs free energy of reactions, and the conserved atom groups to sort the resulting branched pathways. We have shown the effectiveness of BPFinder by comparing the resulting branched metabolic pathways with the known branched metabolic pathways. The experimental results demonstrate that the ability of BPFinder to recover the known branched metabolic pathways is better than other existing pathfinding methods. The branched pathways found by BPFinder indicate that the user can flexibly use our proposed merging rules to find biologically meaningful branched pathways. BPFinder is available as an open-source tool and an on-line framework, which may facilitate further development and utility of the algorithm in such user-specified problem. BPFinder thus can be a promising general alternative to existing methods for finding branched metabolic pathways.

Metabolic pathfinding tools can help biochemists by producing a reasonable number of hypothetical pathways sorted by potential relevance, which might be modified and filtered based on biochemical knowledge and experimentally validated [32]. In application like synthetic biology, it could typically be desired to find alternative pathways that may span multiple organisms [14]. The found branched pathway of synthetizing 11beta-Hydroxyprogesterone from BPFinder in study case demonstrates how pathfinding tool can search alternative pathways that might be found in different organisms. And the ability of BPFinder in automatically searching different pathways that can produce the same compound could be of use in the areas such as synthetic biology or identifying drug targets [14]. In application like metabolic network reconstruction, it could be desired to find metabolic pathways that are similar to the known pathways existing in organism [14]. As seen in the study case, the branched pathway from BPFinder begins with pyruvate and proceeds through (S)-2-Acetolactate, 3-Hydroxy-3-methyl-2-oxobutanoic acid, and reaches 3-Methyl-2-oxobutanoic acid. This pathway is known to exist in *E*. *coli* and is commonly used in the biosynthesis of L-valine [44]. Although our method focuses on searching across the data in KEGG RPAIR, the automatic discovering of the branched pathway from pyruvate to 3-Methyl-2-oxobutanoic acid demonstrates that atom group tracking could also be applied to retrieve commonly used branched pathway known to exist in organism.

Despite its novel characteristics, BPFinder does not figure out a certain solution to all the challenges with regard to the *de novo* design of synthetic branched pathways. For example, similar to other pathfinding methods [14,19,26,29] for synthetic design of metabolic pathways, BPFinder focuses on searching potentially useful branched pathways without reference to any subcellular structure or specific organism. The returning pathways should be carefully carried out further analysis and study in depth before experimental implementations. Such implementations may involve recovering the found branched pathways *in vivo* or *in vitro*, which will increasingly become possible with the development of synthetic biology tools such as genetic switchboards [19,52].

Furthermore, in the procedure of merging linear pathways into branched pathways, it requires pathfinding methods to combine a large number of different linear pathways to generate branched pathways, which remains computationally challenging [21]. In order to control the complexity of the algorithm, BPFinder only produces branched pathways by merging linear pathways, which limits the search scope of branched pathways. In the future, a further extension of our method is to design a highly efficient algorithm for merging branched metabolic pathways, which would provide more comprehensive branched pathways for the analysis of metabolic processes. Moreover, only a source and a target compound are assigned as the input of BPFinder in searching branched metabolic pathways. Another possible future extension is to allow multiple source and target compounds as the input of the algorithm. We have concentrated on searching across the data in KEGG RPAIR, which appears database dependent and would potentially limit the generalizability of the tool. A possible future extension is to propose a feasible algorithm to extract atom mapping rules from the chemical reactions of various pathway databases, and we may search branched pathways in the metabolic networks of different databases based on the extracted atom mapping rules.

## Methods

**Fig 5:**
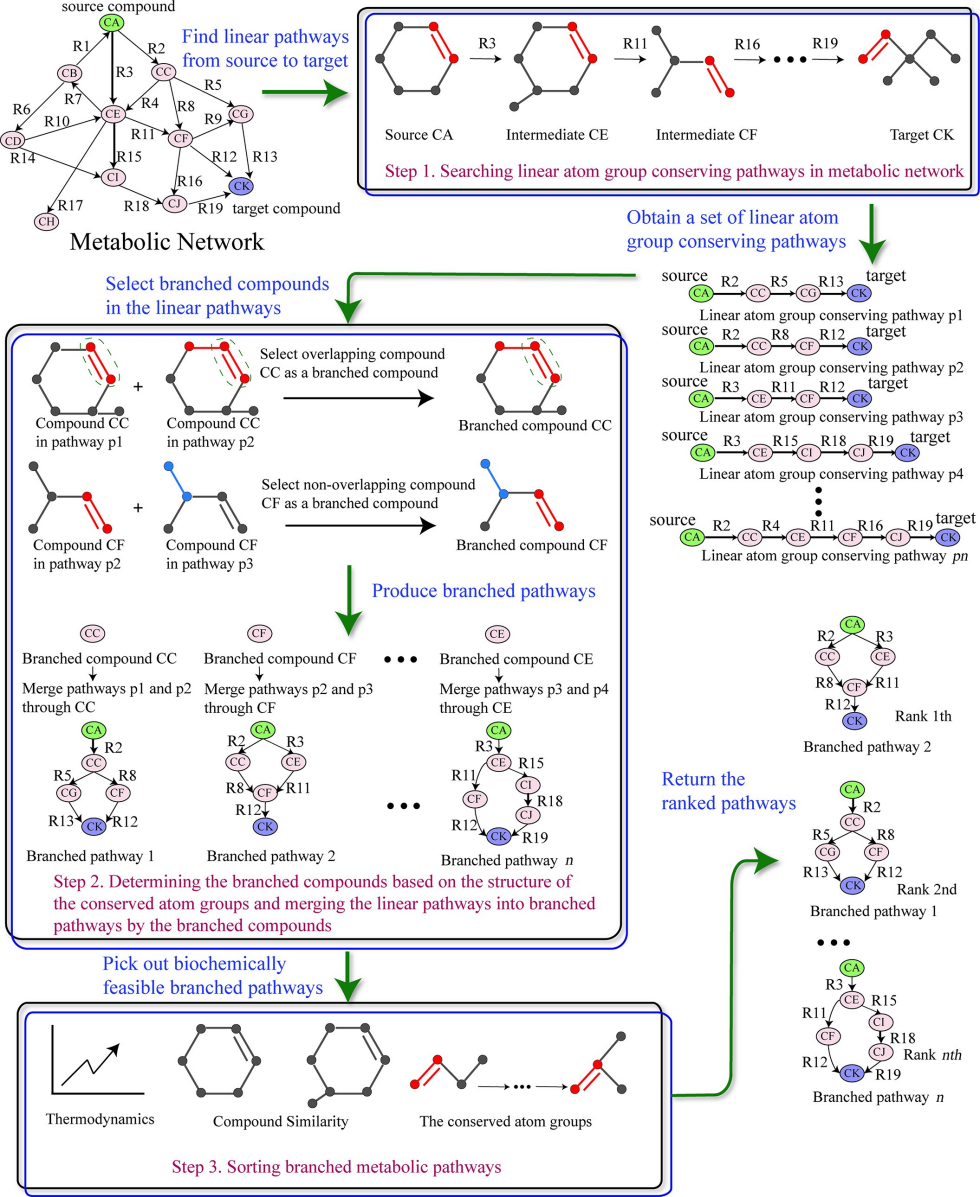
The overview of BPFinder. The compounds circled with ellipses are marked as CA-CK and the reactions are marked as R1-R19. The conserved atom groups are drawn in red or blue in the compounds.

Given a source compound and a target compound, our pathfinding method BPFinder finds the branched metabolic pathways converting the source to the target compound in three main steps: First, BPFinder finds linear pathways from source to target by tracking the movement of atom groups through metabolic network. Second, BPFinder determines the branched compounds in the linear pathways based on the structure of the conserved atom groups, and merges these linear pathways into branched pathways by the branched compounds. Finally, BPFinder sorts the resulting pathways by the combined information of reaction thermodynamics, compound similarity and the conserved atom groups to pick out biochemically feasible branched pathways for the user. The overview of our method BPFinder is summarized in Fig 5.

In the following, we will discuss our method BPFinder in detail.

### Searching linear atom group conserving pathways

**Fig 6:**
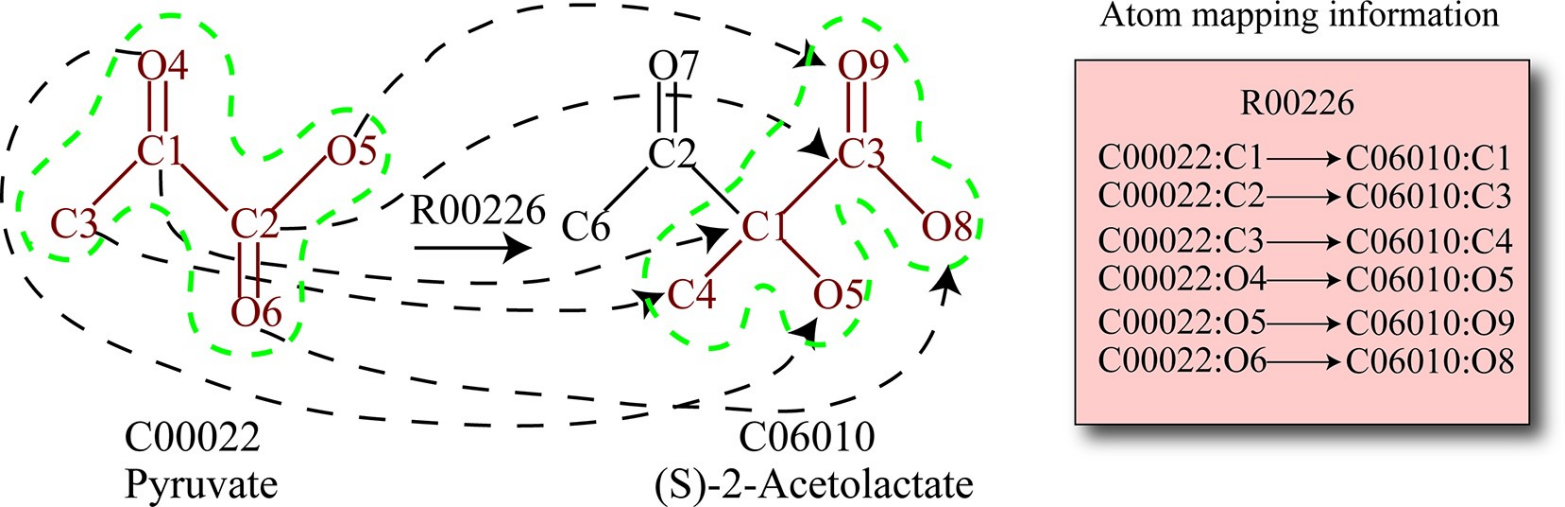
The atom group transferred from pyruvate to (S)-2-Acetolactate in reaction R00226. The dotted arrows denote the mapping of atoms from pyruvate to (S)-2-Acetolactate via R00226. The partition circled with green dashed line is the atom group transferred from pyruvate to (S)-2-Acetolactate in R00226. The rectangle on the right shows the atom mapping information between the compounds pyruvate and (S)-2-Acetolactate of R00226 in the KEGG RPAIR.

An atom group is a group of connected atoms moved from substrate to product in reaction, and the covalent bonds connecting the atoms in the group are conserved during the reaction [53]. For example, Fig 6 shows an atom group transferred from pyruvate to (S)-2-Acetolactate in the reaction R00226 of the KEGG RPAIR database[2,54]. In Fig 6, we can see that six connected atoms (The atoms circled with green dashed line in Fig 6) of pyruvate are transferred to (S)-2-Acetolactate in R00226, and the associated bonds connecting these atoms are conserved during the reaction. These six connected atoms and the bonds connecting the atoms construct an atom group transferred from pyruvate to (S)-2-Acetolactate in R00226.

From Fig 6, we can see that a compound is a graph composed of atoms (nodes) and bonds (edges), and the atom groups transferred between the compounds in the reaction are the sub-graph of the compounds. Furthermore, in Fig 6, the atom group transferred from substrate pyruvate to product (S)-2-Acetolactate in R00226 can be considered as the mapped sub-graph between substrate pyruvate and product (S)-2-Acetolactate. Combining the atom mapping information in the reactions of the KEGG RPAIR, we can obtain the atom groups transferred in the reactions through finding the mapped sub-graphs between the substrates and products.

Ullmann algorithm is a widely used algorithm for finding the mapped sub-graphs of two graphs [55]. Ullmann algorithm represents graphs by adjacency matrices, and computes the mapped sub-graphs between two graphs by matrix operations. For the given reaction *R* of the KEGG RPAIR, the substrate *S* of *R* can be represented as graph *G*_*A*_, the product *O* of *R* can be represented as graph *G*_*B*_. The nodes of *G*_*A*_ and *G*_*B*_ represent the atoms of compounds *S* and *O* respectively, and the edges of *G*_*A*_ and *G*_*B*_ represent the bonds of compounds *S* and *O*, respectively. We can combine the atom mapping information of the reaction *R* in the KEGG RPAIR and Ullmann algorithm to compute the mapped sub-graphs between *G*_*A*_ and *G*_*B*_ to obtain the atom groups transferred from substrate *S* to product *O* in reaction *R*.

The matrices *MA* and *MB* are the adjacency matrices of *G*_*A*_ and *G*_*B*_, respectively. The zero-one matrix *M* is the node mapping matrix of *G*_*A*_ and *G*_*B*_. *M*[*x*][*y*] = 1 when the node (atom) *x* of *G*_*A*_ is mapped to the node (atom) *y* of *G*_*B*_, and *M*[*x*][*y*] = 0 when *x* is not mapped to *y*. Here, the node (atom) mappings in *M* between *G*_*A*_ and *G*_*B*_ can be obtained from the atom mapping information between substrate *S* and product *O* in the reaction *R* of the KEGG RPAIR. We can compute the mapped sub-graphs between *G*_*A*_ and *G*_*B*_ based on the following formulas:

MC=M×(M×MB)’ (1)

$$edge\left(u,v\right)\in G_{A},edge\left(u’,v’\right)\in G_{B}:\;\left(MA\left[u\right]\left[v\right]=1\wedge MC\left[u\right]\left[v\right]=1\wedge M\left[u\right]\left[u’\right]=1\wedge M\left[v\right]\left[v’\right]=1\right)\Rightarrow \left(edge\left(u,v\right)\to edge\left(u’,v’\right)\right)$$ (2)

First, based on the adjacency matrix *MB* of graph *G*_*B*_ and the node(atom) mapping matrix *M* of graphs *G*_*A*_ and *G*_*B*_, we can use formula (1) to compute the potential mapped edges between *G*_*A*_ and *G*_*B*_, and conserve these potential mapped edges by zero-one matrix *MC*. In matrix *MC*, *MC*[*u*][*v*] = 1 when the edge(*u*,*v*) between nodes *u*, *v* of *G*_*A*_ is potentially mapped to *G*_*B*_, and *MC*[*u*][*v*] = 0 when the edge(*u*,*v*) between nodes *u*,*v* of *G*_*A*_ is not mapped to *G*_*B*_. Next, we can use formula (2) to check the consistency between the edges in *G*_*A*_ and the potential mapped edges in matrix *MC*. In formula (2), the edge (*u*,*v*) of *G*_*A*_ is correctly mapped to the edge(*u’*,*v’*) of *G*_*B*_ when *MA*[*u*][*v*] = 1 and *MC*[*u*][*v*] = 1 and *M*[*u*][*u’*] = 1 and *M*[*v*][*v’*] = 1, and this edge (*u*,*v*) is called the correct mapped edge for *G*_*A*_ and *G*_*B*_. We can use such correct mapped edges to construct the sub-graphs mapped from *G*_*A*_ to *G*_*B*_. And these mapped sub-graphs of graphs *G*_*A*_ and *G*_*B*_ are the atom groups transferred from substrate *S* to product *O* in the reaction *R*.

Thus, we can compute the atom groups transferred between substrate and product in each reaction by Ullmann algorithm based on the atom mapping information of the reactions in the KEGG RPAIR. In search of the linear atom group conserving pathways, BPFinder first finds top *k* shortest pathways between source and target compound in the metabolic network constructed by the reactions and compounds of the KEGG RPAIR. Then BPFinder utilizes the Ullmann algorithm to compute the atom groups transferred from source to target compound for each shortest pathway, and selects the linear pathways whose target compound contains the atom groups transferred from the source compound as the linear atom group conserving pathways. Note that, different from our previous linear pathfinding method AGPathfinder [31], BPFinder uses Ullmann algorithm to compute conserved atom groups whereas AGPathfinder computes conserved atom groups by finding connected components in mapped subgraphs between substrate and product without using Ullmann algorithm. And therefore BPFinder does not adopt AGPathfinder to identify linear atom group conserving pathways.

**Fig 7:**
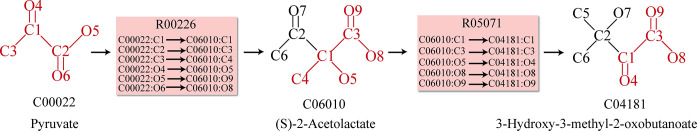
A linear atom group conserving pathway from pyruvate to 3-Hydroxy-3-methyl-2-oxobutanoate. This pathway is composed of reactions R00226 and R05071. The conserved atom group transferred from pyruvate to 3-Hydroxy-3-methyl-2-oxobutanoate is marked in red.

Fig 7 shows a linear atom group conserving pathways from pyruvate to 3-Hydroxy-3-methyl-2-oxobutanoate in the KEGG PATHWAY database. As we can see from Fig 7, an atom group with two carbon atoms and three oxygen atoms is transferred from pyruvate to 3-Hydroxy-3-methyl-2-oxobutanoate through reactions R00226 and R05071 in this linear pathway.

### Producing branched pathways by merging linear pathways

After obtaining multiple linear atom group conserving pathways from source to target compound, BPFinder selects branched compounds from the linear pathways based on the merging rule for branched compound, and then merges the linear pathways through the selected branched compounds to produce branched pathways. In the following, we will introduce the merging rule for branched compound in detail.

### Merging rule for branched compound

Based on the structure of the conserved atom groups transferred from the source to the branched compounds, we propose two merging rules for branched compounds: overlapping rule and non-overlapping rule.

Overlapping rule: In the linear pathways, the common branched compound that has overlapping conserved atom groups with other common branched compounds is called overlapping branched compound. The branched pathways can be produced by merging the linear pathways through the overlapping branched compounds.

**Fig 8:**
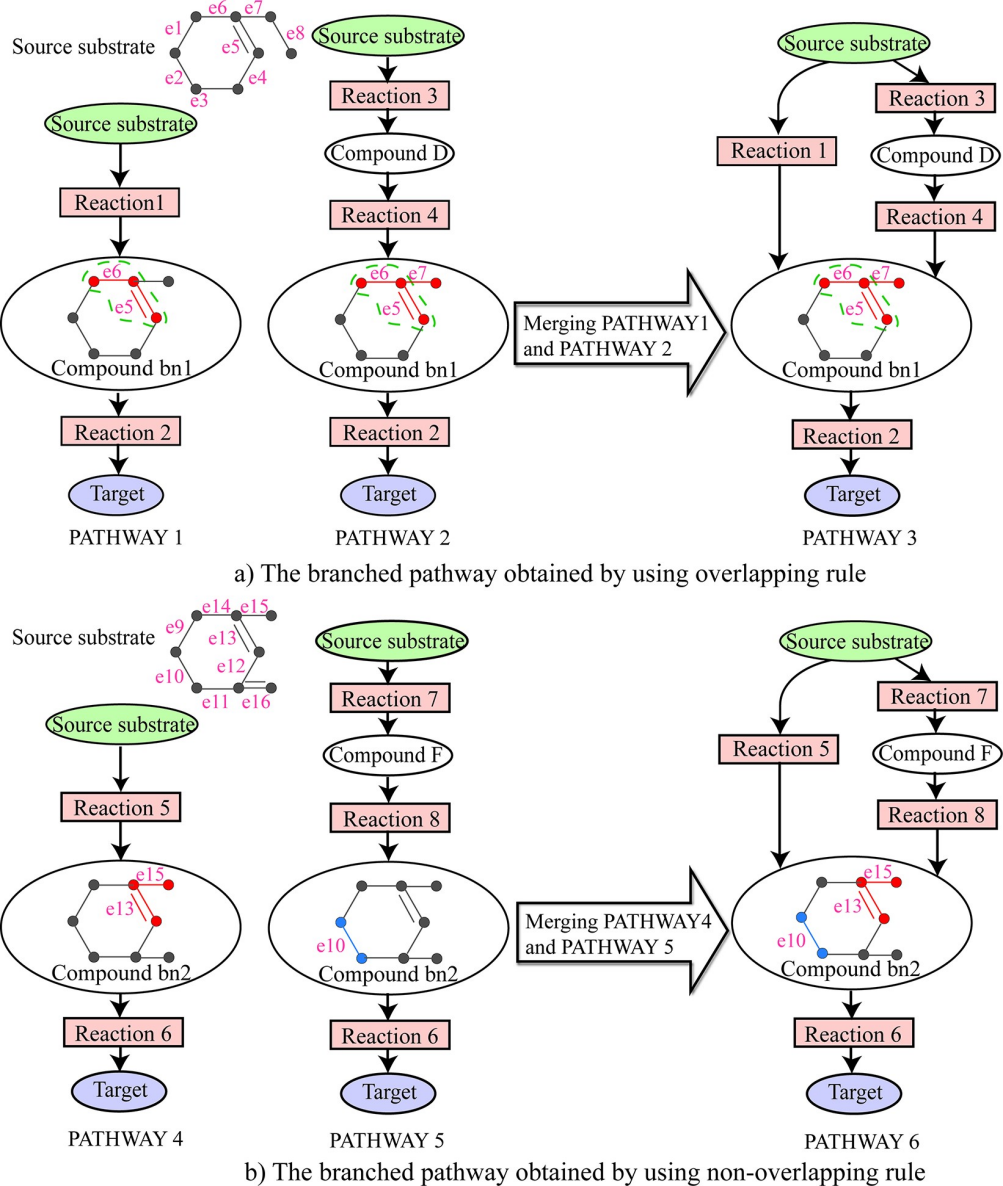
Two branched metabolic pathways obtained by using overlapping rule and non-overlapping rule respectively. **a)** The branched pathway obtained by using overlapping rule. **b)** The branched pathway obtained by using non-overlapping rule. In the compounds bn1 and bn2, a circle represents an atom and the edges connecting atoms represent bonds. The red and blue partitions in the compounds bn1 and bn2 are the conserved atom group transferred from the source compound. The partition circled with green dashed line in the compound bn1 is the overlapping part of the conserved atom group for pathways 1 and 2.

For example, Fig 8A shows a branched pathway produced by using the overlapping rule. In Fig 8A, we can see that the conserved atom groups in the common branched compound bn1 of the pathways 1 and 2 overlap with each other in the bonds e5 and e6. Following the overlapping rule, the branched pathway 3 can be produced by merging pathways 1 and 2 through the overlapping branched compound bn1.

Non-overlapping rule: In the linear pathways, the common branched compound that does not have overlapping conserved atom groups with other common branched compounds is called non-overlapping branched compound. The branched pathways can be produced by merging the linear pathways through the non-overlapping branched compounds.

Fig 8B shows a branched pathway produced by using non-overlapping rule. As can be seen from Fig 8B, the conserved atom group in the compound bn2 of pathway 4 consists of bonds e13, e15, and the conserved atom group in the compound bn2 of pathway 5 consists of bond e10. Thus, the conserved atom groups in the common branched compound bn2 of the pathways 4 and 5 do not overlap with each other. Following the non-overlapping rule, the branched pathway 6 can be produced by merging pathways 4 and 5 through the non-overlapping branched compound bn2.

### Producing branched metabolic pathways

For a group of linear atom group conserving pathways from source to target compound, the following Algorithm 1 chooses the compounds contained in at least two linear pathways as candidate branched compounds. Then, combining the overlaps of the conserved atom groups in the candidate branched compounds and the merging rule determined before searching pathways, Algorithm 1 selects compounds from the candidate branched compounds and merges the linear pathways by the selected compounds to produce branched pathways. Algorithm 1 is shown in Table 5.

**Table 5:** Producing branched pathways.

**Algorithm 1** Produce branched pathways
**Input:** The linear pathway set *P*, containing the linear atom group conserving pathways from the given source to target compound; Merging rule *MR* for branched compound
**Output:** The branched metabolic pathway set *BP*
1: *BrN* ← The candidate branched compounds contained in at least two linear pathways in the pathway set *P* except for the source and target compound;
2: *C*←{};// *C* stores the mappings between overlapping branched compounds and the linear pathways that contain the overlapping branched compounds
3: *C1*←{};// *C1* stores the mappings between non-overlapping branched compounds and the linear pathways that contain the non-overlapping branched compounds
4: **for** each candidate branched compound *Bn* **in** *BrN* **do:**
5: *overlap_atom* ←{};// *overlap_atom* stores the overlapping atom groups in branched compounds
6: *non_atom* ←{};// *non_atom* stores the non-overlapping atom groups in branched compounds
7: *P_over* ←{};// *P_over* stores the linear pathways that contain overlapping branched compounds
8: *P_non* ←{}; // *P_ non* stores the linear pathways that contain non-overlapping branched compounds
9: **for** the *i*th linear pathway *P*(*i*) **in** *P* **do:**
10: *IAGT* ← the conserved atom groups in the branched compound *Bn* of the pathway *P*(*i*);
11: **if** *overlap_atom* is null and *P*(*i*) is not the last pathway in *P*:
12: *overlap_atom*← the overlaps of the conserved atom groups in the branched compound *Bn* of the pathways *P*(*i*) and *P*(*i+*1);
13: **if** the atom groups in *IAGT* do not overlap with the atom groups in *overlap_atom*:
14: Add the atom groups in *IAGT* to *non_atom*;
15: Add *P*(*i*) to *P_non*;
16: **else**
17: *overlap_atom*← the overlaps of the conserved atom groups of *IAGT* and *overlap_atom*;
18: Add *P*(*i*) to *P_over*;
19: **if** *overlap_atom* is not null and the number of pathways in *P_over* is greater than 1:
20: Add (*Bn*, *P_over*) to *C*;
21: **if** *non_atom* is not null and the number of pathways in *P_non* is greater than 1:
22: Add (*Bn*, *P_non*) to *C1*;
23: **if** *MR* = ‘overlapping rule’:
24: *W* ← the combinations of all branched compounds in *C*;
25: **for** each branched compound combination *w*_*i*_ in *W*:
26: Extract the overlapping branched compounds from *w*_*i*_, and merge the linear pathways in *C* through the extracted compounds to produce branched pathways, and add the produced pathways to *BP*;
27: **else if** *MR* = ‘non-overlapping rule’:
28: *W*’ ← the combinations of all branched compounds in *C1*;
29: **for** each branched compound combination *w*_*i*_’ **in** *W*’:
30: Extract the non-overlapping branched compounds from *w*_*i*_’, and merge the linear pathways in *C1* through the extracted compounds to produce branched pathways, and add the produced pathways to *BP*;
31: **return** BP;

First, except for the source and target compounds, Algorithm 1 selects the compounds contained in at least two pathways in the linear pathway set *P* as the candidate branched compounds, and adds the candidate branched compounds to a branched compound set *BrN* (line 1). For each candidate branched compound *Bn* in *BrN*, Algorithm 1 checks the overlaps of the conserved atom groups in the branched compound *Bn* of the pathways *P*(*i*) and *P*(*i+*1) in *P* (lines 2–12). If there is no overlap in the conserved atom groups of the branched compound *Bn* of *P*(*i*) and *P*(*i+*1), Algorithm 1 adds *P*(*i*) to *P_non* and stores the conserved atom groups in *Bn* of *P*(*i*) to *non-atom* (lines 13–15). Else if there are overlaps in the conserved atom groups of the branched compound *Bn* of *P*(*i*) and *P*(*i+*1), Algorithm 1 adds *P*(*i*) to *P_over* and stores the conserved atom groups in *Bn* of *P*(*i*) to *overlap_atom* (lines 16–18).

After checking the overlaps of the conserved atom groups in the branched compound *Bn* of the pathways in *P*, if *overlap_atom* is not null, which indicates *Bn* is an overlapping branched compound, Algorithm 1 adds the mapping (*Bn*, *P_over*) between *Bn* and *P_over* to *C* (lines 19–20). If *non_atom* is not null, which indicates *Bn* is a non-overlapping branched compound, Algorithm 1 adds the mapping (*Bn*, *P_non*) between *Bn* and *P_non* to *C1* (lines 21–22).

Finally, when the merging rule determined by the user is ‘overlapping rule’, Algorithm 1 enumerates the combinations of all branched compounds in *C*, and extracts the overlapping branched compounds from the enumerated combinations of branched compound. And then Algorithm 1 merges the linear pathways in *C* through the extracted compounds to produce branched pathways, and add the produced pathways to *BP* (lines 23–26). Similarly, when the merging rule determined by the user is ‘non-overlapping rule’, Algorithm 1 extracts the non-overlapping branched compounds from *C1* in the similar way, and merges the linear pathways in *C1* through the extracted compounds to produce branched pathways (lines 27–30).

### Sorting branched metabolic pathways

After generating multiple branched metabolic pathways by Algorithm 1, in order to pick out biochemically feasible branched pathways of interest, we intend to sort the found branched pathways for the user. We combine the biochemical information of the linear pathways in branched pathways and branched compounds to score the resulting branched pathways, and the pathways with higher scores are ranked ahead.

It is known that the compound similarity, reaction thermodynamics and the number of conserved atom groups transferred from source to target compounds have directly impact on the biochemical relevance of metabolic pathways [24,25,31,34]. Thus, we utilize these biochemical factors to evaluate the biochemical relevance of linear metabolic pathways.

Following the literature [31], we also use SMSD tool [56] to compute the similarity values between the input and output compounds in the reactions of the pathway. Gibbs free energy is usually used to evaluate the thermodynamic feasibility of the reactions in the metabolic pathway [57]. In this work, the Gibbs free energy change of the reaction in KEGG RPAIR database is denoted as Δ*G_r_*, and the values of Δ*G_r_* are retrieved from the literature [57]. For the linear atom group conserving pathway, the number of conserved atom groups transferred from source to target compound would be useful information for pathway sorting since this information may assist in ranking the pathways transferring conserved atom groups, and the pathways with more conserved atom groups could be ranked ahead in searching results depending on the users’ interest in conserved atom groups in the pathway. For the given linear pathway *p*_*a*_, we can compute the linear pathway score Score(*p*_*a*_) by the following formula:

$$\mathrm{Score}\left(p_{a}\right)=\alpha_{s}\times \overset{l_{c}}{\underset{i=0}{\sum }}sim\left(v_{i},v_{j}\right)-\alpha_{sf}\times \overset{l_{c}}{\underset{i=0}{\sum }}\frac{3200+fe\left(r_{i}\right)}{10000}+\alpha_{t}\times \frac{t}{e}$$ (3)

In formula (3), *v*_*i*_ and *v*_*j*_ are the compounds connected by the reaction in the pathway *p*_*a*_, *sim*(*v*_*i*_,*v*_*j*_) is the compound similarity of *v*_*i*_ and *v*_*j*_, and the value of *sim*(*v*_*i*_,*v*_*j*_) is between 0 and 1. *l*_*c*_ is the number of reactions in the pathway *p*_*a*_. *r*_*i*_ is a reaction in the pathway *p*_*a*_, *fe*(*r*_*i*_) is value of the Δ*G_r_* of reaction *r*_*i*_. Note that the constants 10000 and 3200 are utilized to normalize the value of *fe*(*r*_*i*_). In formula (3), the value of *sim*(*v*_*i*_,*v*_*j*_) is between 0 and 1, and the value of *fe*(*r*_*i*_) retrieved from the literature [57] is between -2233.7 and 10194.7. That is, the values of *fe*(*r*_*i*_) and *sim*(*v*_*i*_,*v*_*j*_) are very different, and the normalization (3200+ *fe*(*r*_*i*_))/10000 is to adjust the value of *fe*(*r*_*i*_) to the range [0.097, 1.339] and brings the values of *fe*(*r*_*i*_) and *sim*(*v*_*i*_,*v*_*j*_) into alignment [31]. *t* is the number of the minimal atom groups transferred from source to target compound in the pathway *p*_*a*_. A minimal atom group is composed of a covalent bond with two atoms. *e* is the number of the covalent bonds in the source compound. $\frac{t}{e}$ is to adjust the value of *t* to the range [0, 1]. *α*_*s*_, *α*_*sf*_ and *α*_*t*_ are weight parameters, which are used to adjust the relative weights of compound similarity, Gibbs free energy of reaction and conserved atom groups in formula (3) respectively. The values of *α*_*s*_, *α*_*sf*_ and *α*_*t*_ are between 0 and 1, and can be adjusted by the user.

We can obtain the score of the linear pathway that consists of branched pathway by formula (3). Combining formula (3) and the information of the branched compounds, for the given branched pathway *p*_*b*_, we can compute the branched pathway score Score_Branch(*p*_*b*_) by the following formula:

$$\mathrm{Score}\_\mathrm{Branch}\left(p_{b}\right)=\alpha_{p}\times n_{b}+\left(1-\alpha_{p}\right)\times \frac{\sum_{i=0}^{l_{b}}\mathrm{Score}\left(p_{i}\right)}{l_{b}}$$ (4)

In formula (4), *n*_*b*_ is the number of the branched compounds in the branched pathway *p*_*b*_. *l*_*b*_ is the number of linear pathways in *p*_*b*_. *p*_*i*_ is the *i*th linear pathway that consists of *p*_*b*_. Score(*p*_*i*_) is the score of *p*_*i*_ computed by formula (3). *α*_*p*_ is weight parameter, which is used to adjust the relative weights of the branched compounds and the score of linear pathways in formula (4). The value of *α*_*p*_ is between 0 and 1, and can be adjusted by the user. We can compute the scores of the resulting branched pathways by formula (4), and sort the branched pathways by the computed scores in descending order.

## Supporting information

S1 Text The 30 known core metabolic pathways. (DOCX)

S2 Text Running parameters. (DOCX)

S3 Text The 20 known organism-specific pathways. (DOCX)

S4 Text Organism list for organism-specific pathways. (XLSX)
