# Supplementary material for: Finding branched pathways in metabolic network via atom group tracking
Source: PLoS Comput Biol. 2021 Feb 2;17(2):e1008676. doi: 10.1371/journal.pcbi.1008676 (PMC7880430; doi:10.1371/journal.pcbi.1008676)
Supplement: S1 Text — (DOCX) [file pcbi.1008676.s001.docx]

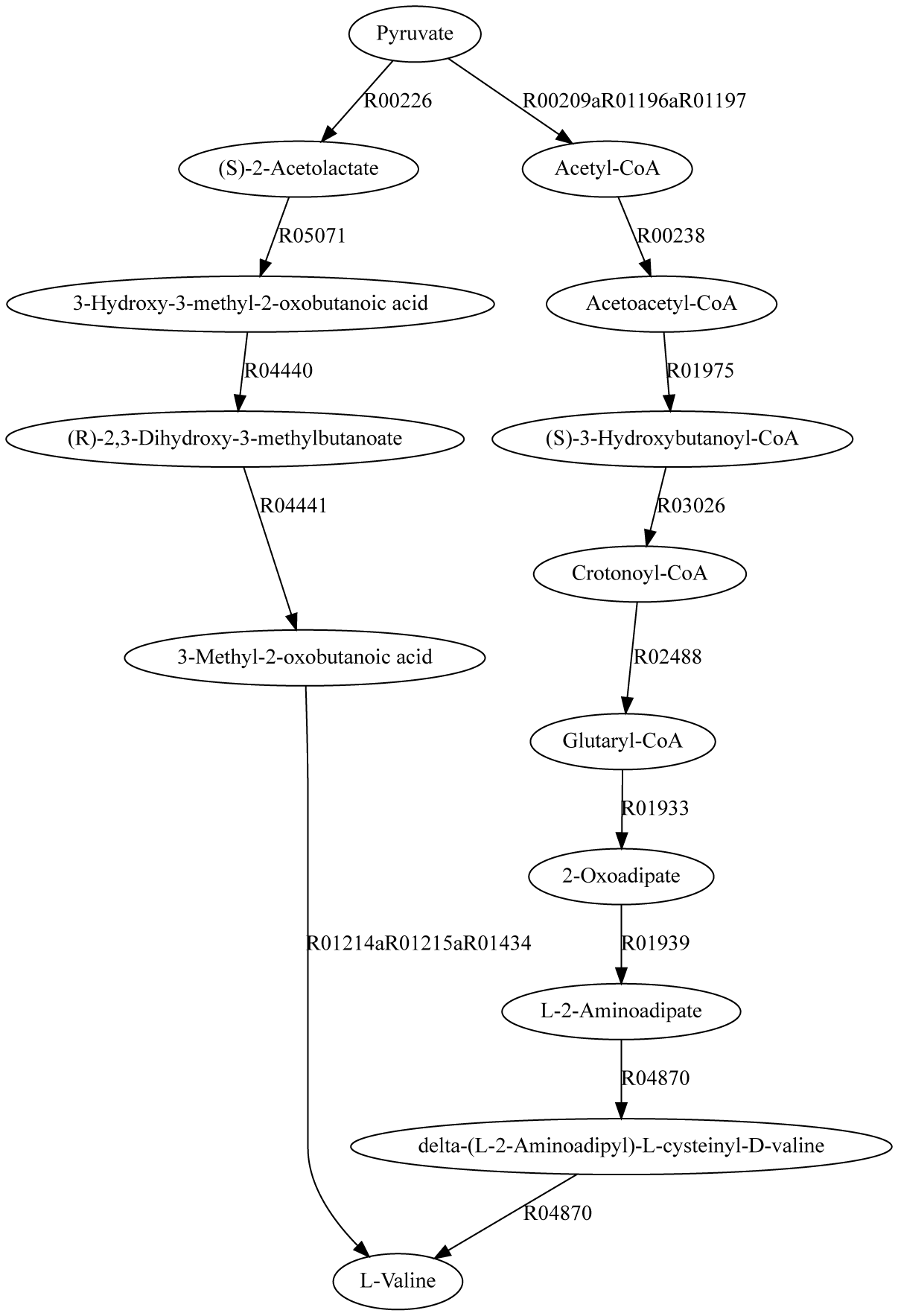


**Fig 1(S). The known pathway of Pyruvate to L-Valine from KEGG rn01130.**


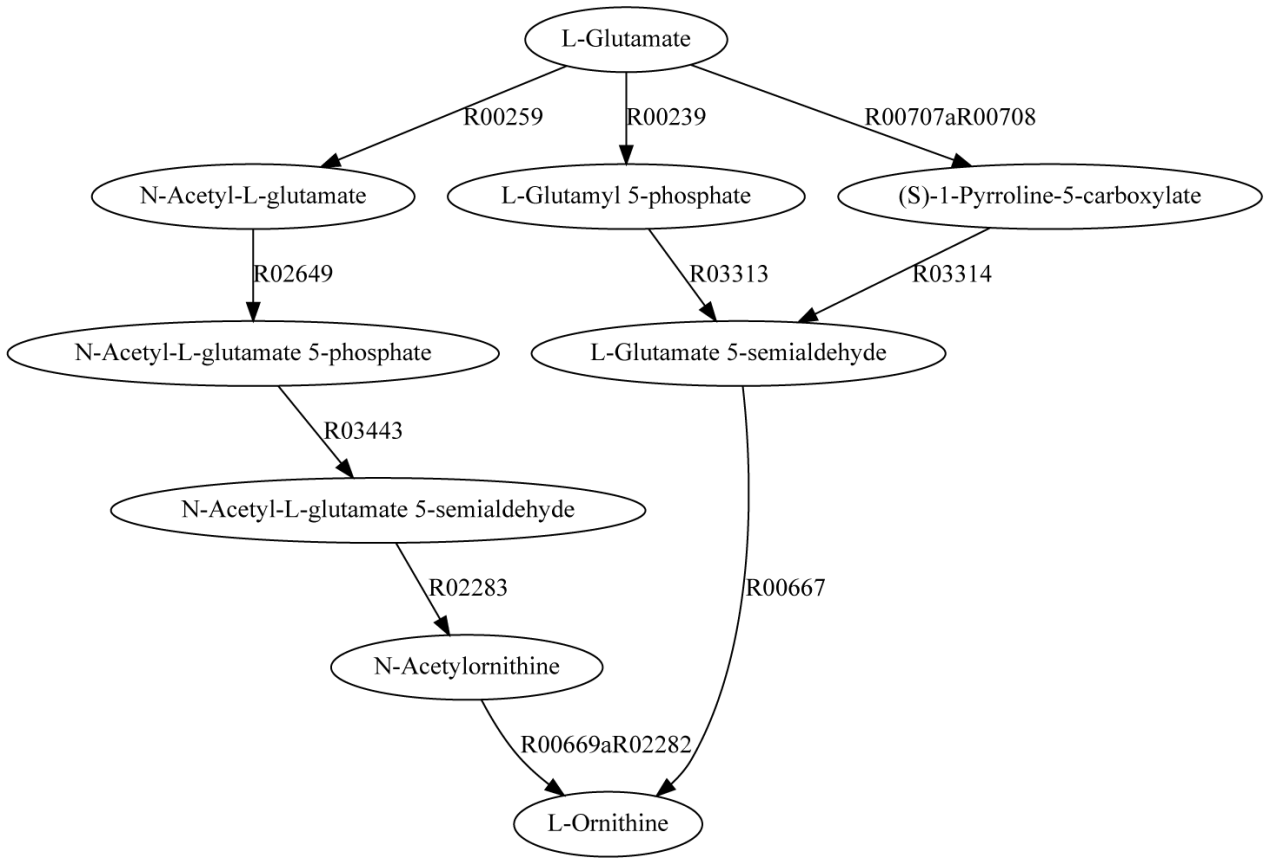


**Fig 2(S). The known pathway of L-Glutamate to L-Ornithine from KEGG rn01100.**


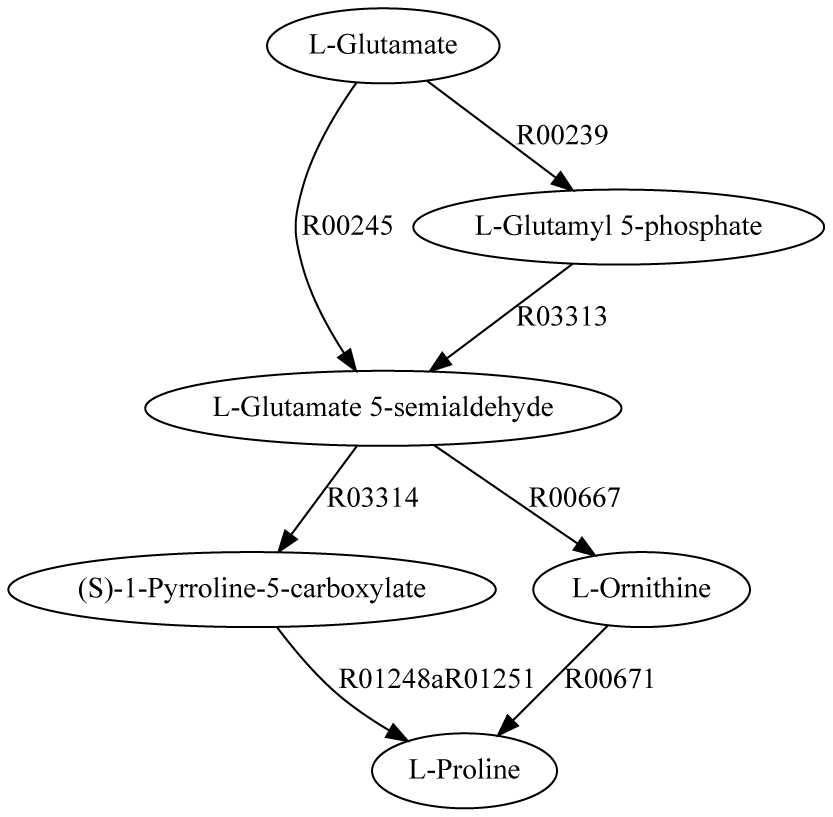


**Fig 3(S). The known pathway of L-Glutamete to L-Proline from KEGG rn00330.**

**
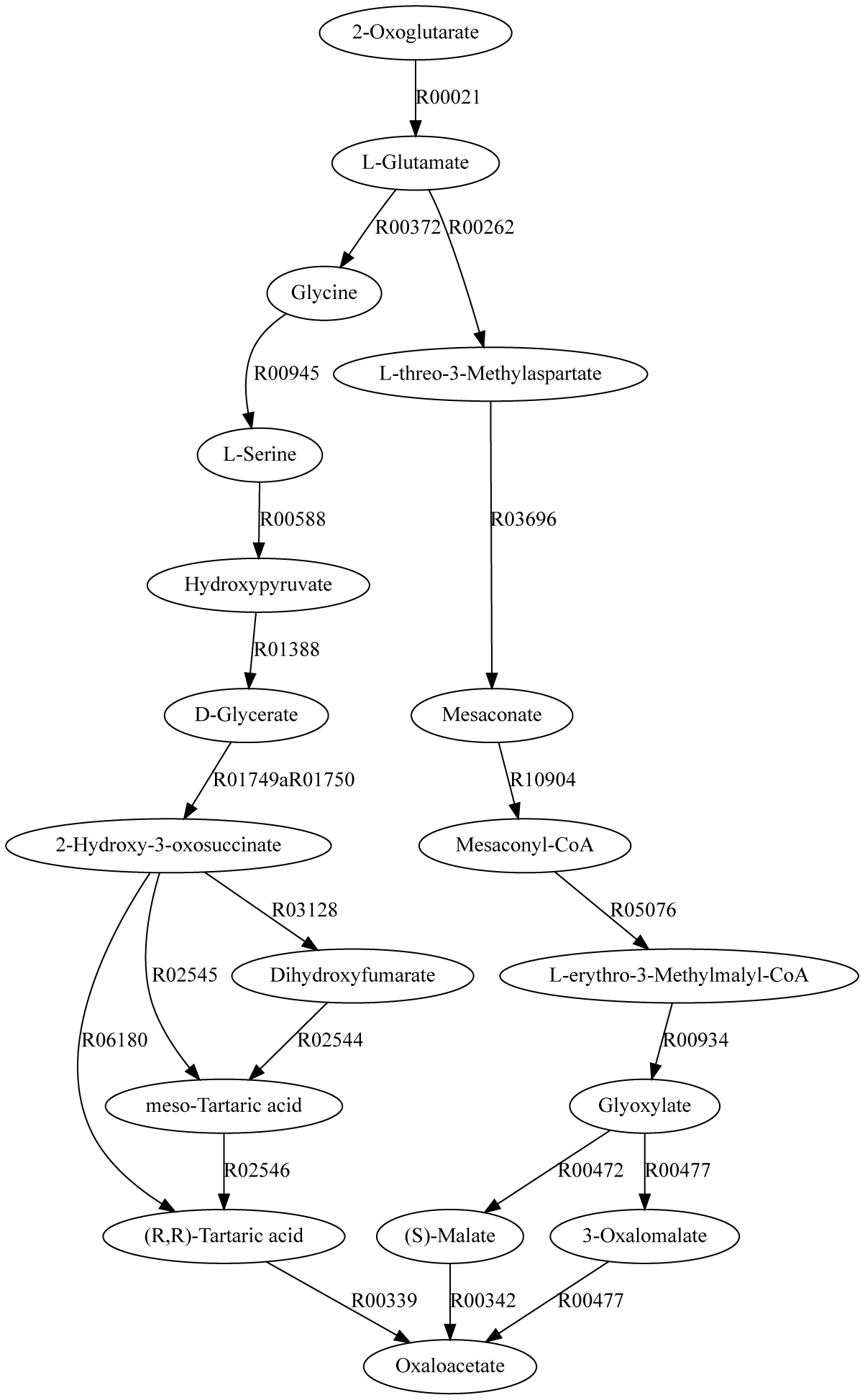
**

**Fig 4(S). The known pathway of 2-Oxoglutarate to Oxaloacetate from KEGG rn00630.**

**
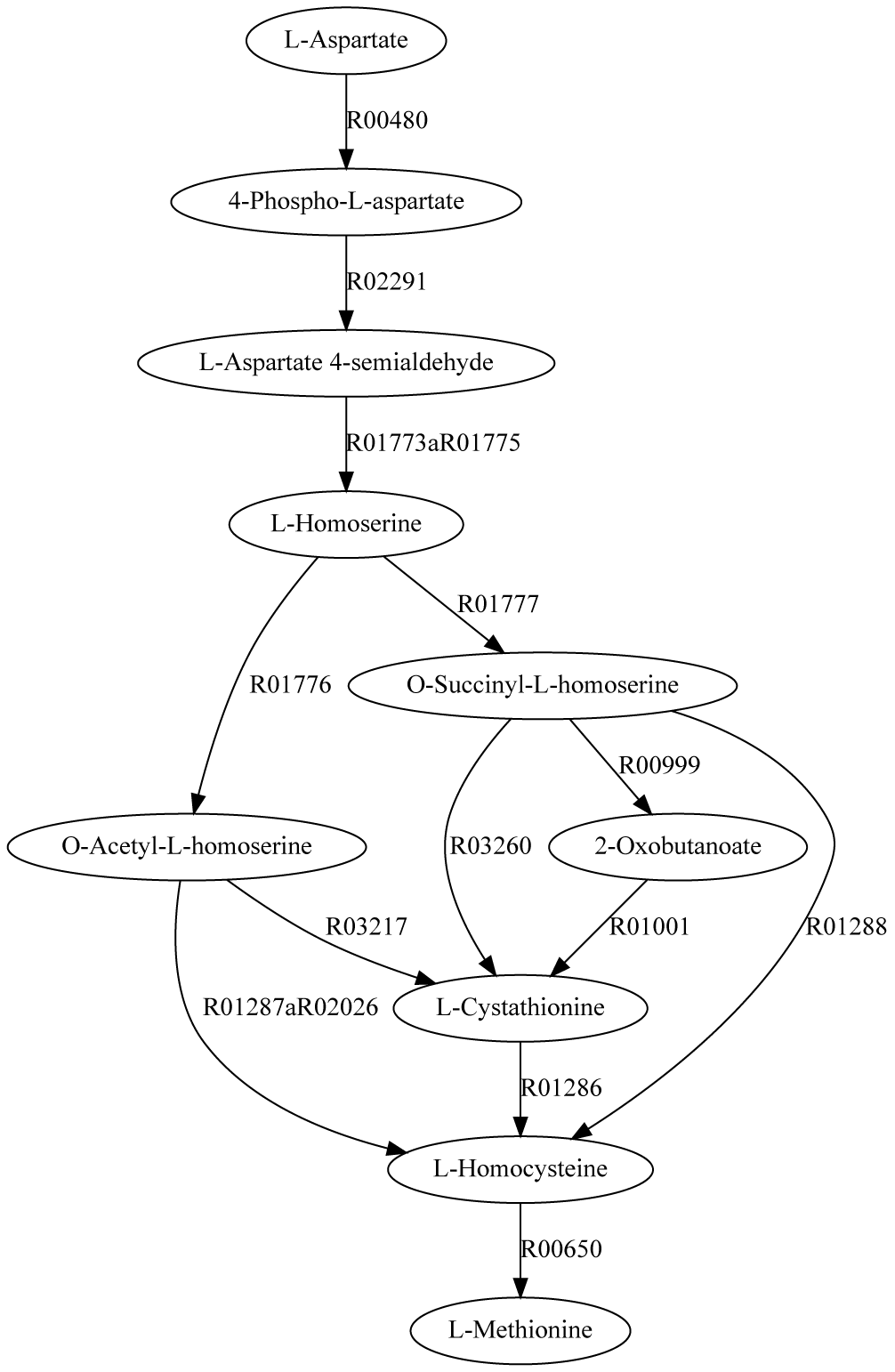
**

**Fig 5(S). The known pathway of L-Aspartate to L-Methionine from KEGG rn00270**

**
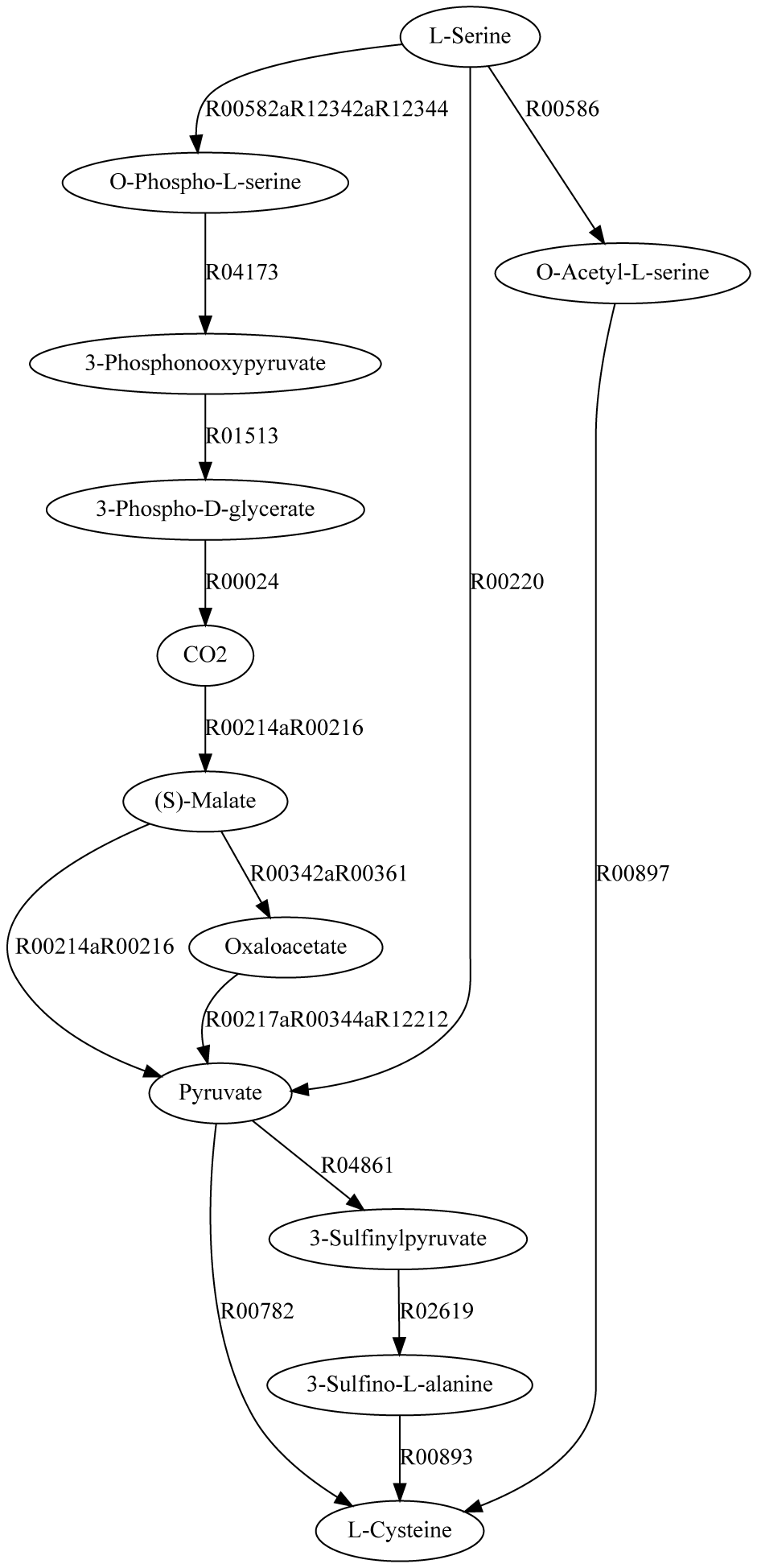
**

**Fig 6(S). The known pathway of L-Serine to L-Cysteine from KEGG rn01100.**

**
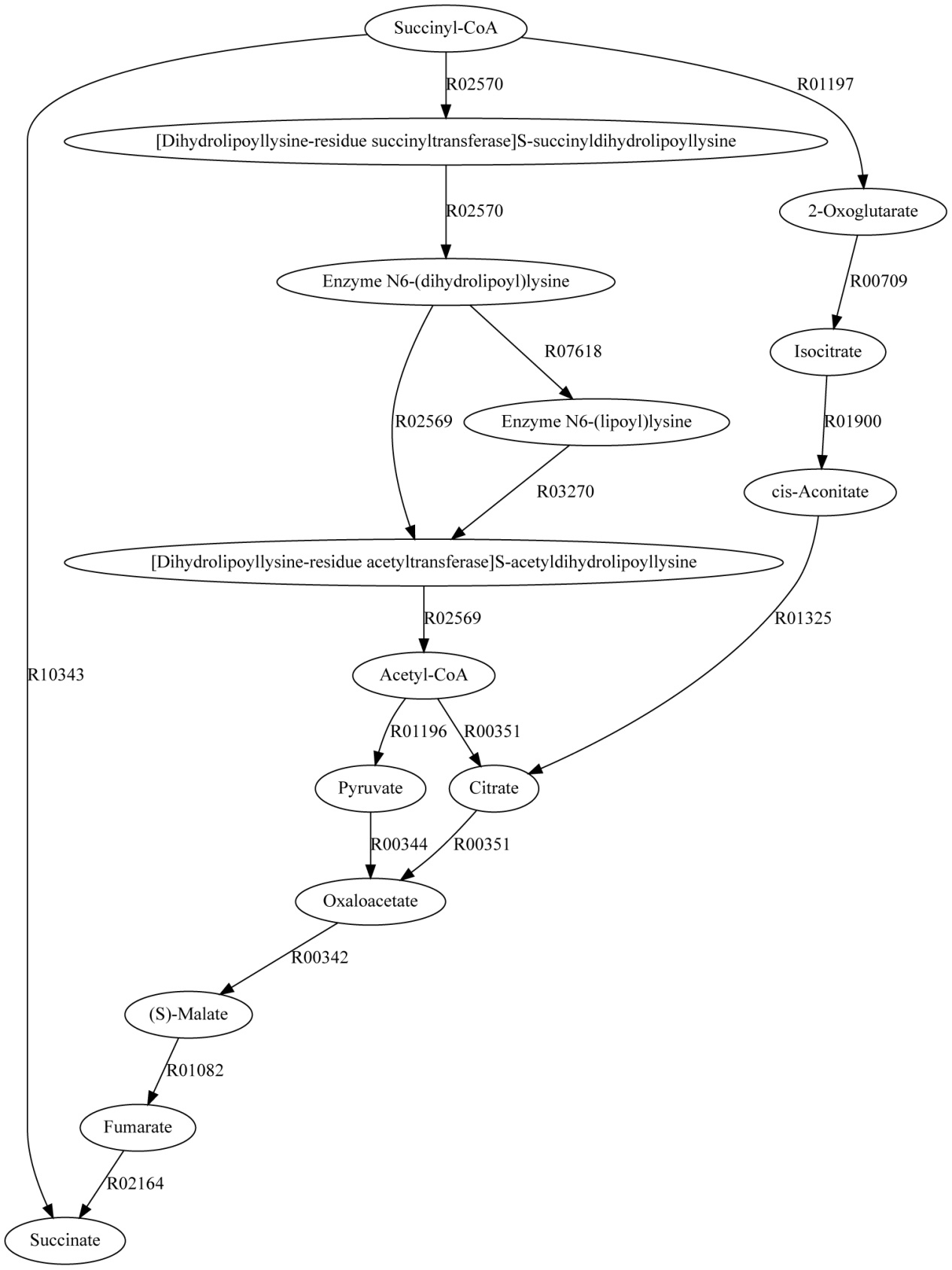
**

**Fig 7(S). The known pathway of Succinyl-CoA to Succinate from KEGG rn00020.**

**
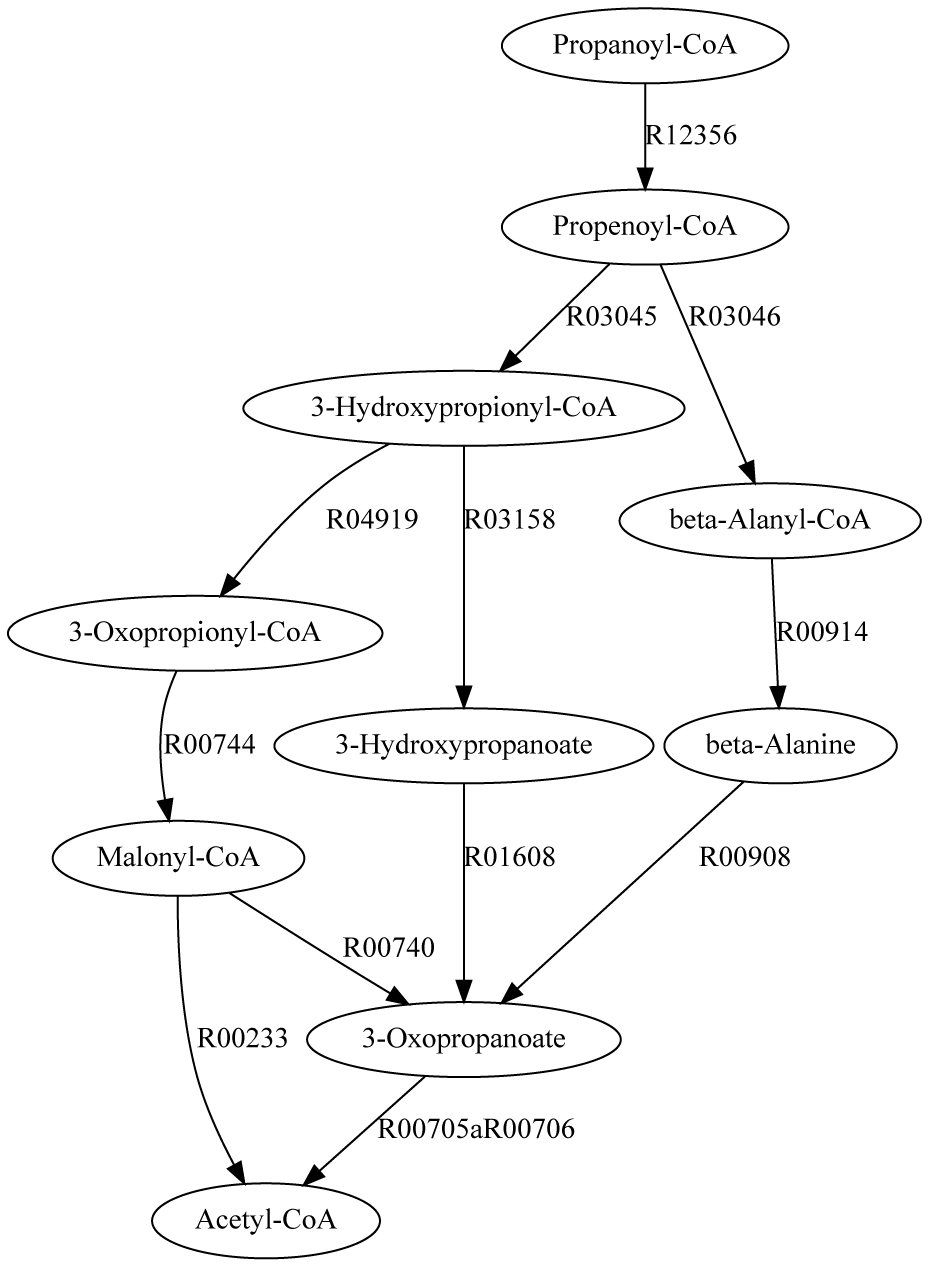
**

**Fig 8(S). The known pathway of Propanoyl-CoA to Acetyl-CoA from KEGG rn00640.**

**
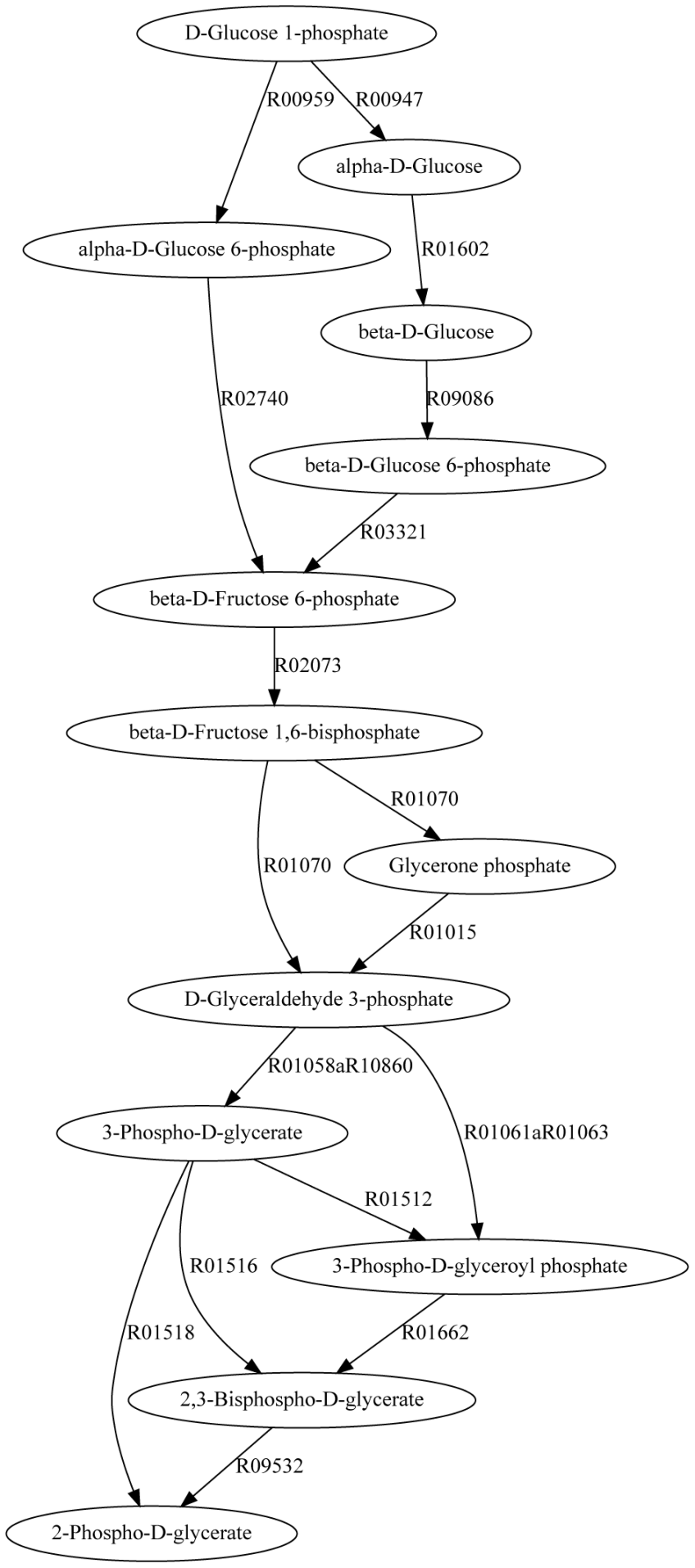
**

**Fig 9(S). The known pathway of D-Glucose 1-phsophate to 2-Phospho-D-glycerate from KEGG rn00010.**

**
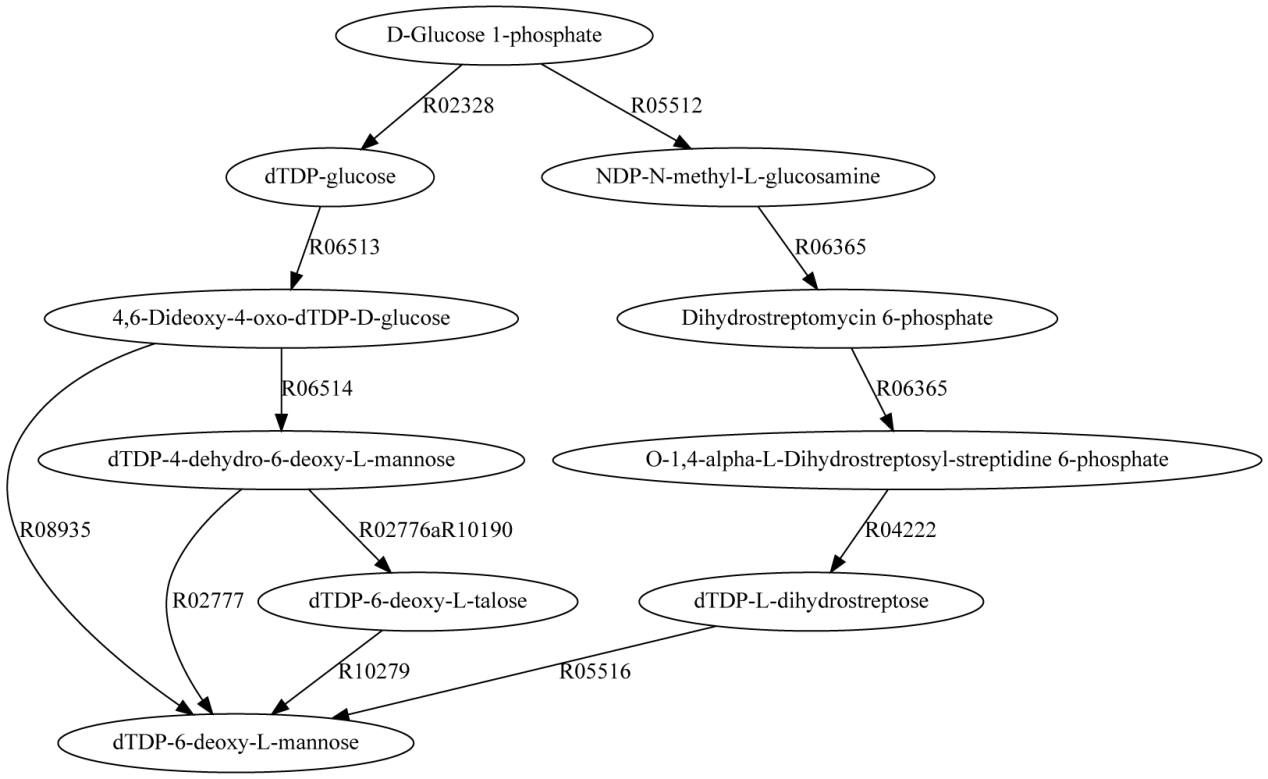
**

**Fig 10(S). The known pathway of D-Glucose 1-phsophate to dTDP-6-deoxy-L-mannose from KEGG rn01130.**

**
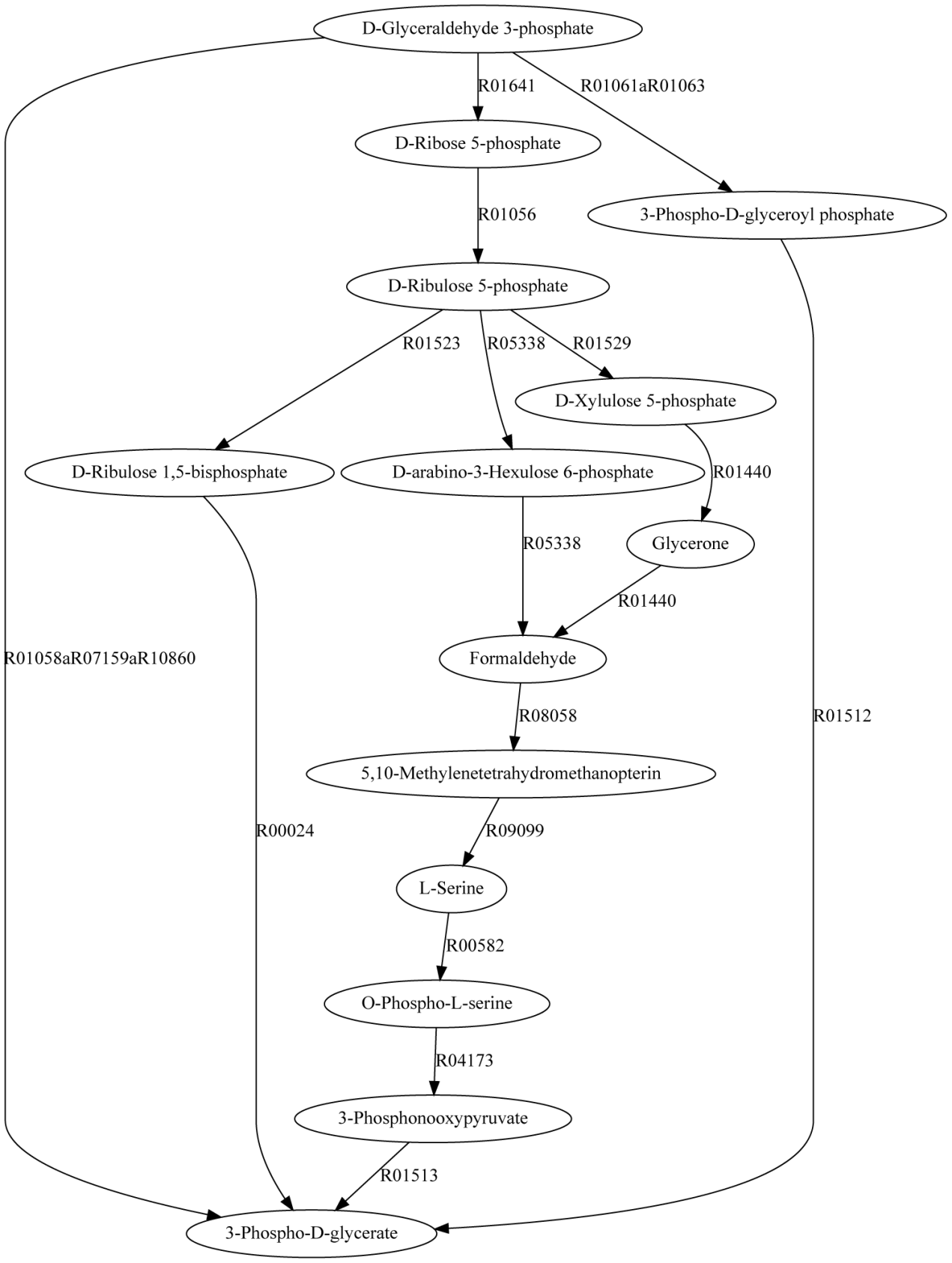
**

**Fig 11(S). The known pathway of D-Glyceraldehyde 3-phosphate to 3-Phospho-D-glycerate from KEGG rn01120.**

**
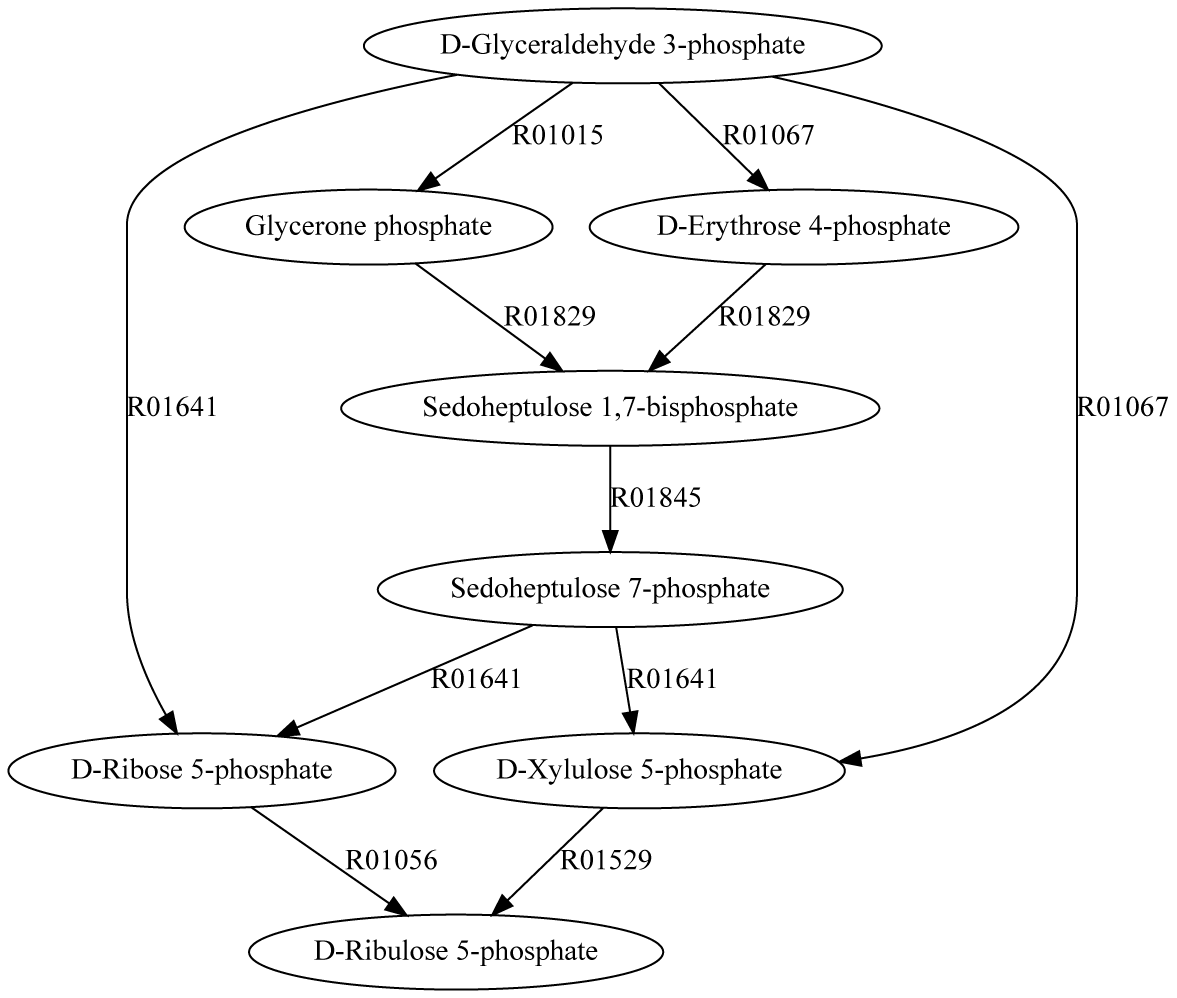
**

**Fig 12(S). The known pathway of D-Glyceraldehyde 3-phosphate to D-Ribulose 5-phosphate from KEGG rn00710.**

**
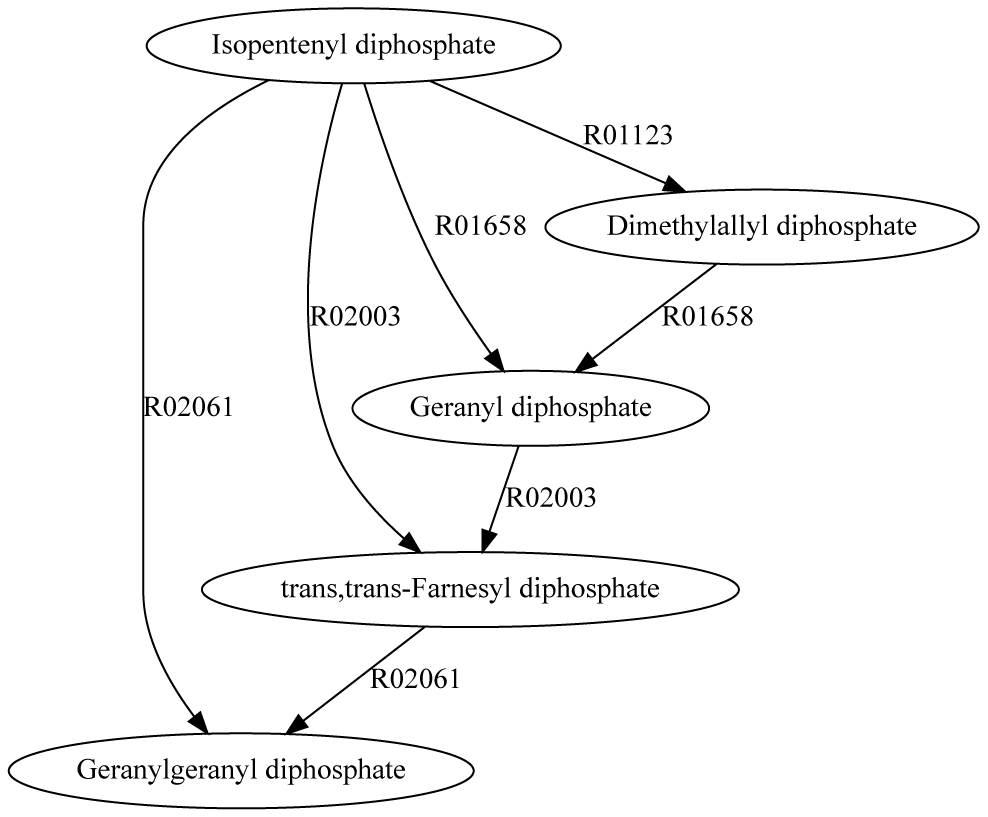
**

**Fig 13(S). The known pathway of Isopentenyl diphosphate to Geranylgeranyl diphosphate from KEGG rn00900.**

**
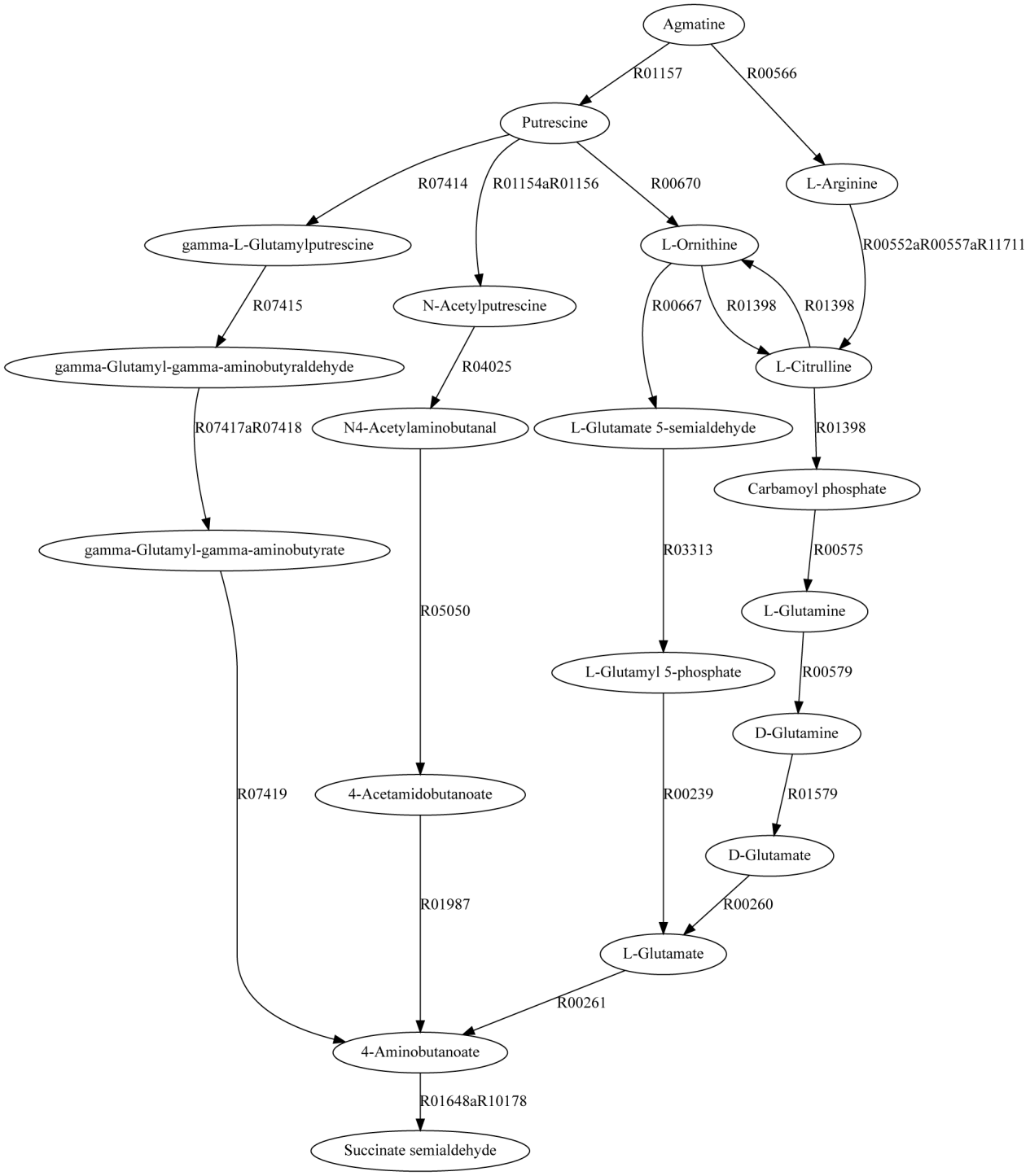
**

**Fig 14(S). The known pathway of Agmatine to Succinate semialdehyde from KEGG rn01100.**

**
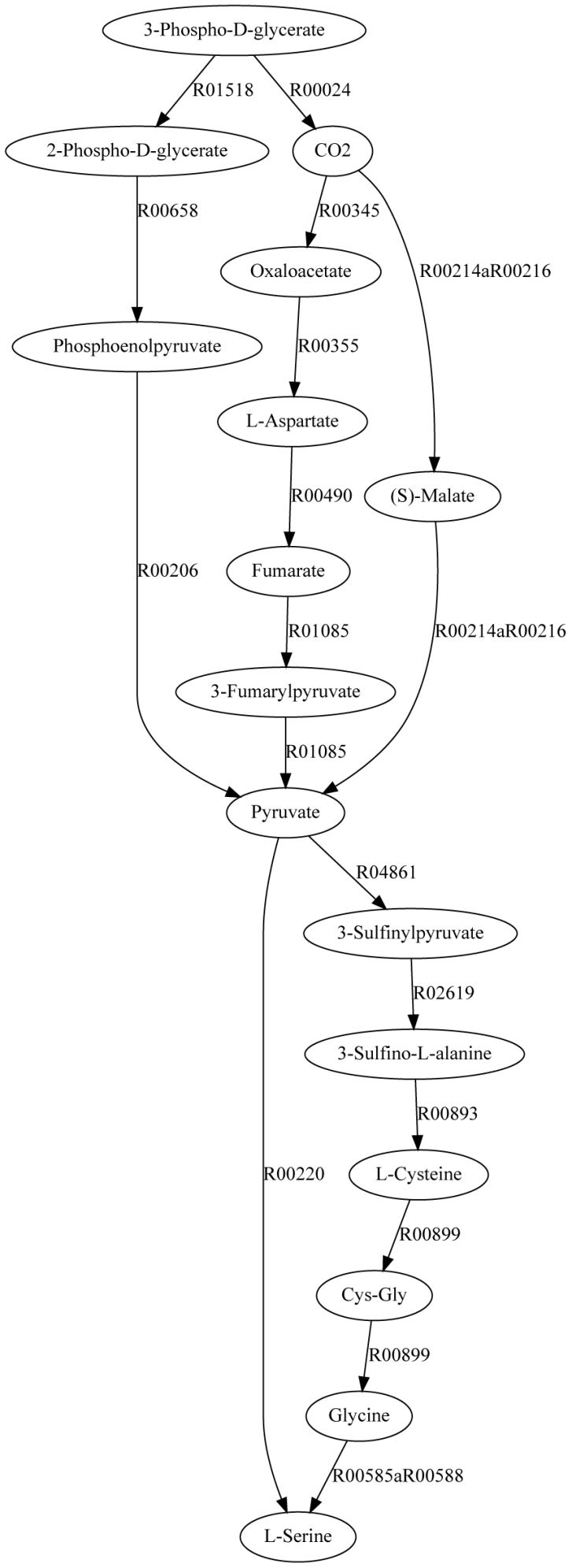
**

**Fig 15(S). The known pathway of 3-Phospho-D-glycerate to L-Serine from KEGG rn01100.**

**
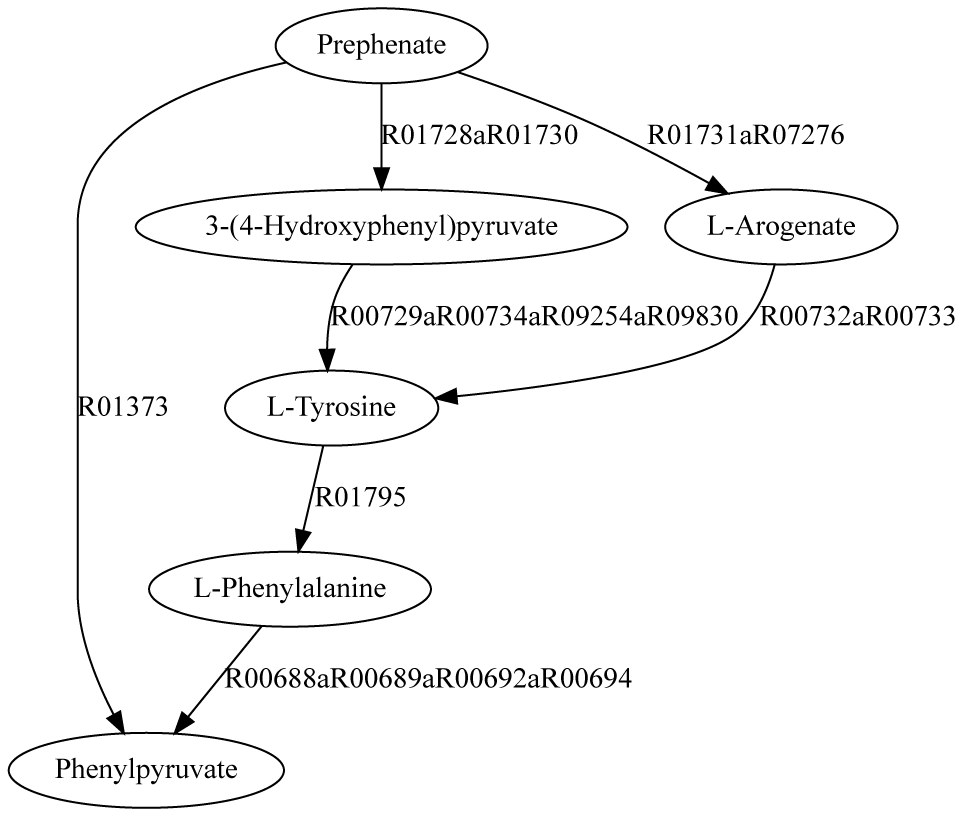
**

**Fig 16(S). The known pathway of Prephenate to Phenylpyruvate from KEGG rn01100.**

**
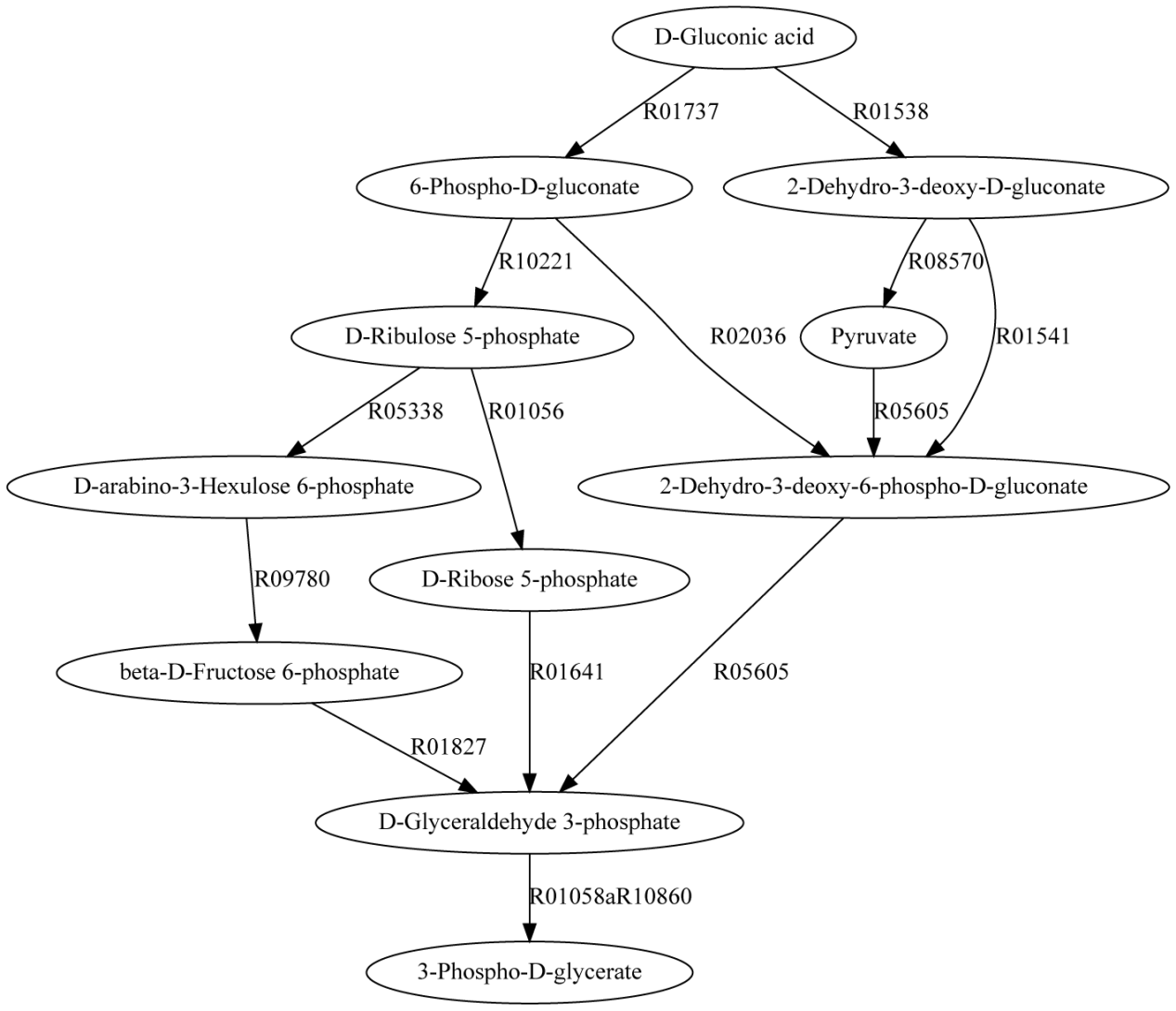
**

**Fig 17(S). The known pathway of D-Gluconic acid to 3-Phospho-D-glycerate from KEGG rn00030.**

**
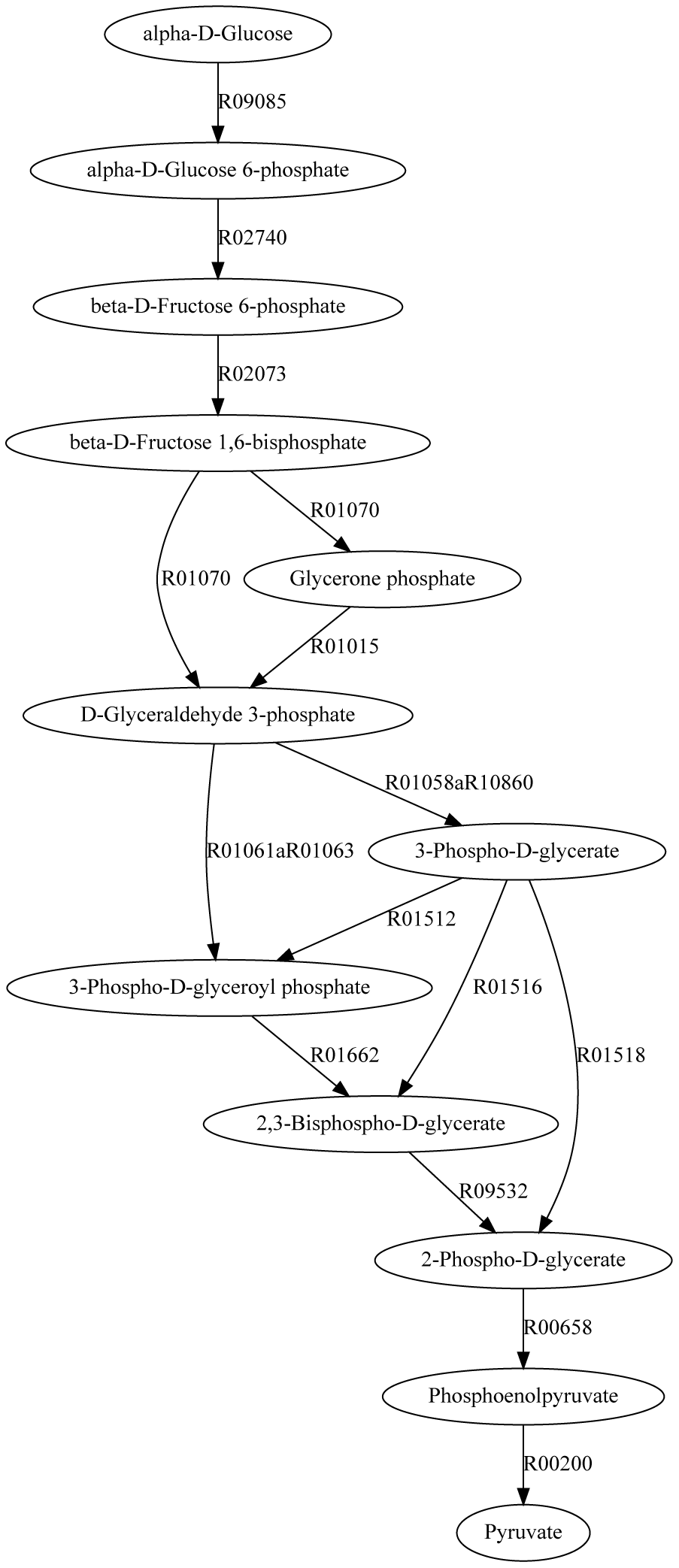
**

**Fig 18(S). The known pathway of alpha-D-Glucose to Pyruvate from KEGG rn00010.**

**
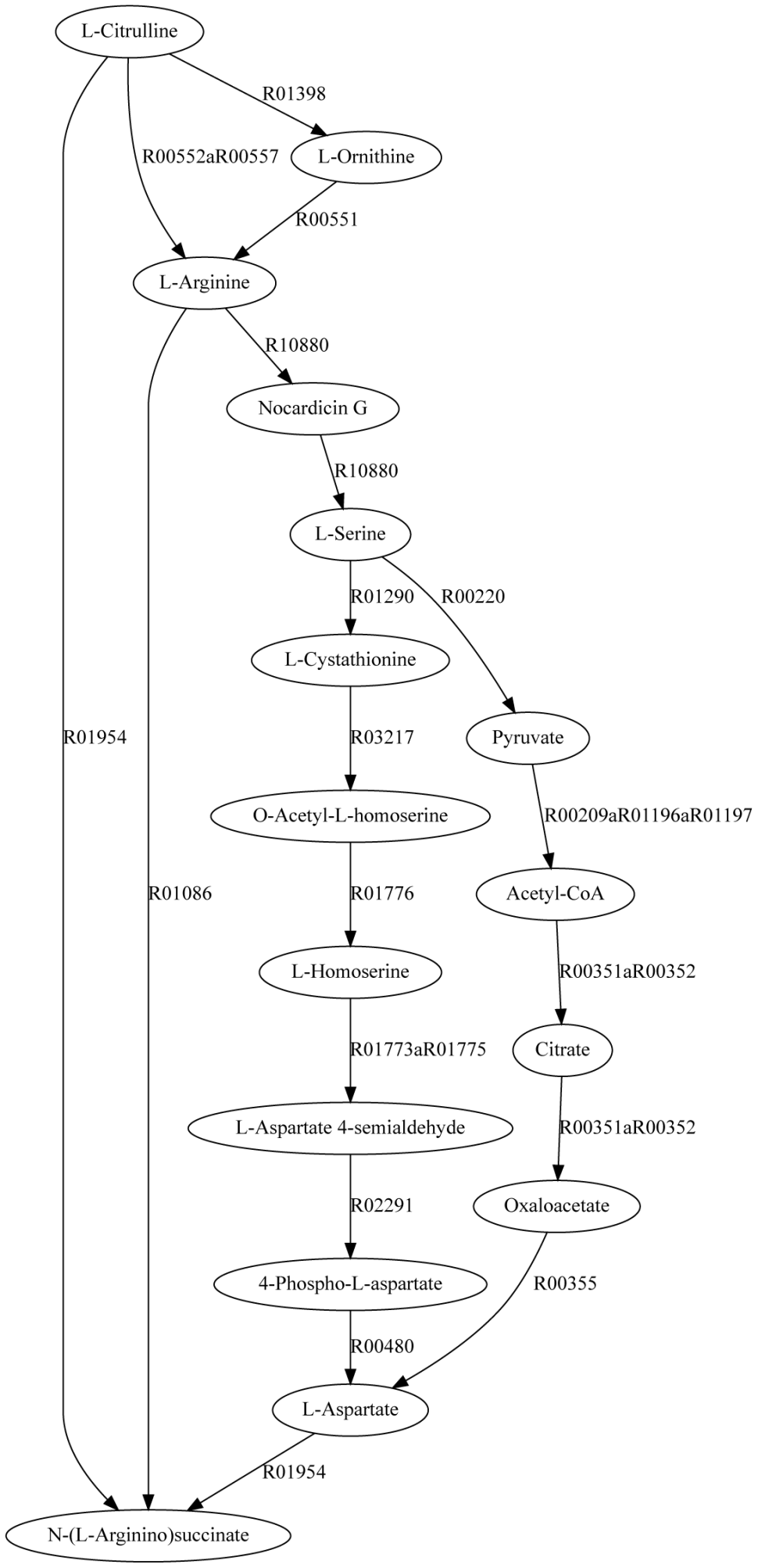
**

**Fig 19(S). The known pathway of L-Citrulline to N-(L-Arginino) succinate from KEGG rn01130.**

**
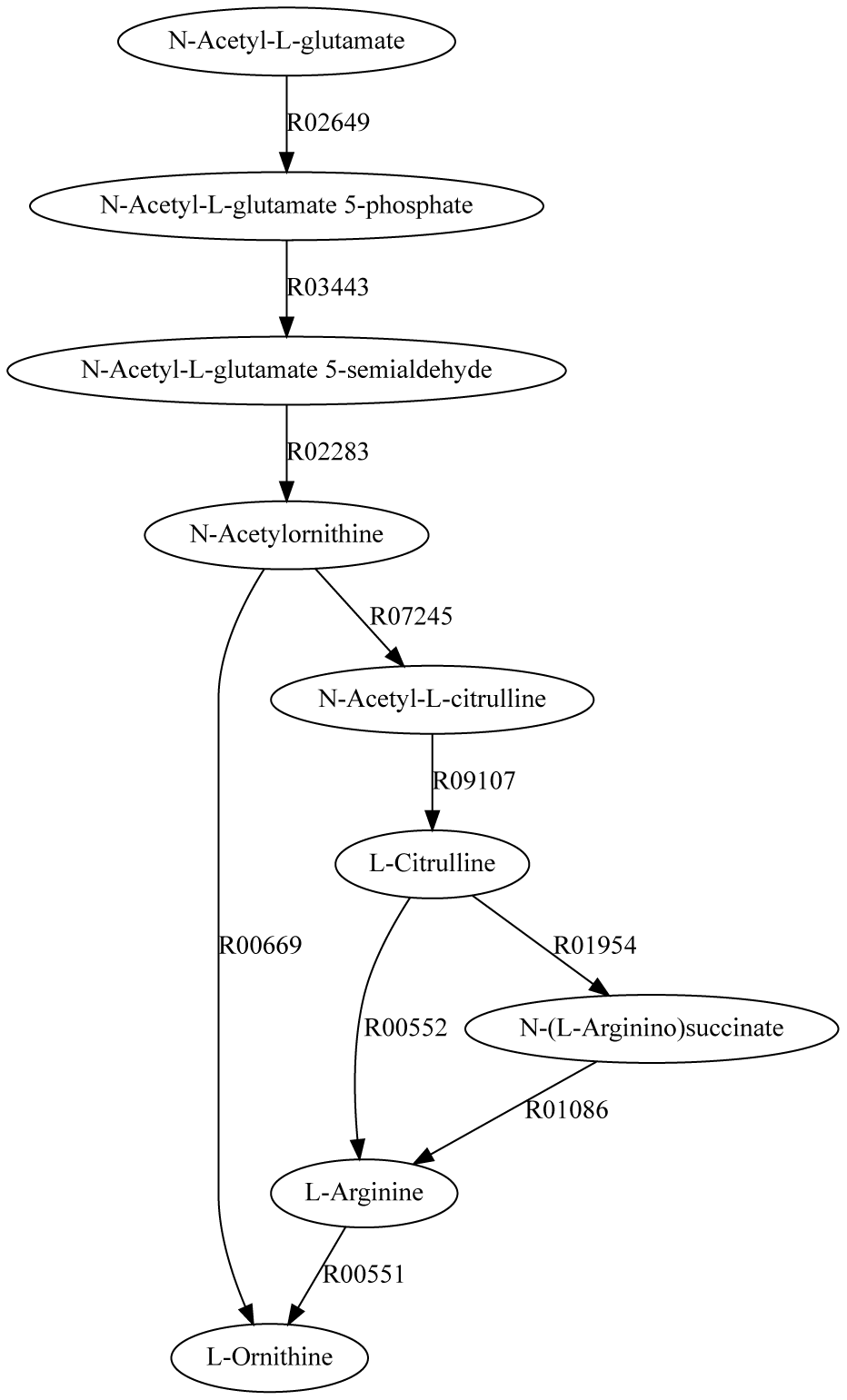
**

**Fig 20(S). The known pathway of N-Acetyl-L-glutamate to L-Ornithine from KEGG rn00220.**

**
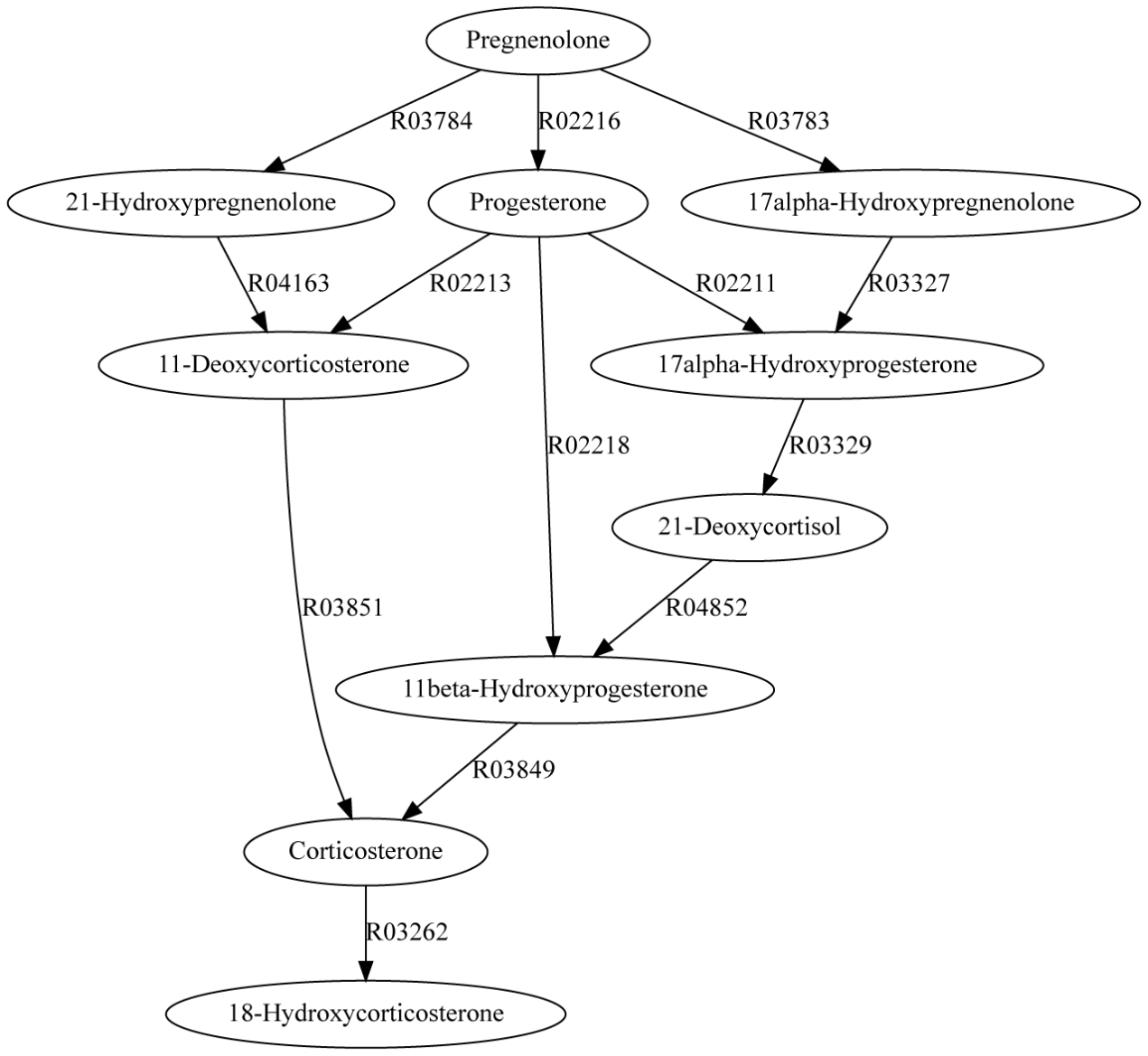
**

**Fig 21(S). The known pathway of Pregnenolone to 18-Hydroxycorticosterone from KEGG rn00140.**

**
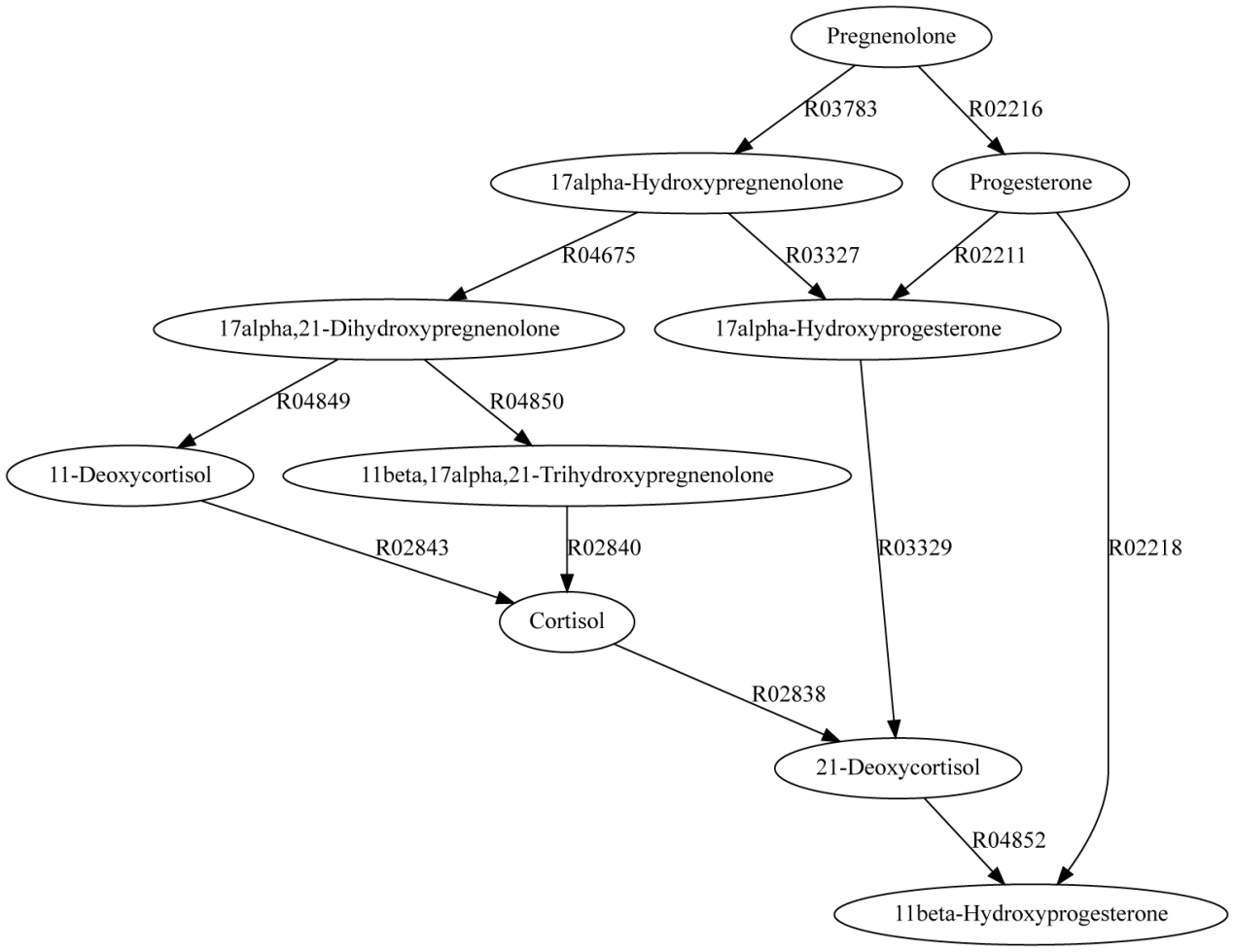
**

**Fig 22(S). The known pathway of Pregnenolone to 11 beta-Hydroxyprogesterone from KEGG rn00140.**

**
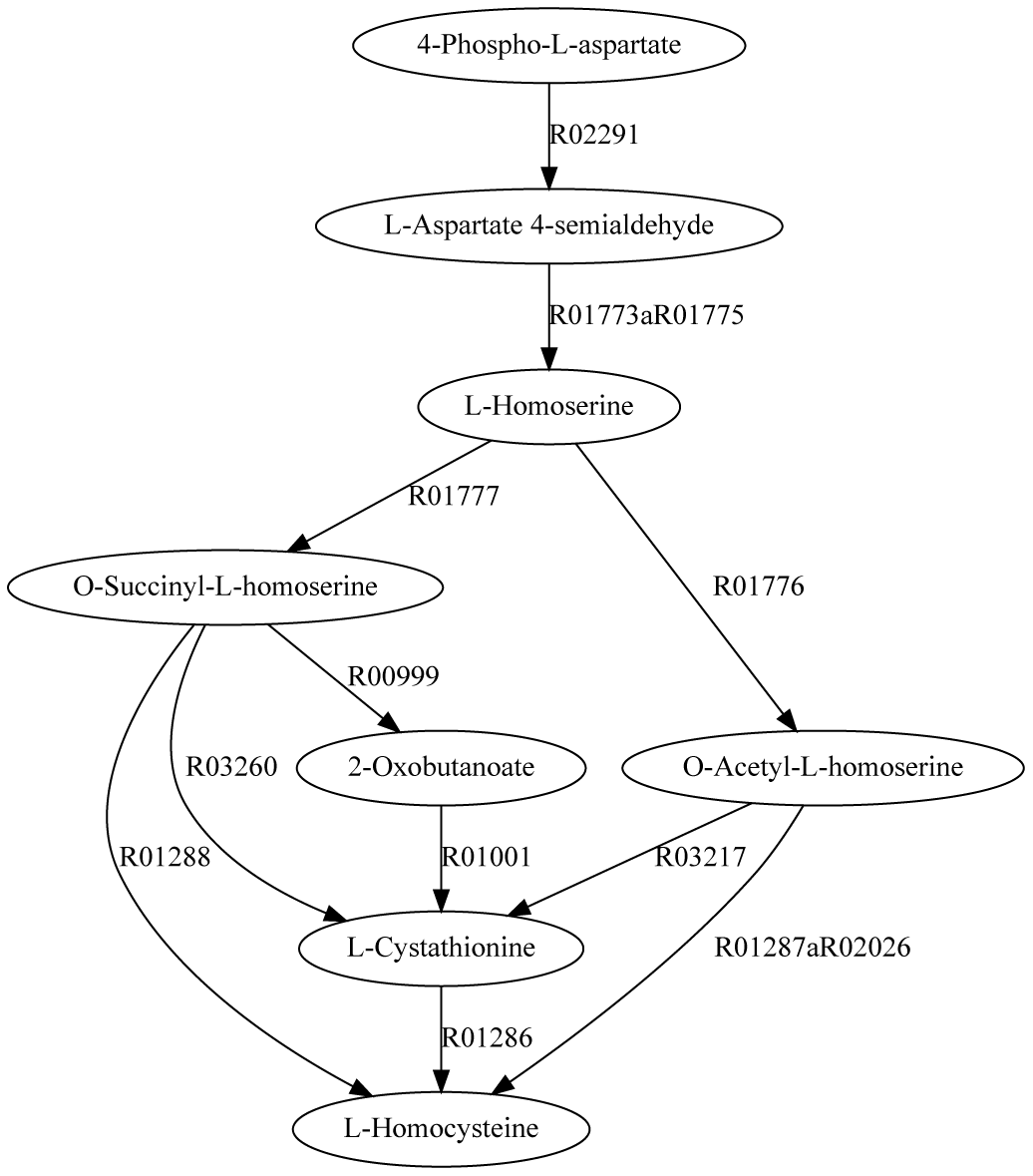
**

**Fig 23(S). The known pathway of 4-Phospho-L-aspartate to L-Homocysteine from KEGG rn00270.**

**
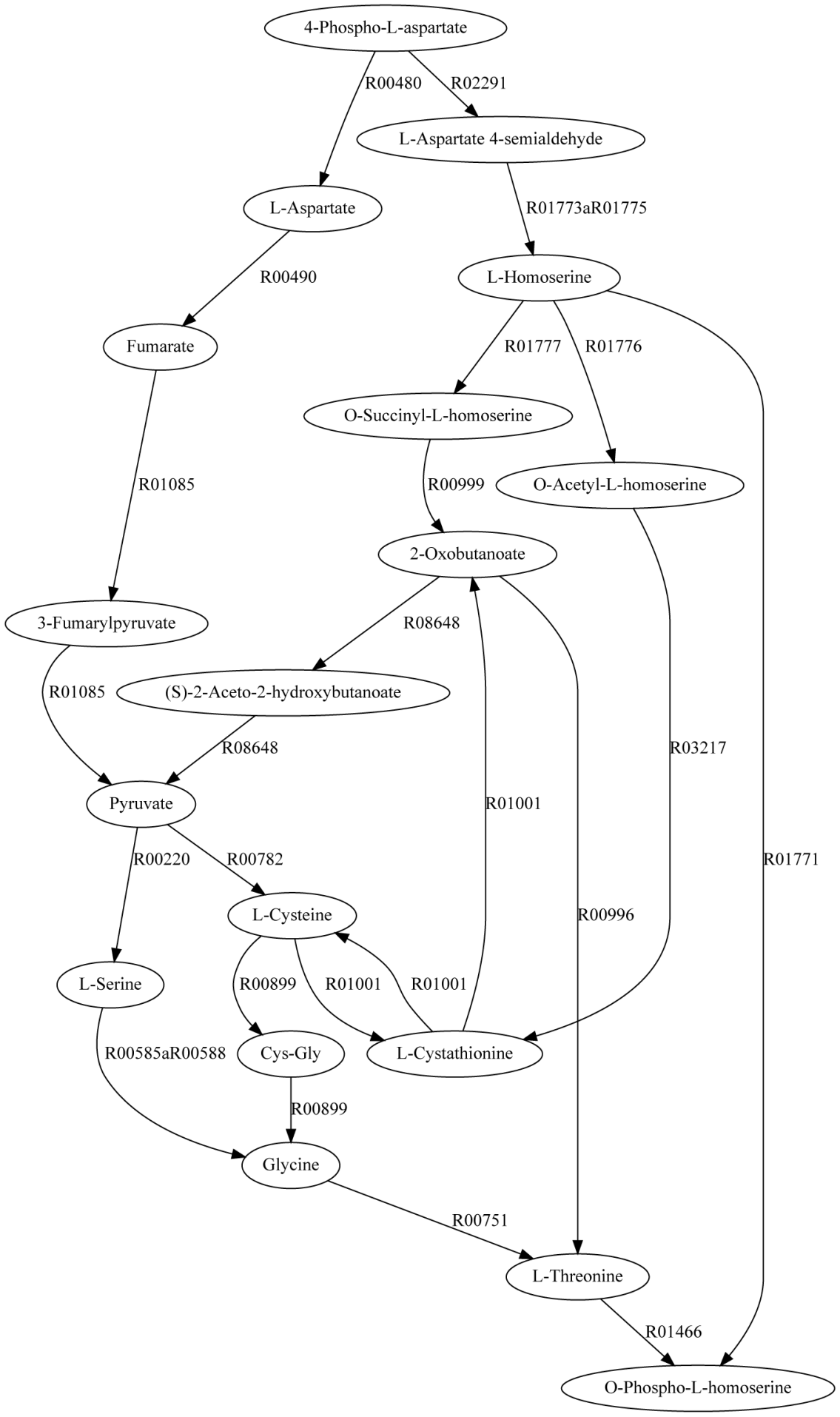
**

**Fig 24(S). The known pathway of 4-Phospho-L-aspartate to O-Phospho-L-homoserine from KEGG rn01100.**

**
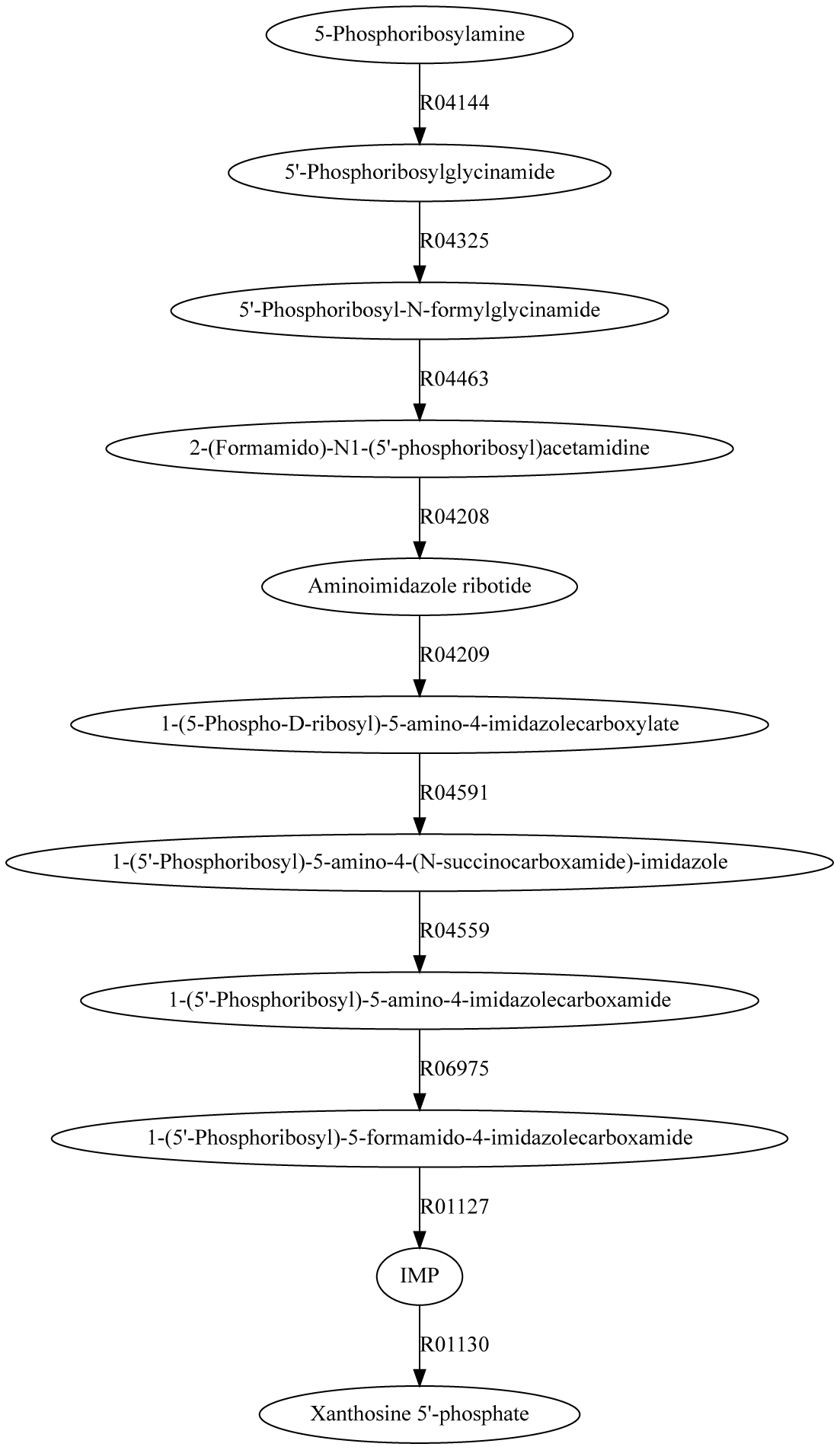
**

**Fig 25(S). The known pathway of 5-Phosphoribosylamine to Xanthosine 5’-phosphate from KEGG rn01100.**

**
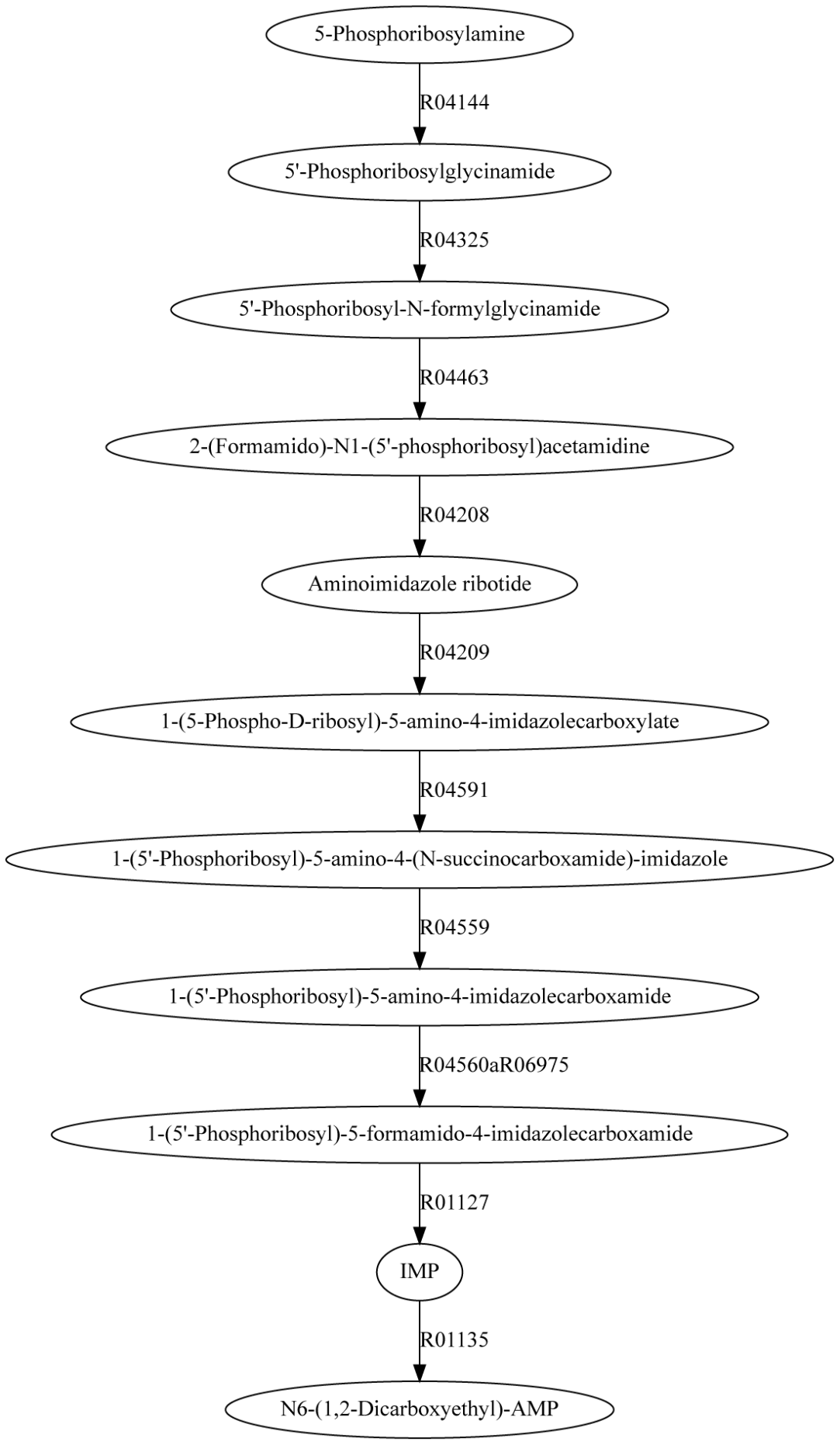
**

**Fig 26(S). The known pathway of 5-Phosphoribosylamine to N6-(1,2-Dicarboxyethyl)-AMP from KEGG rn01100.**

**
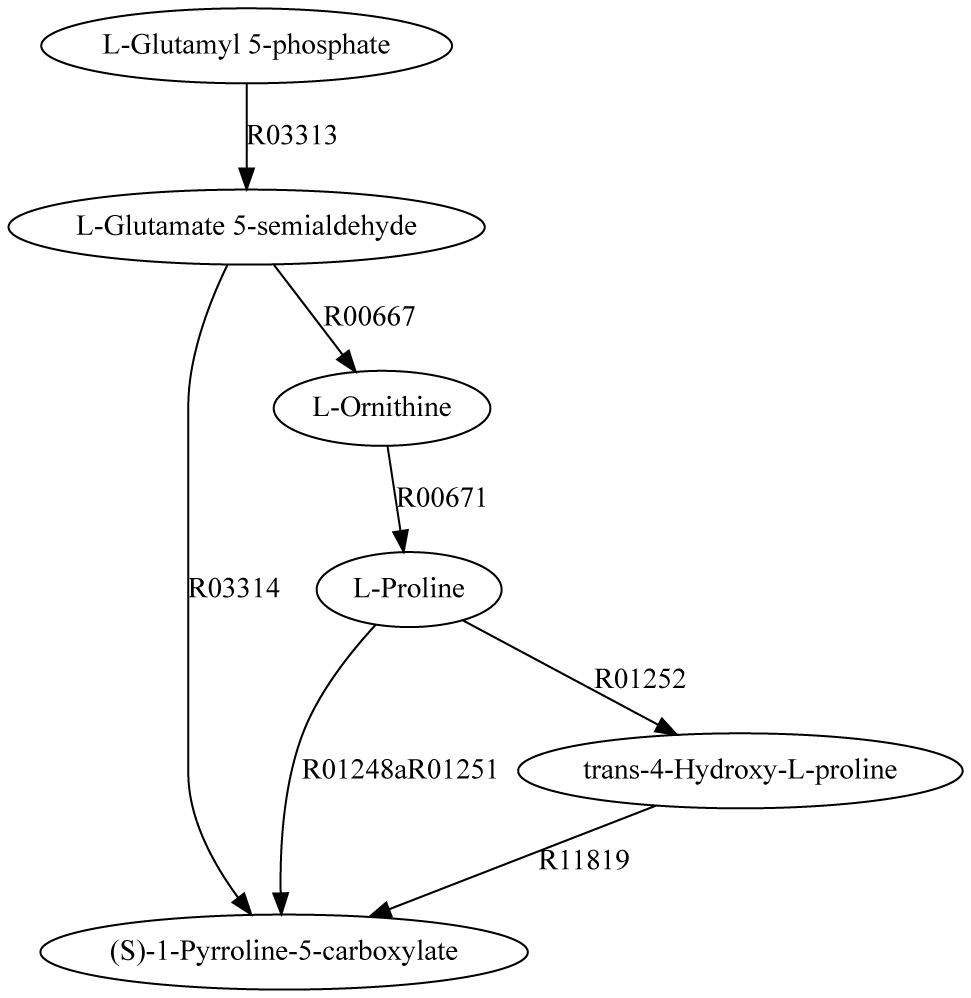
**

**Fig 27(S). The known pathway of L-Glutamyl 5-phosphate to (S)-l-Pyrroline-5-carboxylate from KEGG rn00330.**

**
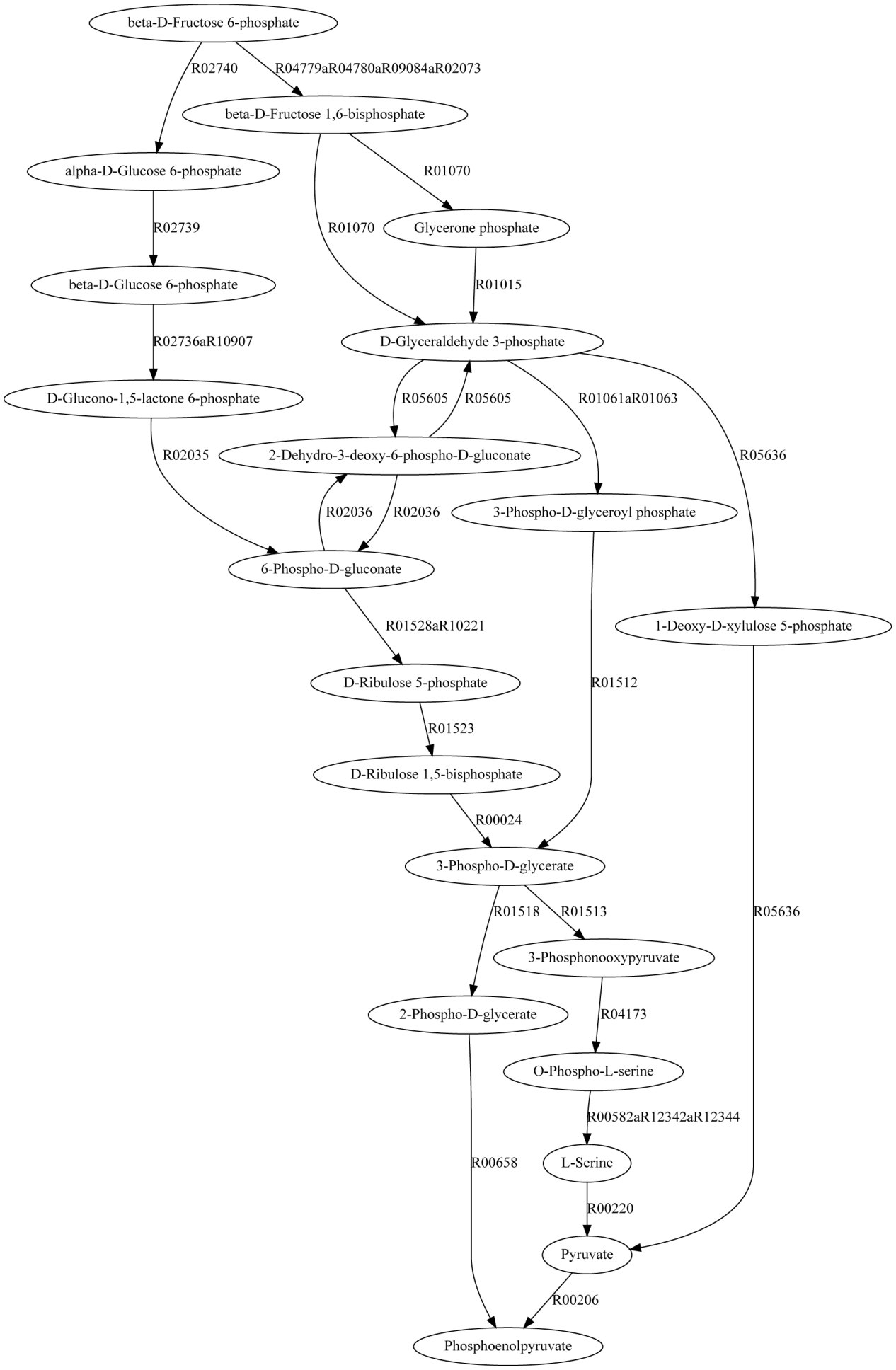
**

**Fig 28(S). The known pathway of beta-D-Fructose 6-phosphate to Phosphonenolpyruvate from KEGG rn01100.**

**
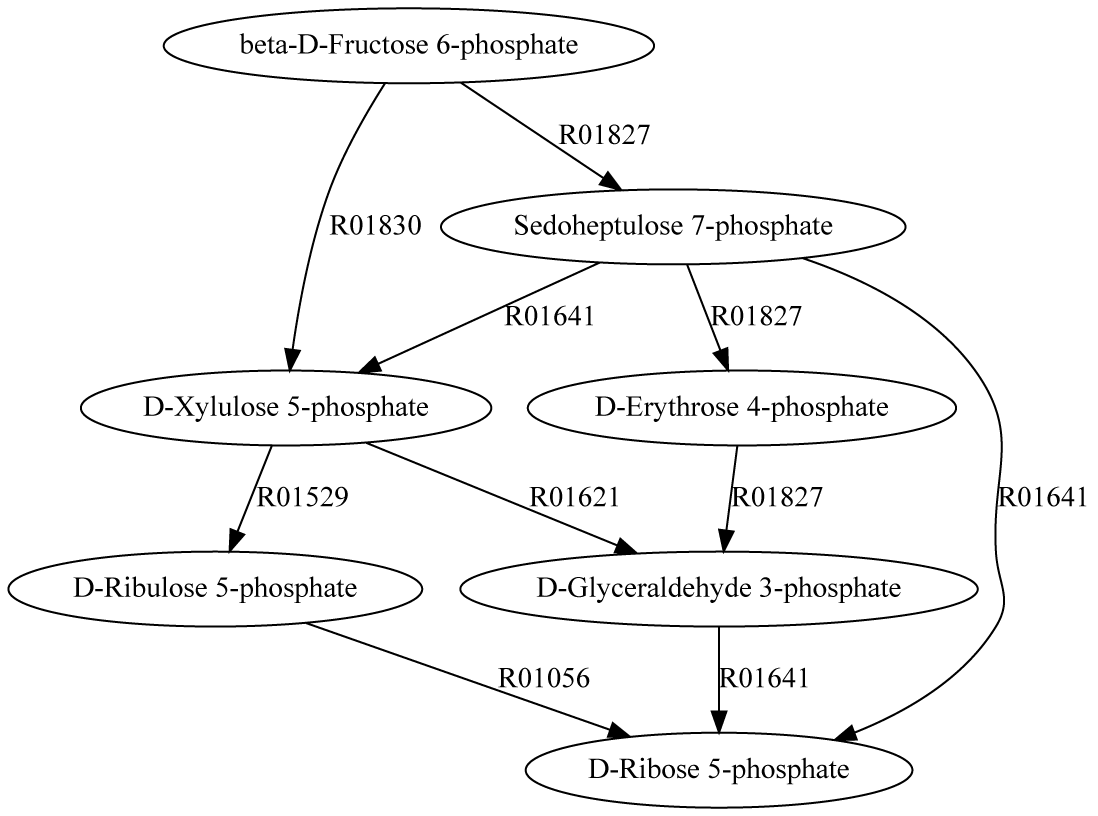
**

**Fig 29(S). The known pathway of beta-D-Fructose 6-phosphate to D-Ribose 5-phosphate from KEGG rn00030.**

**
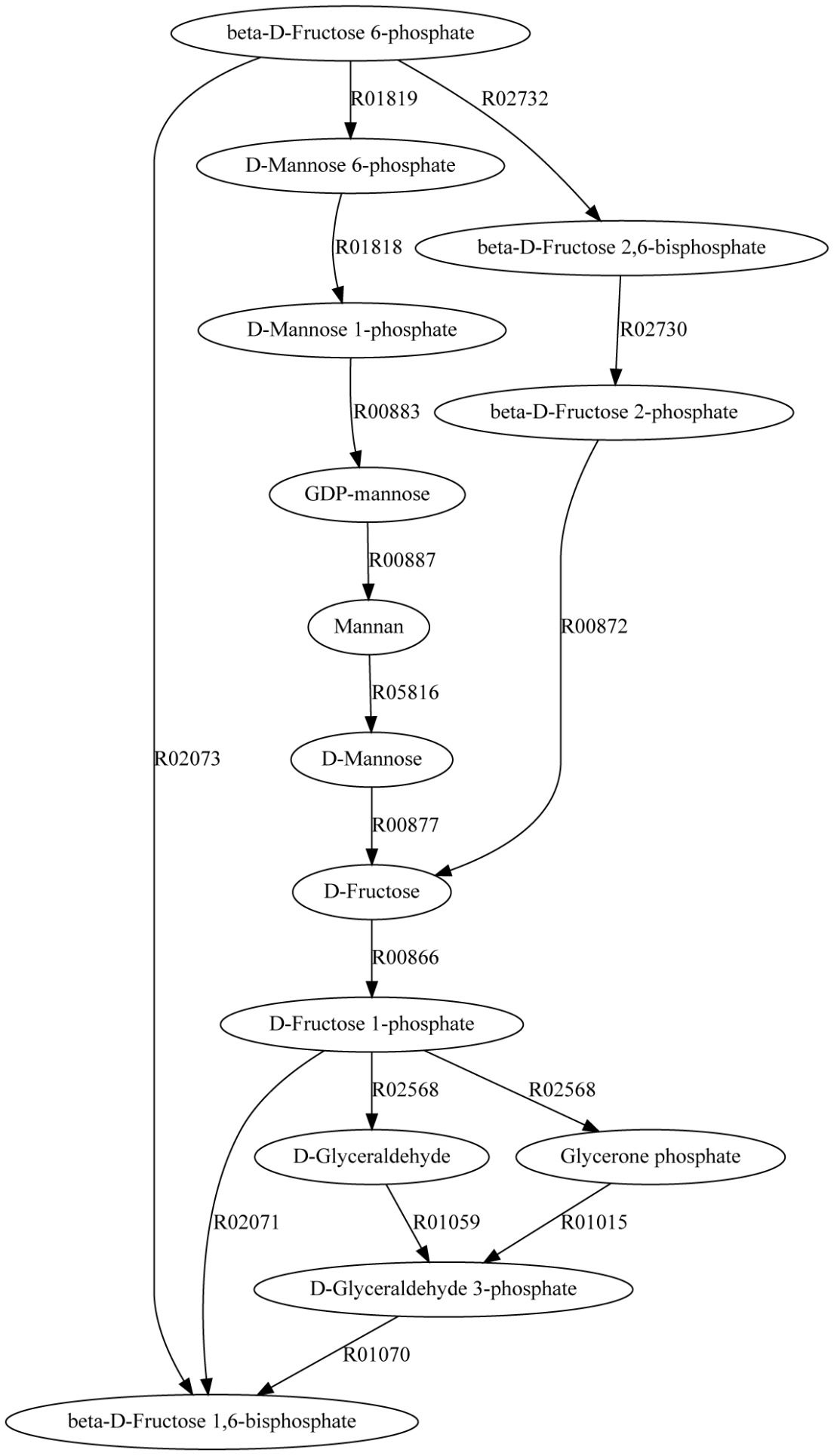
**

**Fig 30(S). The known pathway of beta-D-Fructose 6-phosphate to beta-D-Fructose 1,6-bisphosphate from KEGG rn01100.**
