# Supplementary material for: Finding branched pathways in metabolic network via atom group tracking
Source: PLoS Comput Biol. 2021 Feb 2;17(2):e1008676. doi: 10.1371/journal.pcbi.1008676 (PMC7880430; doi:10.1371/journal.pcbi.1008676)
Supplement: S2 Text — (DOCX) [file pcbi.1008676.s002.docx]

**Running parameters of the pathfinding methods**

**Table 1(S). Default parameters of BPAT-M**

| **Option** | **Description** | **value** |
| --- | --- | --- |
| k | Number of shortest paths computed in each step | 100000 |
|  |  |  |
| w | The algorithm performs a beam search with the fixed beam width | 500 |
|  |  |  |
| cluster | The number of pathways in each cluster | 2000 |
|  |  |  |
| carbon atoms | The number of carbons tracking for Seed Pathway | 2 |
|  |  |  |

**Table 2(S). Default parameters of BPAT-S**

| **Option** | **Description** | **value** |
| --- | --- | --- |
| k | Number of shortest paths computed in each step | 100000 |
|  |  |  |
| carbon atoms | The number of carbons tracking for Seed Pathway | Maximize Carbons |
|  |  |  |

**Table 3(S). Default parameters of Retrace**

| **Option** | **Description** | **value** |
| --- | --- | --- |
| -s | Source metabolites | *Required* |
| -t | Target metabolite | *Required* |
| -e | Atom graph edge weights: (u)niform, (s)cores, (a)toms | Uniform |
| -g | Greedy finish: set k=1 for search levels 2 and beyond | No |
| -i | Report incomplete pathways | No |
| -l | Maximum pathway size | Unbounded |
| -k | Number of shortest paths computed in each step | 50,1 |
| -m | Maximum search depth | 3 |
| -p | Prune atom graph | No pruning |
| -r | Reaction direction constraints file | - |
| -w | Minimum Z_O_ score requirement | 0 |

**Table 4(S). Default parameters of Phdseeker**

| **Option** | **Description** | **value** |
| --- | --- | --- |
| NCORES | Number of cores used to parallelize | 6 |
|  |  |  |
| Nants | Number of ants peranthill | 10 |
| rho | Evaporation rate | 0.1 |
| maxIterations | Maximum number of iterations | 1 |
| IterationsWithoutChanges | Minimum number of consecutive iterations without changes in the best solution | 2 |
|  |  |  |
|  |  |  |
| IterationsWithAlignedAnts | Number of consecutive iterations with all ants following the same solution | 2 |
|  |  |  |
| StrictInitialization | If "true", force the algorithm to use only feasible initial reactions from the provided substrates | FALSE |
|  |  |  |
| AllowExternalCompounds | If "true", allows the algorithm to include as freely available compounds all those which cannot be synthesized by any reaction | TRUE |
|  |  |  |
|  |  |  |
|  |  |  |
| Verbose | If "true", a detail of the searching process is shown. | TRUE |
|  |  |  |
| abundant | KEGG ID of the hub metabolites | C00001, C00002, C00003, C00004, C00005, C00006, C00007, C00008, C00009, C00010, C00080 |
|  |  |  |
|  |  |  |
|  |  |  |
|  |  |  |
|  |  |  |

**Table 5(S). Default parameters of MetQuest**

| **Option** | **Description** | **value** |
| --- | --- | --- |
| β | The sub-networks within a size cut-of | 15 |
|  |  |  |
| SBML | Model(s) of metabolic networks # XML files of the metabolic networks(COBRA-compatible) | Recon3D.xml, iRC1080.xml, iND750.xml, iJO1366.xml |
|  |  |  |
| seed_mets | The seed metabolites | glc__D_eiJO1366, ACP_ciJO1366, adp_ciJO1366, amp_ciJO1366, atp_ciJO1366, co2_ciJO1366, coa_ciJO1366, h2o_ciJO1366, h2o_eiJO1366, h2o_piJO1366, h_ciJO1366, nad_ciJO1366, nadh_ciJO1366, nadp_ciJO1366, nadph_ciJO1366, pi_ciJO1366, pi_piJO1366 ppi_c |
| source_mets | The source metabolites | *Required* |
| target_mets | The target metabolites | *Required* |
