# Supplementary material for: Finding branched pathways in metabolic network via atom group tracking
Source: PLoS Comput Biol. 2021 Feb 2;17(2):e1008676. doi: 10.1371/journal.pcbi.1008676 (PMC7880430; doi:10.1371/journal.pcbi.1008676)
Supplement: S3 Text — (DOCX) [file pcbi.1008676.s003.docx]

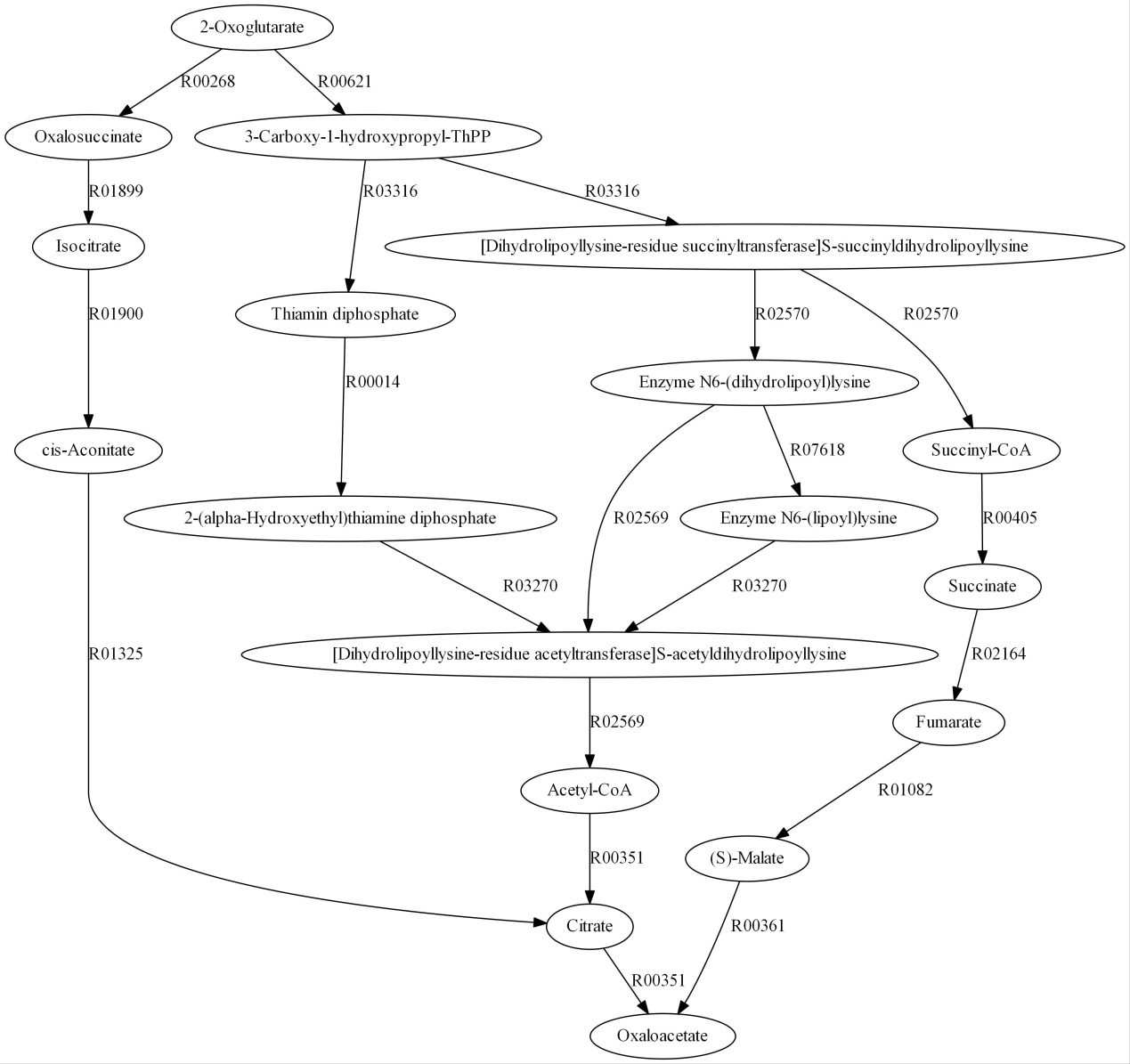
**Fig 1(S). Organism-specific pathway 1: 2-Oxoglutarate to Oxaloacetate**


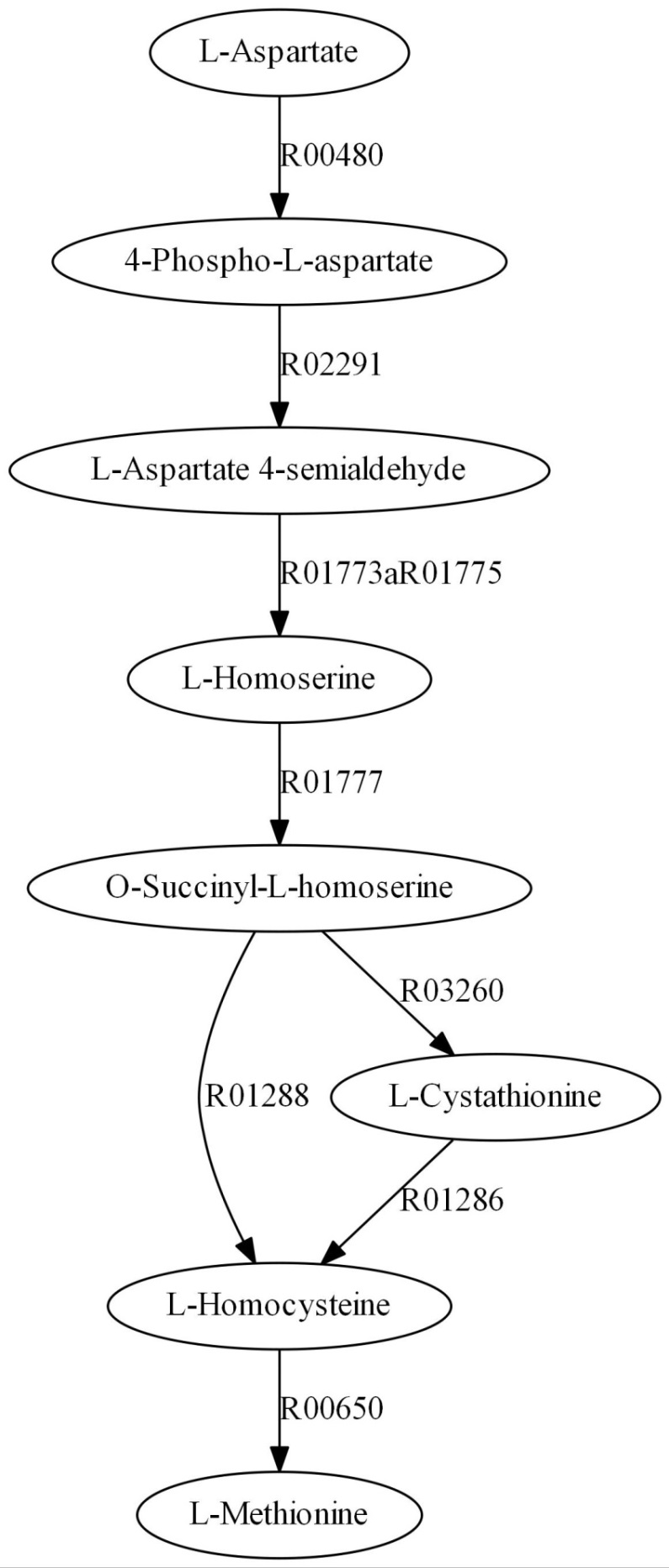


**Fig 2(S). Organism-specific pathway 2: L-Aspartate to L-Methionine**


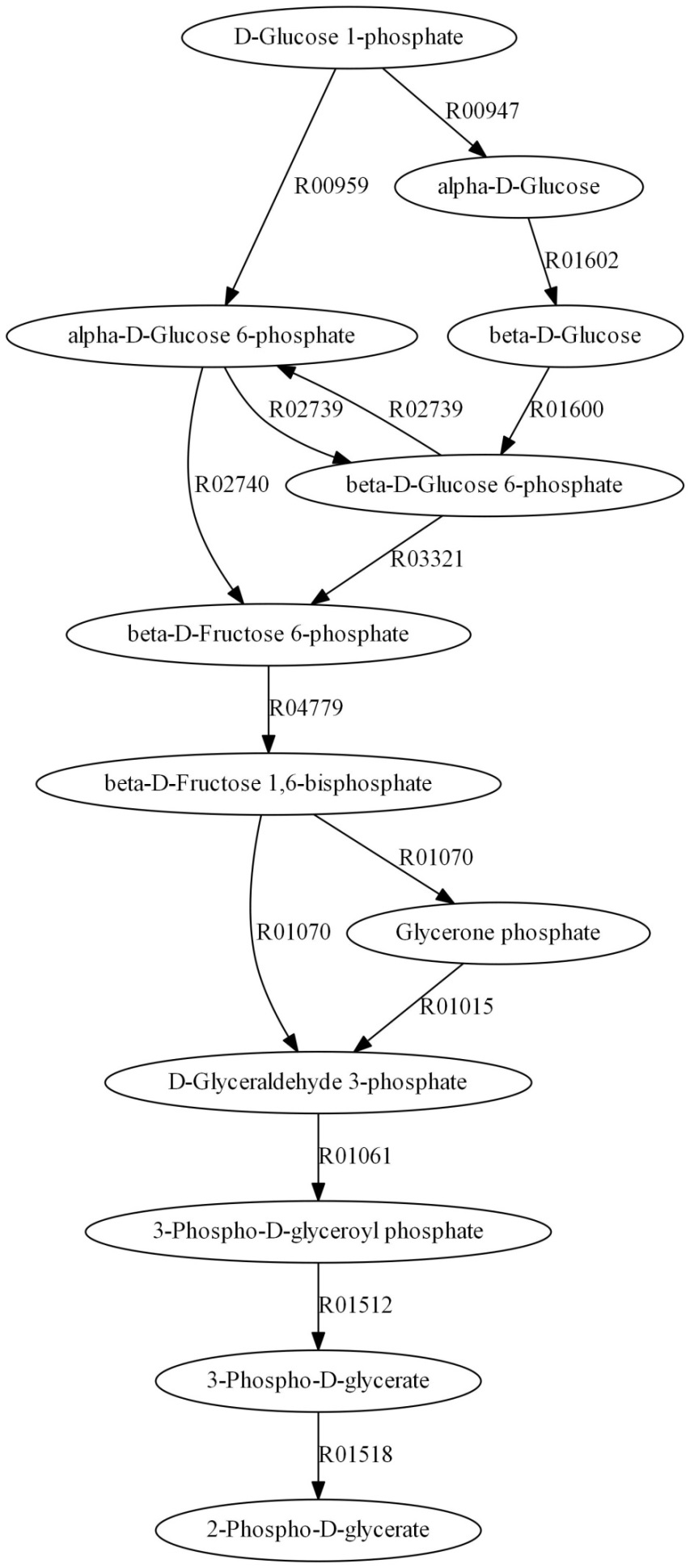


**Fig 3(S). Organism-specific pathway 3: D-Glucose 1-phsophate to 2-Phospho-D-glycerate**


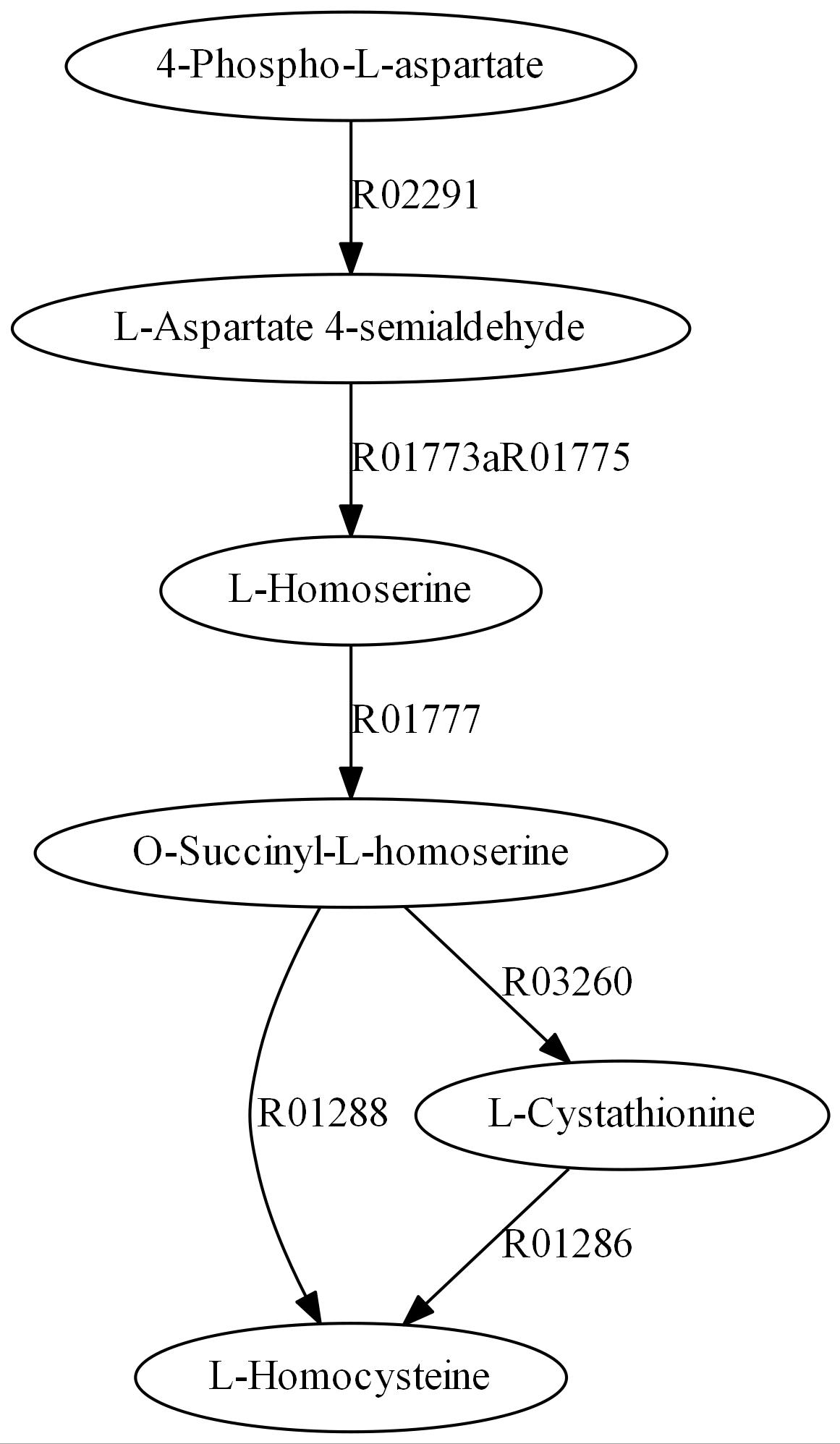


**Fig 4(S). Organism-specific pathway 4: 4-Phospho-L-aspartate to L-Homocysteine**
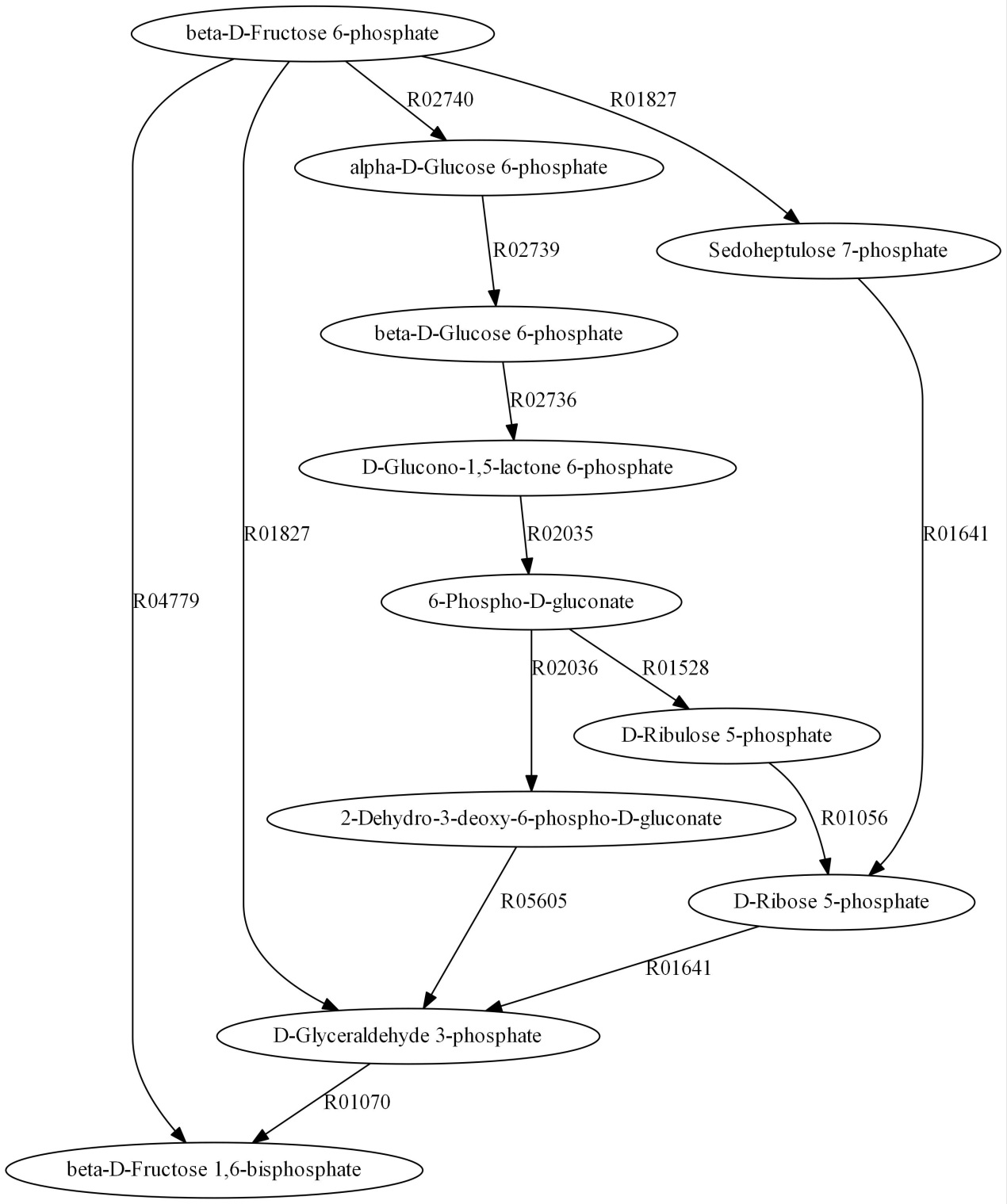


**Fig 5(S). Organism-specific pathway 5: beta-D-Fructose 6-phosphate to Phosphonenolpyruvate**


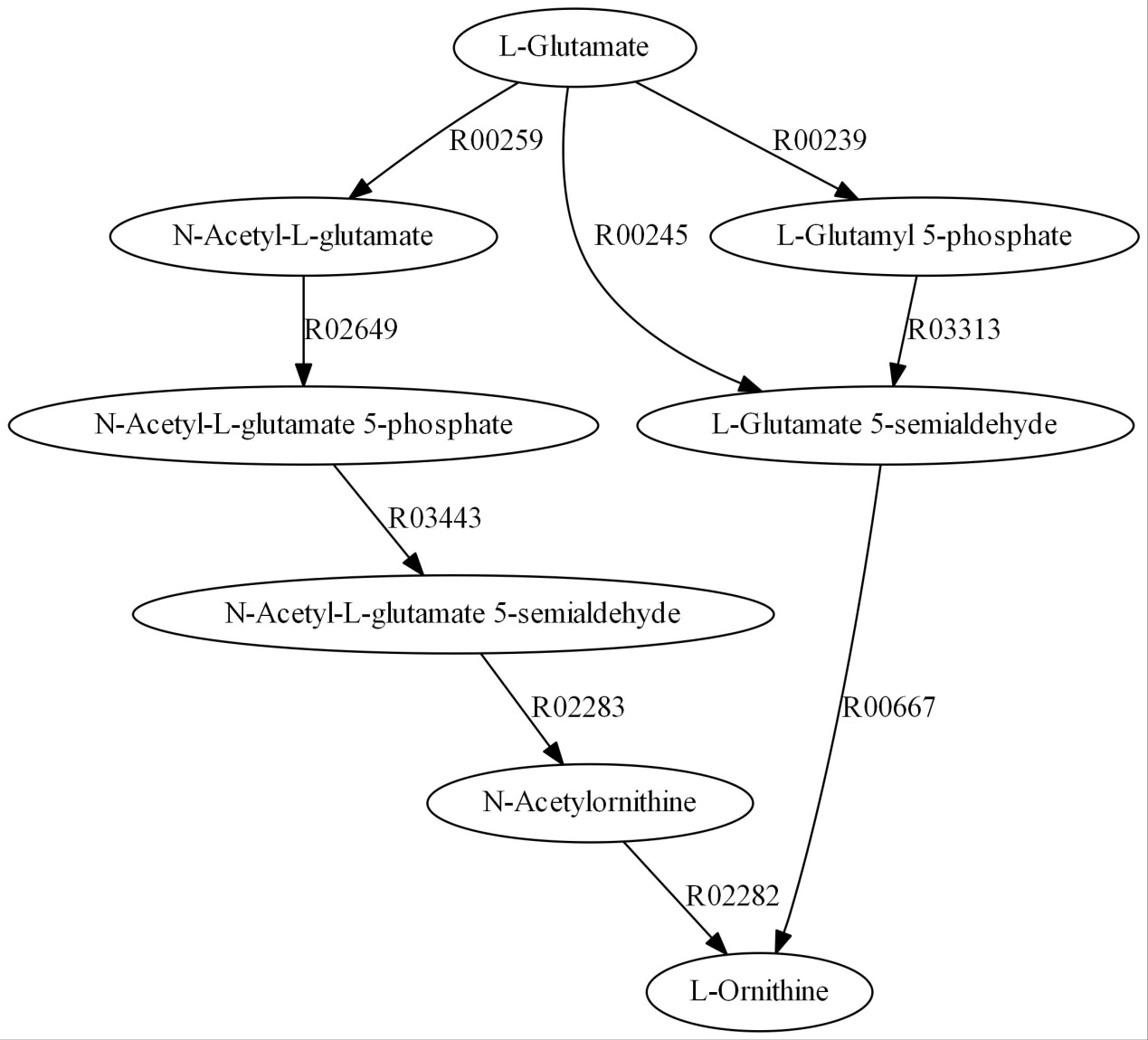
 **Fig 6(S). Organism-specific pathway 6: L-Glutamate to L-Ornithine**
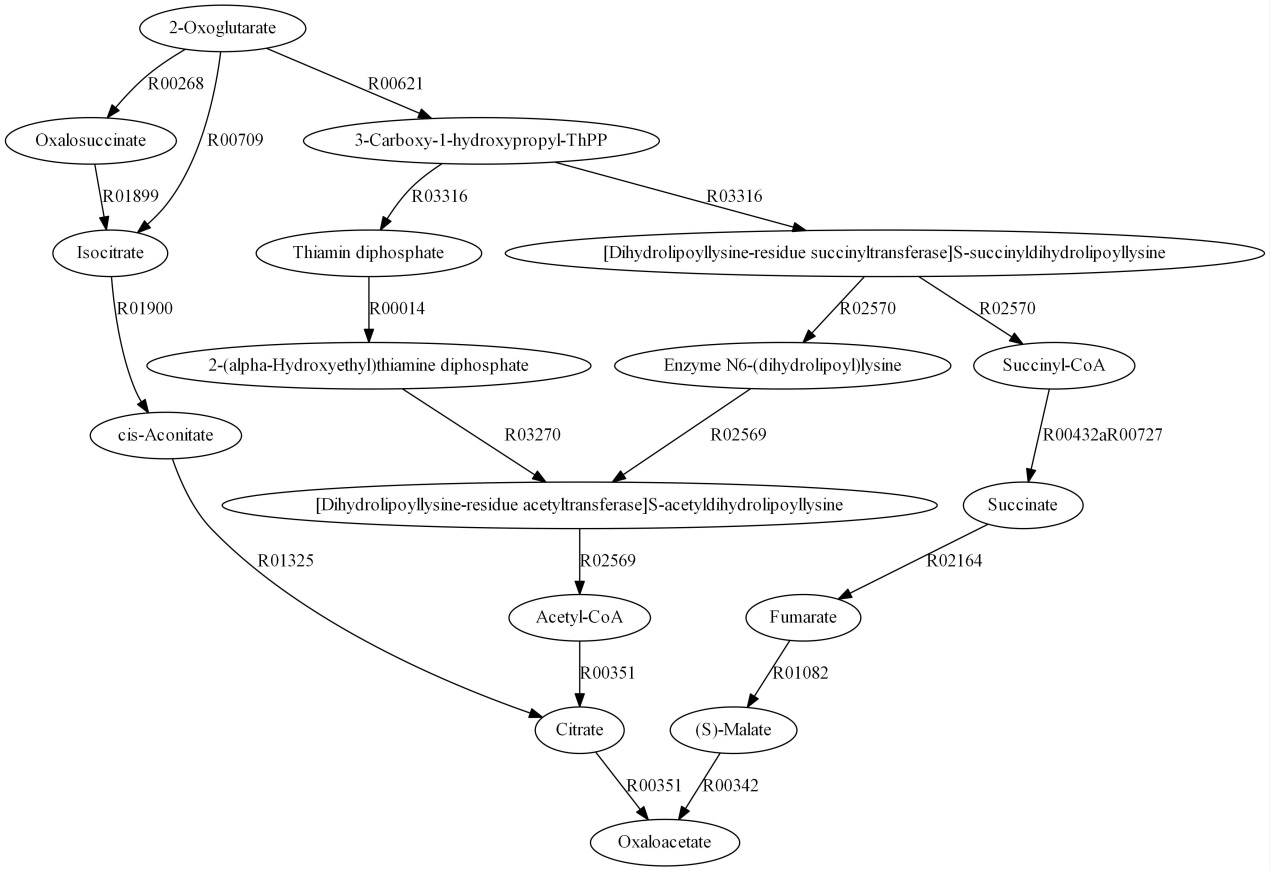
**Fig 7(S). Organism-specific pathway 7: 2-Oxoglutarate to Oxaloacetate**


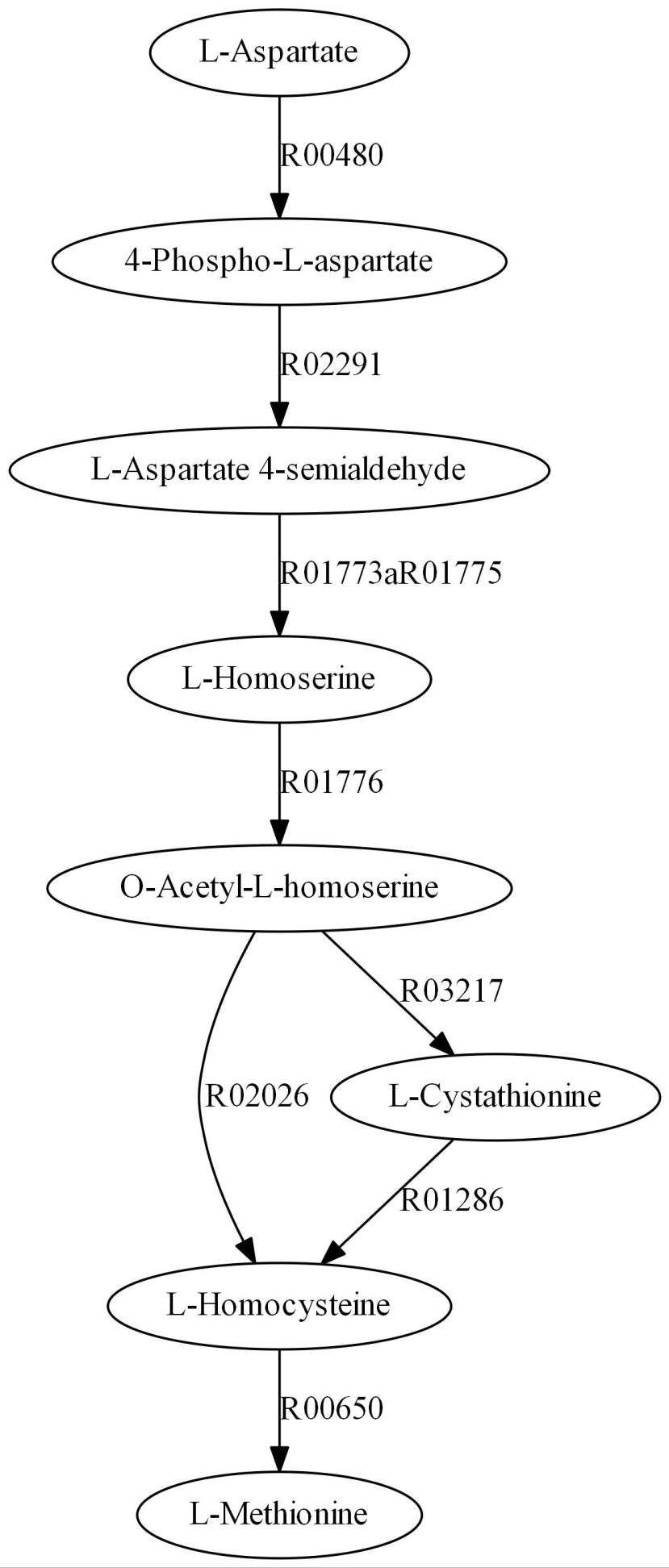


**Fig 8(S). Organism-specific pathway 8: L-Aspartate to L-Methionine**
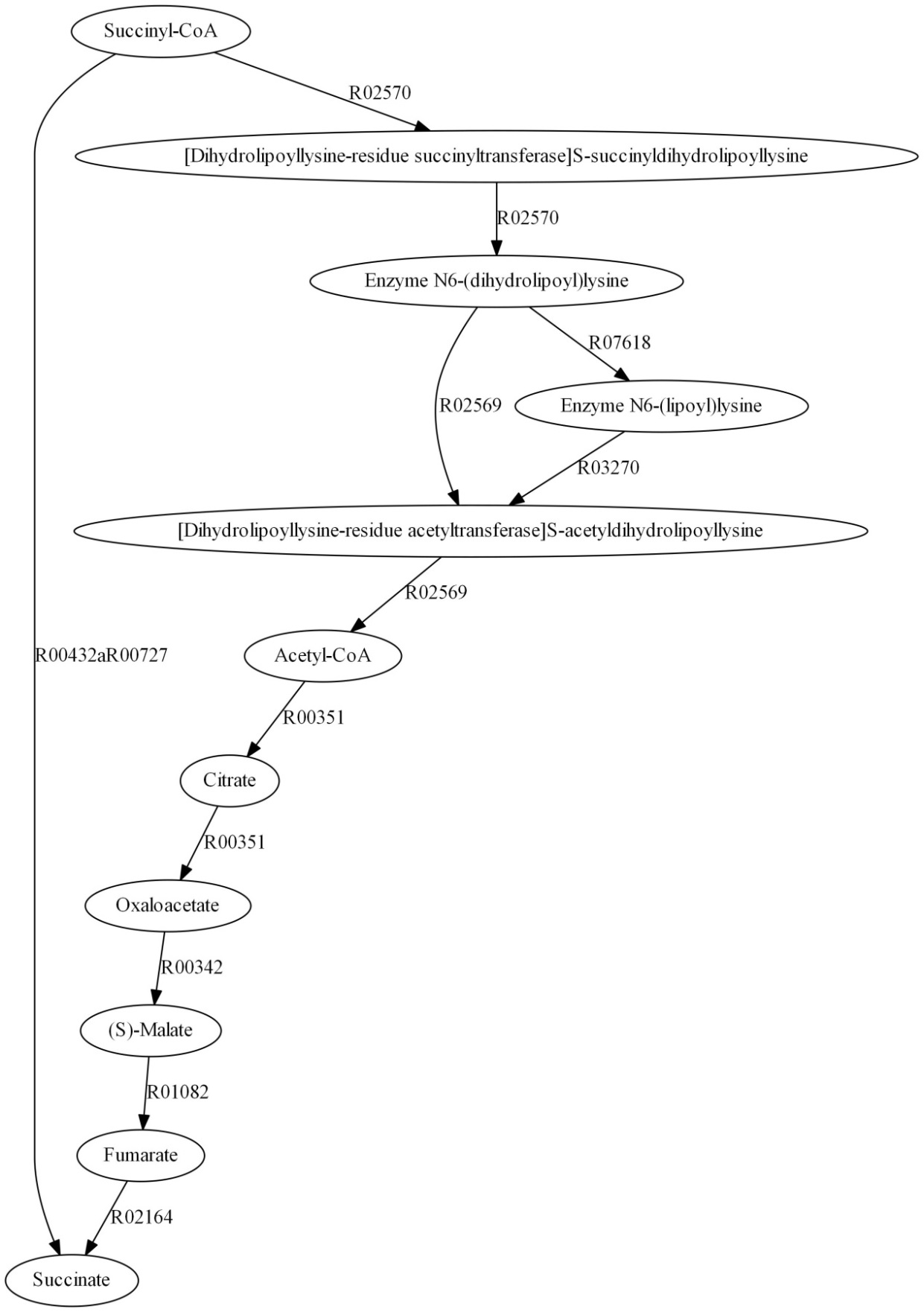
 **Fig 9(S). Organism-specific pathway 9: Succinyl-CoA to Succinate**


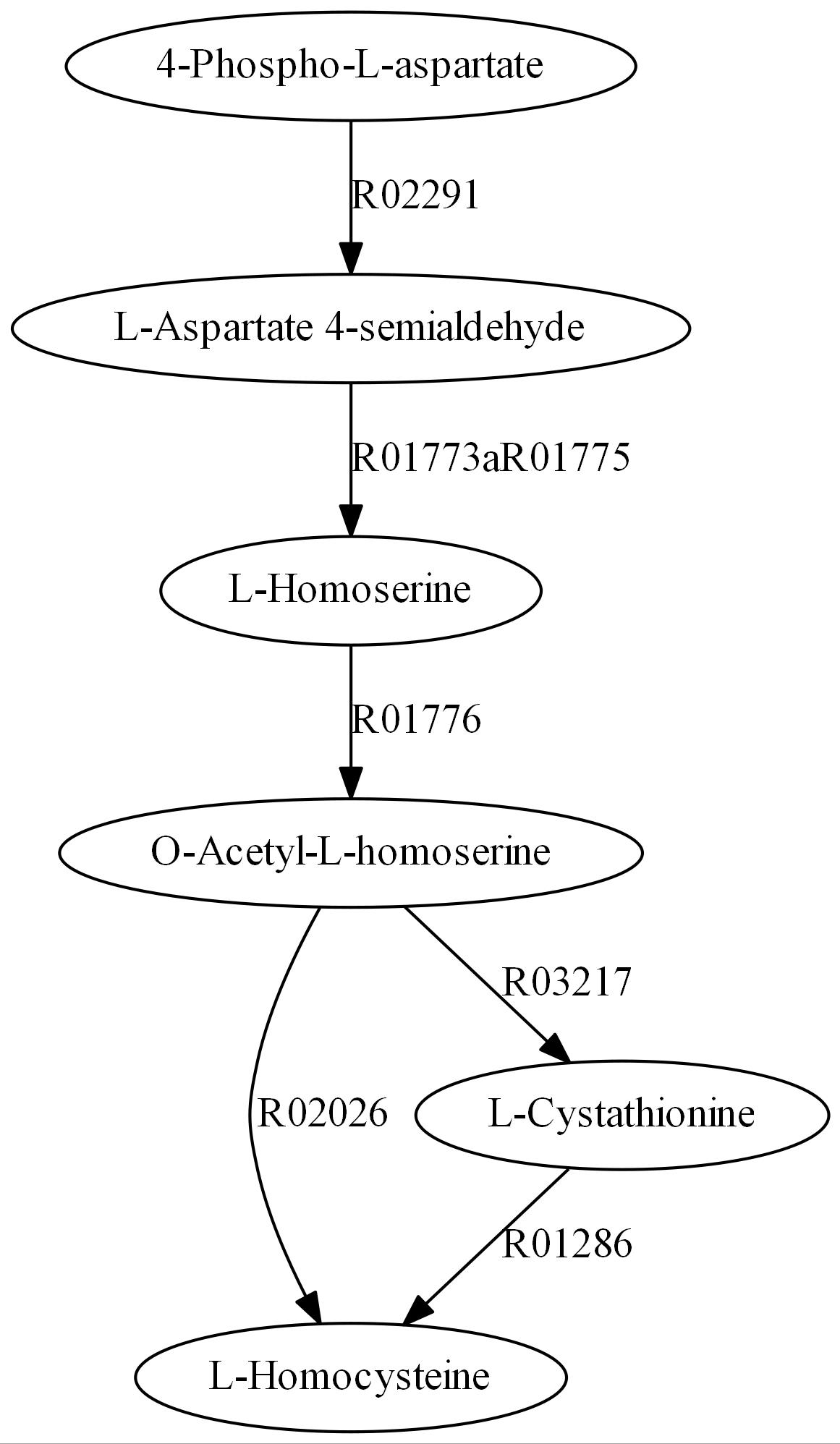


**Fig 10(S). Organism-specific pathway 10: 4-Phospho-L-aspartate to L-Homocysteine**


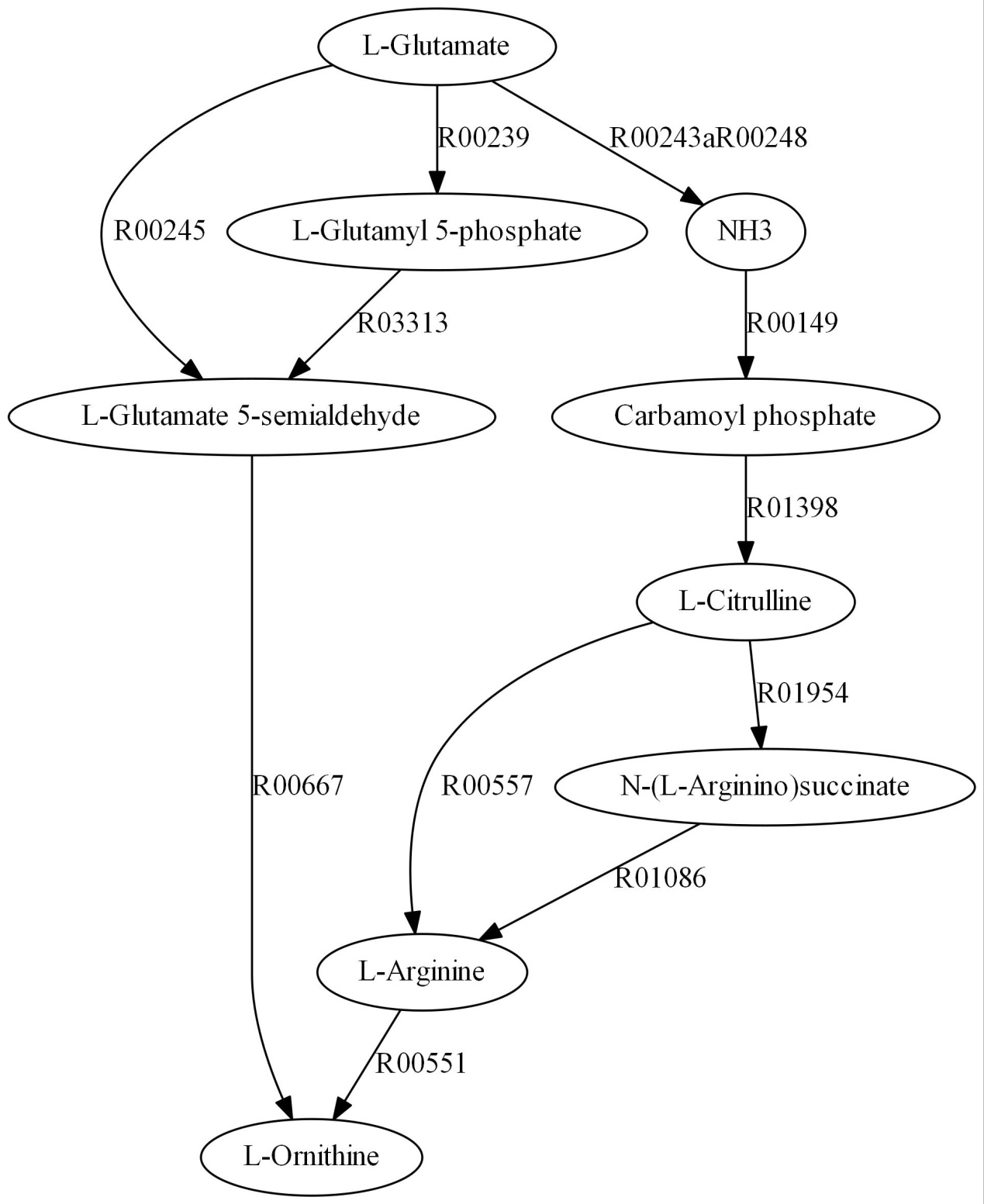
**Fig 11(S). Organism-specific pathway 11: L-Glutamate to L-Ornithine**
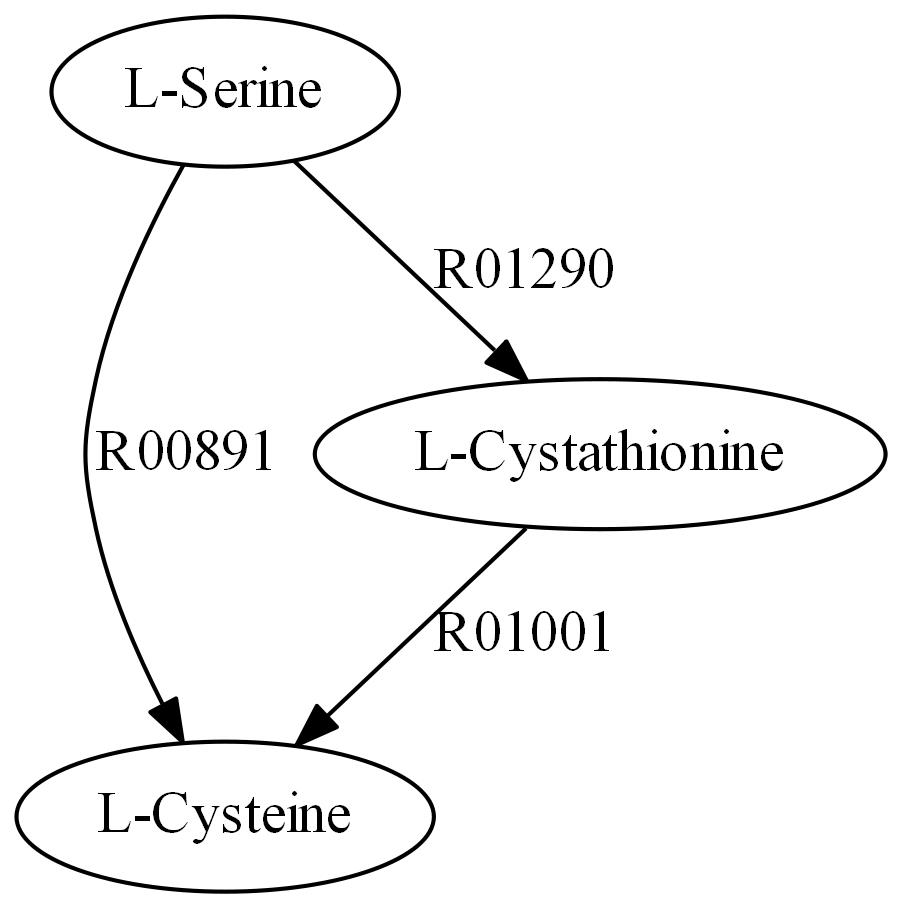
 **Fig 12(S). Organism-specific pathway 12:**

**L-Serine to L L-Cysteine**
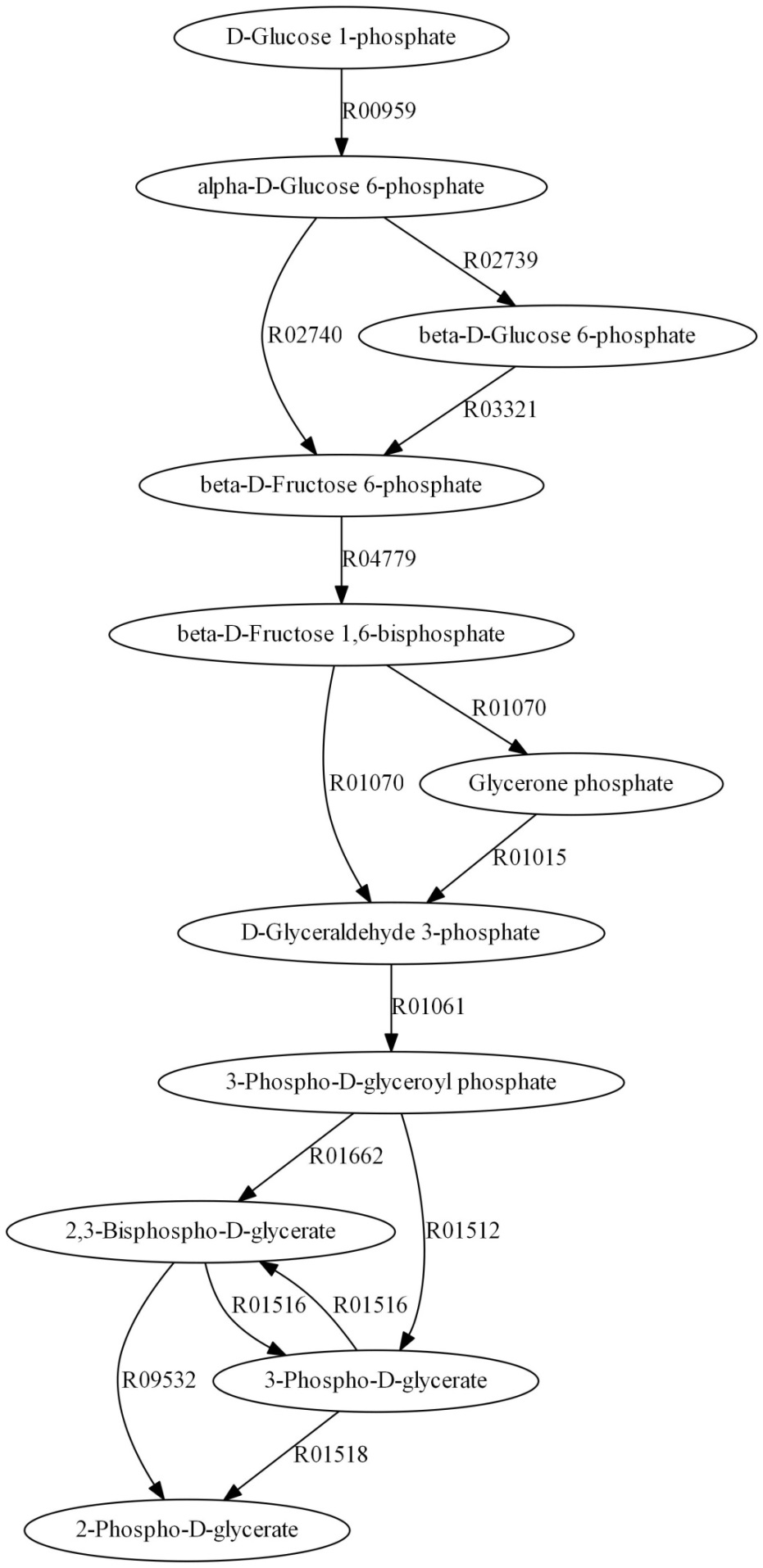


**Fig 13(S). Organism-specific pathway 13: D-Glucose 1-phsophate to 2-Phospho-D-glycerate**
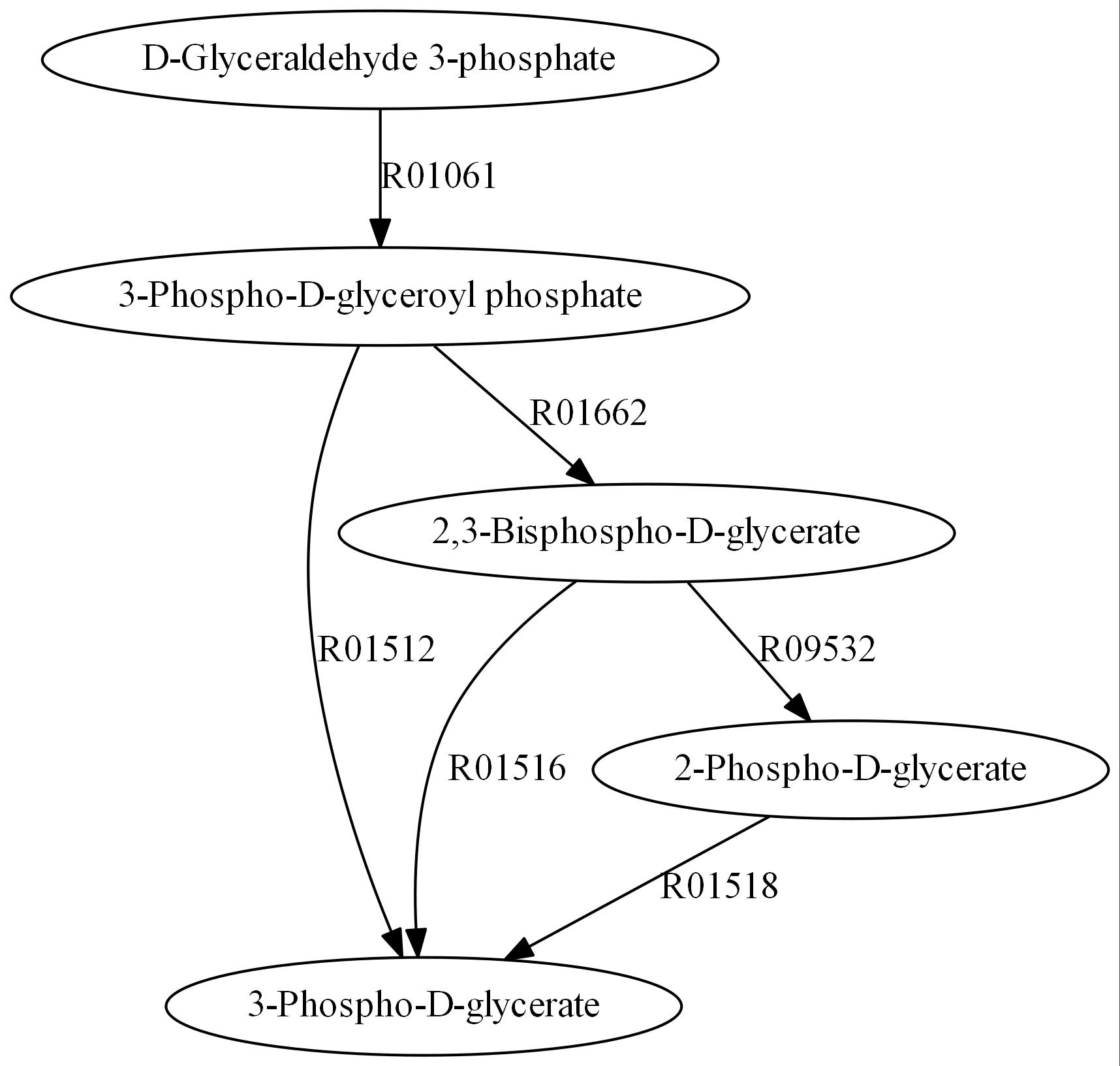
 **Fig 14(S). Organism-specific pathway 14: D-Glyceraldehyde 3-phosphate to 3-Phospho-D-glycerate**


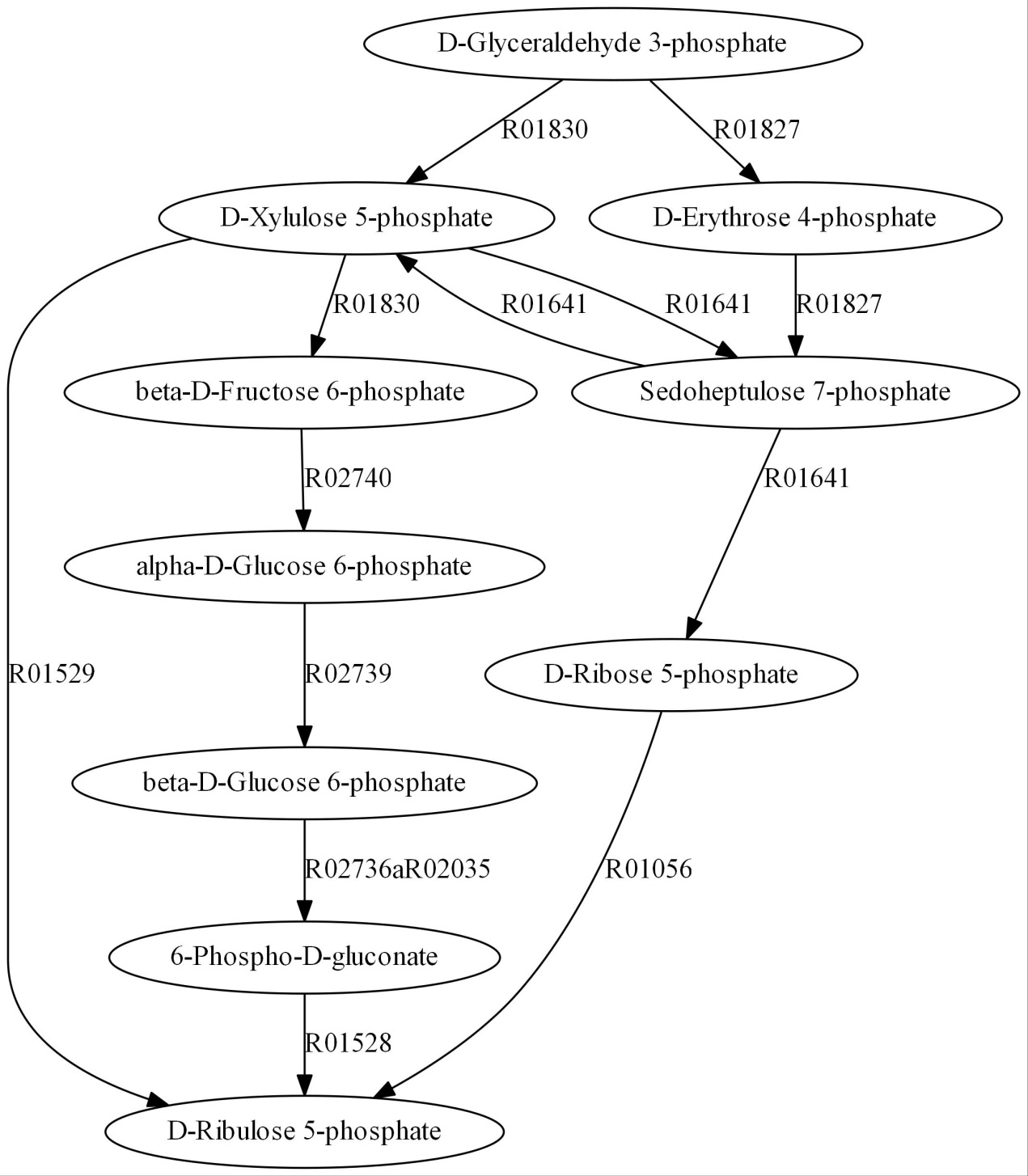
 **Fig 15(S). Organism-specific pathway 15: D-Glyceraldehyde 3-phosphate to D-Ribulose 5-phosphate**
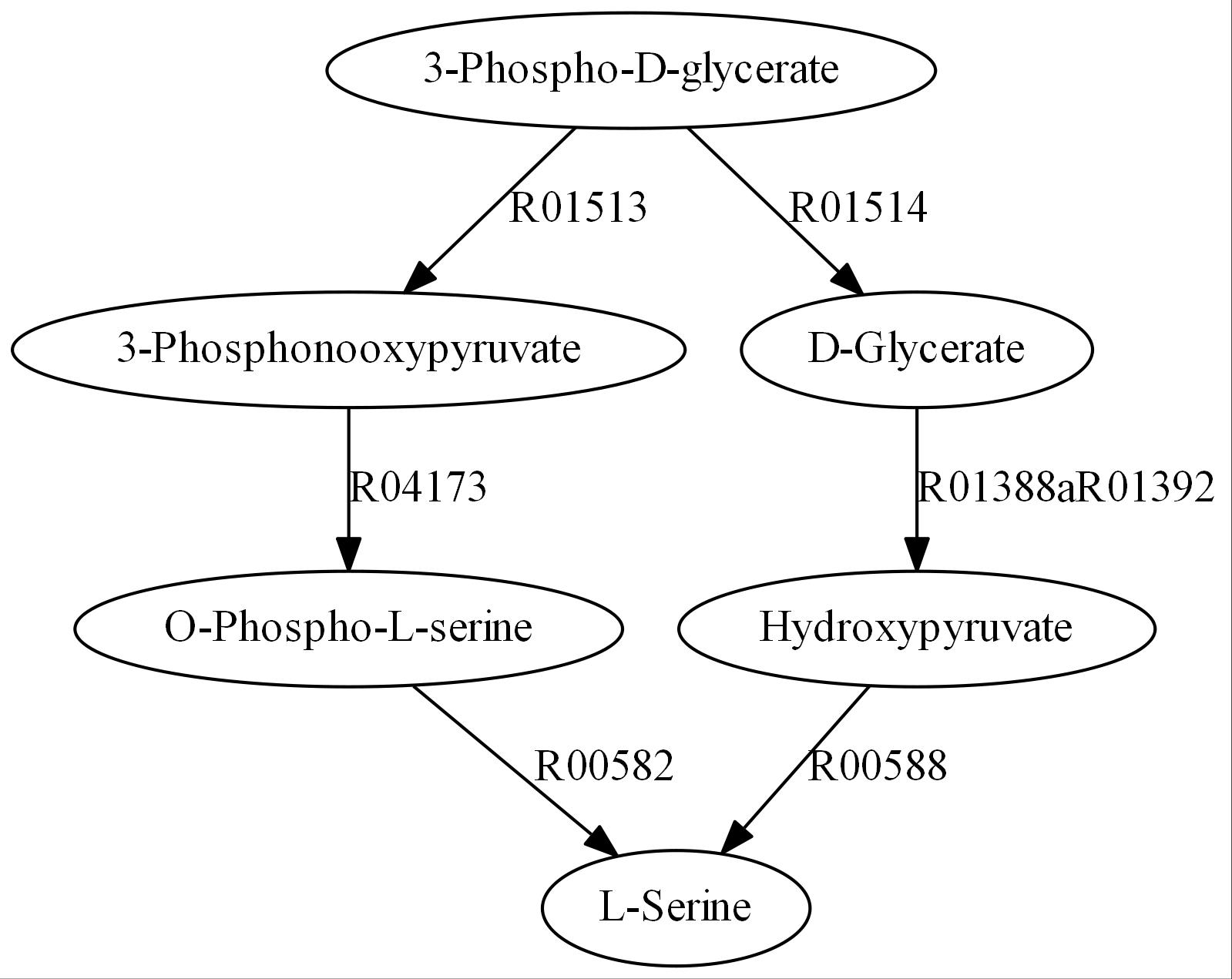
 **Fig 16(S). Organism-specific pathway 16: 3-Phospho-D-glycerate to L-Serine**


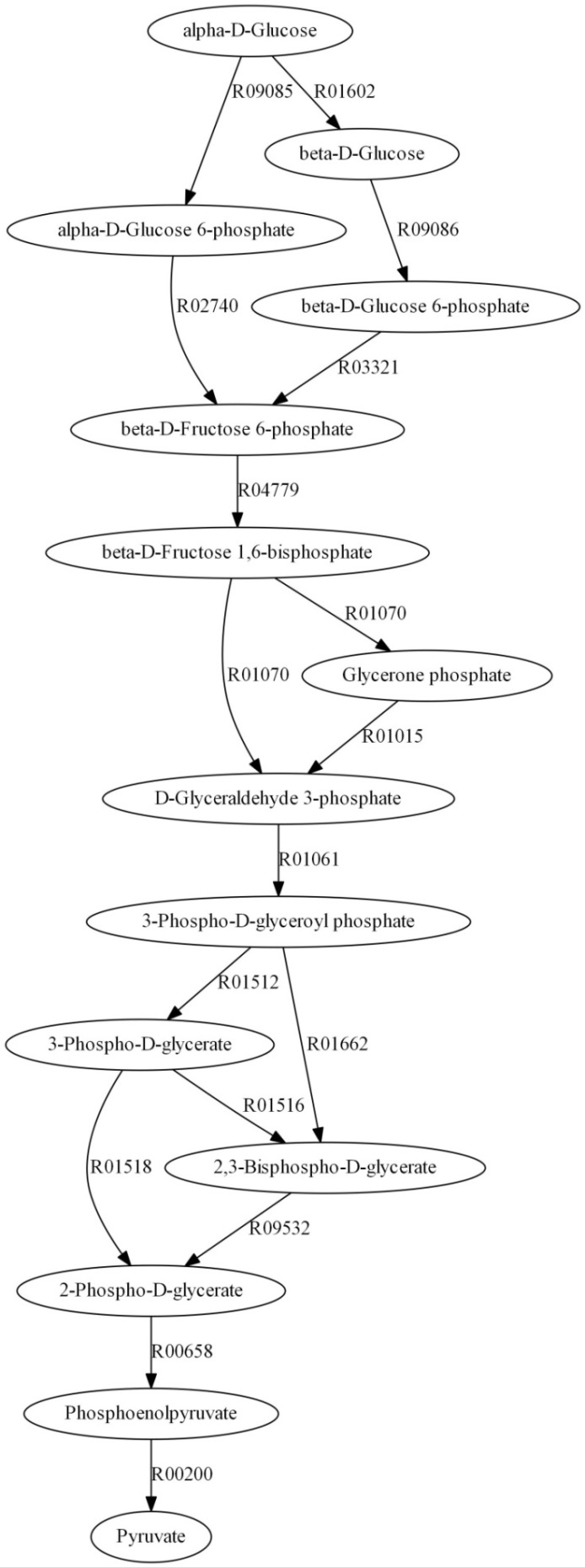


**Fig 17(S). Organism-specific pathway 17: alpha-D-Glucose to Pyruvate**
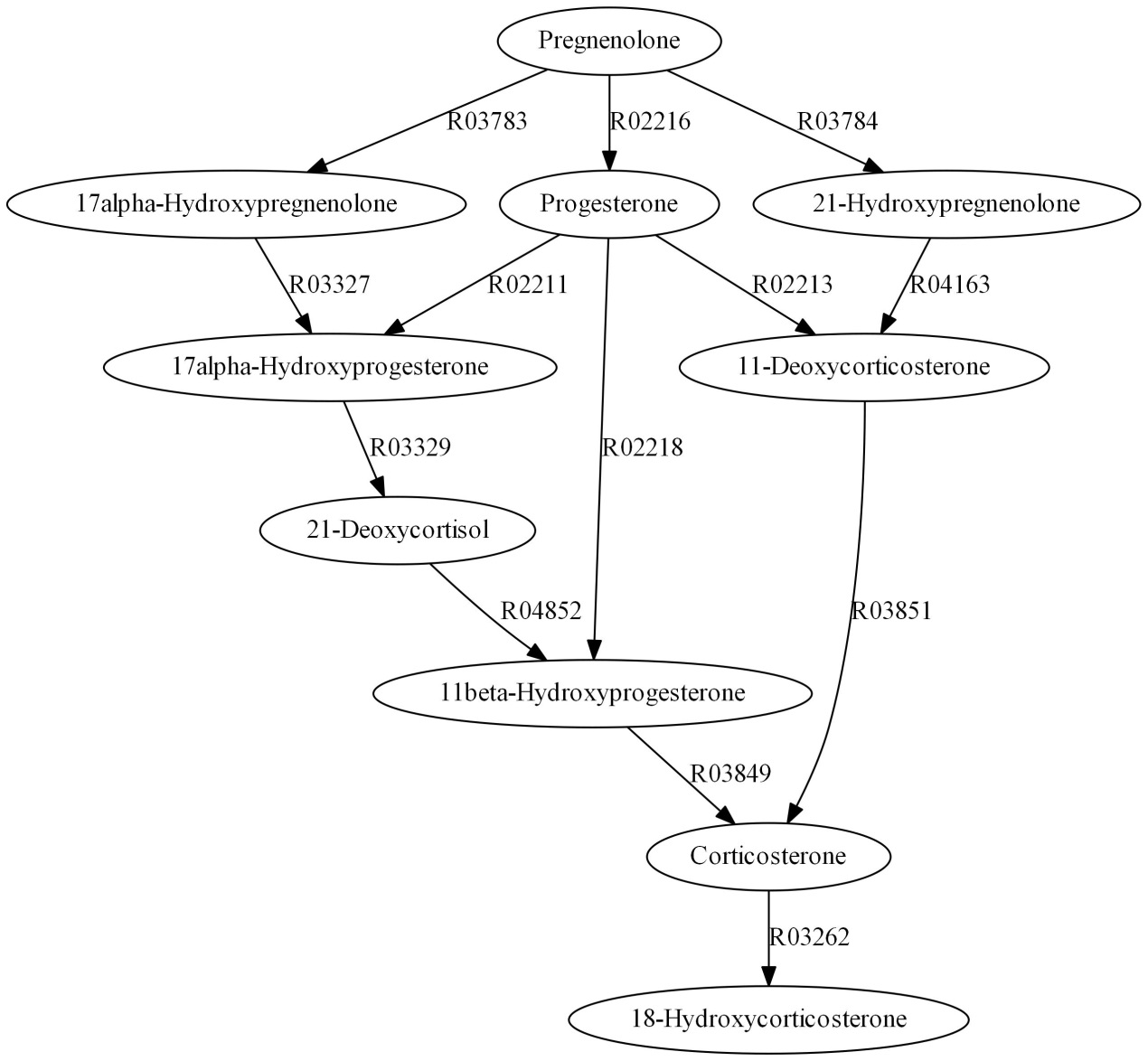
 **Fig 18(S). Organism-specific pathway 18: Pregnenolone to 18-Hydroxycorticosterone**
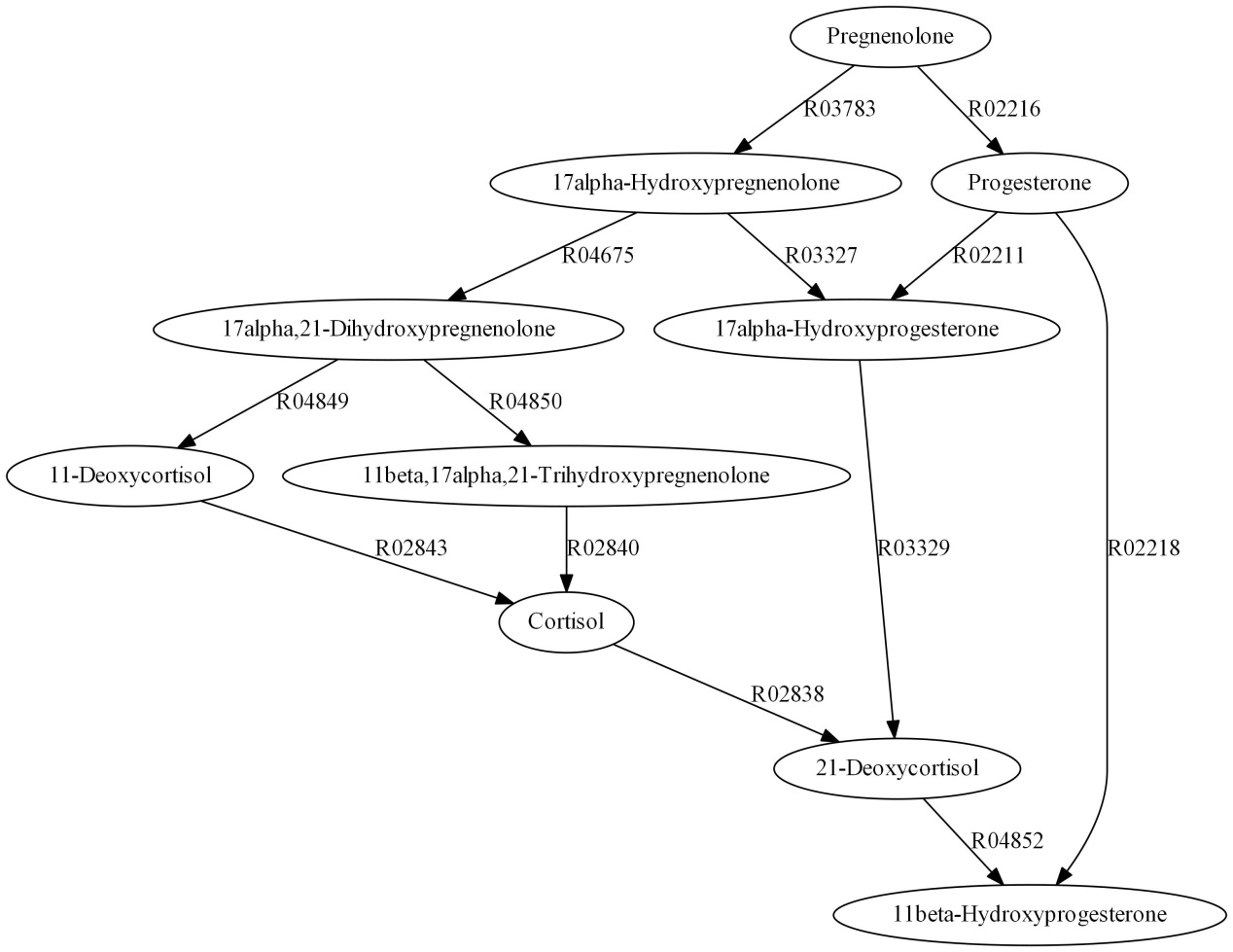
 **Fig 19(S). Organism-specific pathway 19: Pregnenolone to 11 beta-Hydroxyprogesterone**
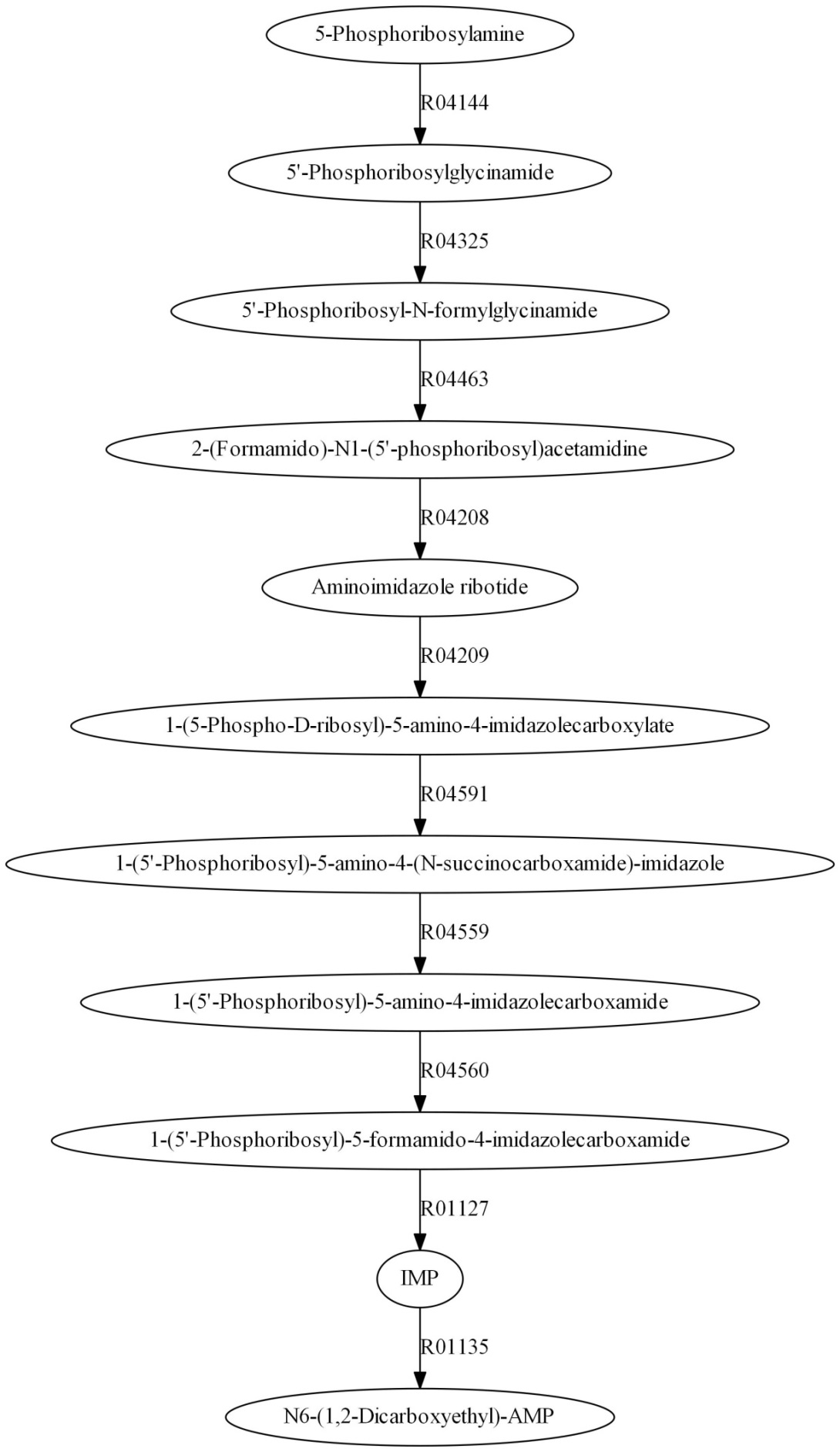


**Fig 20(S). Organism-specific pathway 20: 5-Phosphoribosylamine to N6-(1,2-Dicarboxyethyl)-AMP**
